# Encapsulation within a coordination cage modulates the reactivity of redox-active dyes

**DOI:** 10.1038/s42004-022-00658-8

**Published:** 2022-03-30

**Authors:** Oksana Yanshyna, Michał J. Białek, Oleg V. Chashchikhin, Rafal Klajn

**Affiliations:** 1 Department of Organic Chemistry, Weizmann Institute of Science, Rehovot, 76100 Israel; 2 Department of Chemistry, University of Wrocław, 14 F. Joliot-Curie St, 50383 Wrocław, Poland

**Keywords:** Self-assembly, Molecular capsules, Coordination chemistry

## Abstract

Confining molecules within well-defined nanosized spaces can profoundly alter their physicochemical characteristics. For example, the controlled aggregation of chromophores into discrete oligomers has been shown to tune their optical properties whereas encapsulation of reactive species within molecular hosts can increase their stability. The resazurin/resorufin pair has been widely used for detecting redox processes in biological settings; yet, how tight confinement affects the properties of these two dyes remains to be explored. Here, we show that a flexible Pd^II^_6_L_4_ coordination cage can efficiently encapsulate both resorufin and resazurin in the form of dimers, dramatically modulating their optical properties. Furthermore, binding within the cage significantly decreases the reduction rate of resazurin to resorufin, and the rate of the subsequent reduction of resorufin to dihydroresorufin. During our studies, we also found that upon dilution, the Pd^II^_6_L_4_ cage disassembles to afford Pd^II^_2_L_2_ species, which lacks the ability to form inclusion complexes – a process that can be reversed upon the addition of the strongly binding resorufin/resazurin guests. We expect that the herein disclosed ability of a water-soluble cage to reversibly modulate the optical and chemical properties of a molecular redox probe will expand the versatility of synthetic fluorescent probes in biologically relevant environments.

## Abstract (web summary)

Water-soluble nanocontainers that can encapsulate and modulate the properties of synthetic fluorescent probes could expand the versatility of such probes in biologically relevant environments. Here, the authors encapsulate the resazurin/resorufin redox pair within a flexible palladium-based coordination cage and find that confinement drastically alters the optical and redox properties of the two dyes.

## Introduction

The resazurin/resorufin pair has long been studied and utilized as a structurally simple system for detecting reducing environments. Upon reduction, the weakly fluorescent dye resazurin is converted into strongly emissive resorufin. This reaction is readily induced by metabolically active cells and can thus serve as a method for evaluating cell viability^1–3^. As early as 1945, Twigg proposed the use of resazurin (nowadays widely known as Alamar Blue) for monitoring bacterial and yeast contamination of milk^4^. On a larger scale, resazurin has been used to investigate metabolic activity within microbial communities in large aquatic ecosystems^5,6^ and to monitor biological activity in sewage treatment plants^7^. Resazurin has also been used to detect enzymes (such as alkaline phosphatase^8^) whose activity results in the production of reducing agents, which can then induce the formation of the fluorescent resorufin^9^. Owing to the high emission intensity of resorufin, the resazurin/resorufin pair has been employed for detecting and studying corrosion^10^ and other important processes^11–14^ on very small scales using fluorescence microscopy. Additionally, fluorescence microscopy can provide important insights into the properties of supported catalysts capable of facilitating the resazurin→resorufin reduction^15–19^. However, despite several decades of research on resazurin and resorufin, little attention has been devoted to the behavior of these two dyes within confined environments.

Placing optically active and/or redox-active species under confinement can profoundly affect their physicochemical characteristics^20–25^. For example, the oxidation potential of tetrathiafulvalene could be shifted considerably upon encircling it within macrocyclic “hosts”, such as *α*-cyclodextrin^24^ or cyclobis(paraquat-*p*-phenylene)^25^. Similarly, the optical properties of dyes can be modulated by confining them within the cavities of self-assembled cages^26–29^. Surprisingly, however, little attention has been devoted to the behavior of resorufin and resazurin in confined spaces, and no strong confinement effects for these two dyes have been reported to date. For example, the fluorescence of resorufin was not markedly affected by encapsulation within *β*-cyclodextrin^30^ or within a tetralactam macrocycle incorporating two anthracene moieties^31^. Similarly, resorufin placed within micelles^32^, microemulsion droplets^33^, and one-dimensional channels of metal–organic frameworks^34^ retained its strong emission.

**Fig. 1:**
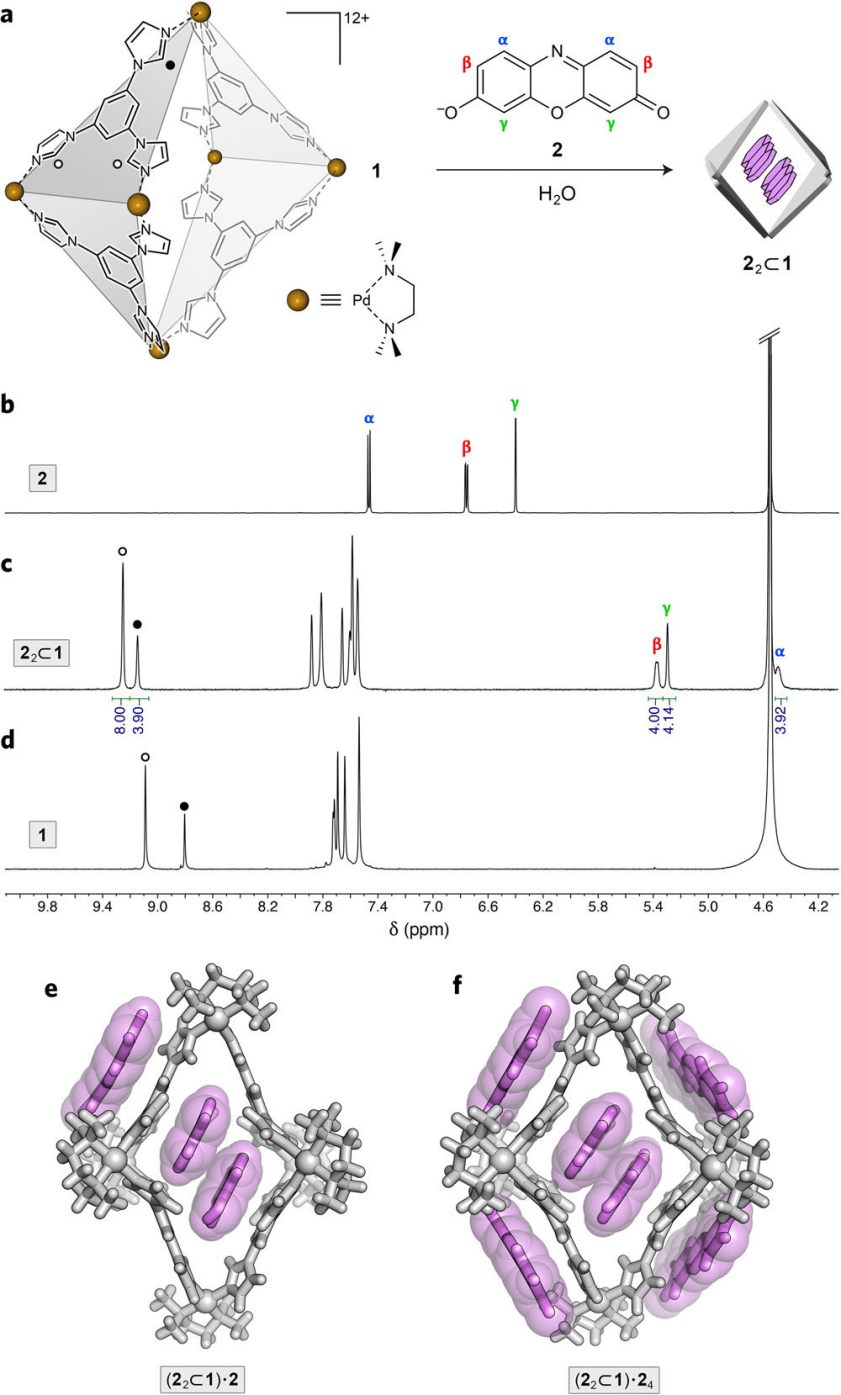
Encapsulation of resorufin within coordination cage 1. **a** Structural formulas of coordination cage **1** (counterions = 12 NO_3_^−^), resorufin **2**, and a cartoon representation of the inclusion complex **2**_2_⊂**1**. The two types of acidic imidazole protons in **1** are denoted by black and white circles. **b** ^1^H NMR spectrum of **2** (600 MHz, D_2_O, 320 K). **c** Partial ^1^H NMR spectrum of **2**_2_⊂**1** (600 MHz, D_2_O, 320 K) (for full-range ^1^H and ^13^C spectra, see Supplementary Figs. 1 and 2). **d** Partial ^1^H NMR spectrum of **1** (600 MHz, D_2_O, 320 K). **e** Crystal structure of **2**_2_⊂**1** co-crystallized with 1 equiv of unencapsulated **2**; i.e., (**2**_2_⊂**1**)⋅**2**. **f** Crystal structure of **2**_2_⊂**1** co-crystallized with 4 equiv of unencapsulated **2**; i.e., (**2**_2_⊂**1**)⋅**2**_4_. Nitrate counterions and water molecules were omitted for clarity.

Here, we investigated the host–guest complexation of resorufin and resazurin within a cationic cage based on Pd–imidazole coordination^35^ (**1** in Fig. 1a). Cage **1** is assembled from six Pd^2+^ cations (*cis*-blocked with *N*,*N*,*N*′,*N*′-tetramethylethylenediamine (TMEDA)) and four triimidazolylbenzene (TImB) ligands. Uniquely, **1** combines a well-defined cavity size with sufficient^36,37^ structural flexibility to simultaneously accommodate^38^ two small aromatic guest molecules as diverse as azobenzenes,^39,40^ BODIPY dyes^41^ and pyrene^35^. The encapsulation of these molecules is driven by the hydrophobic effect and their attractive interactions with the cage through a combination of π–π stacking and van der Waals interactions, with additional contributions from other, weaker interactions such as dispersion forces. The binding is further stabilized by attractive interactions between the two guest molecules, which reside near each other.

We hypothesized that, owing to their structural resemblance to the previously studied guests, resorufin and resazurin could similarly be encapsulated within **1**. Moreover, in contrast to the above guests (all of which are electrically neutral), both resorufin and resazurin are monoanionic, strengthening binding through Coulombic interactions with the duodecuply (i.e., +12) charged host. Indeed, we found that cage **1** can bind two molecules of both dyes, thus inducing noncovalent dimerization, which dramatically changes their optical properties, causing strong shifts in the absorption spectra and effectively turning off the emission of resorufin. Furthermore, encapsulation within the cage markedly decreases the kinetics of the reduction of resazurin to resorufin, and the further reduction of resorufin to dihydroresorufin. Our results indicate that confinement effects, ubiquitous in nature, have a profound effect on the behavior of a widely used redox probe.

## Results

### Encapsulation of resorufin within coordination cage 1

We began our experiments by studying host–guest complexation of resorufin **2** by cage **1**. The ^1^H nuclear magnetic resonance (NMR) spectrum of a 2:1 mixture of **2** and **1** recorded at room temperature featured relatively broad signals, hampering the characterization of the complex. We found, however, that gentle heating preserved the integrity of the inclusion complex while significantly sharpening the signals. Figure 1c shows a partial ^1^H NMR spectrum of the complex recorded at 320 K. The DOSY spectrum (Supplementary Fig. 3) indicates that all the protons diffuse at the same rate, suggesting that all the signals observed in the ^1^H NMR spectrum belong to a single species. The signals have been assigned after a comprehensive characterization by a suite of 2D NMR techniques (Supplementary Figs. 4–12). The signals originating from **2** (**α**, **β**, and **γ** in Fig. 1c) are upfield-shifted compared to free **2** in D_2_O (Fig. 1b), which can be attributed to the guest **2** residing within the cavity of cage **1** and experiencing the magnetic field due to the aromatic ring currents of the cage’s walls and of the coencapsulated guest. Integrating these signals with respect to the characteristic signals of **1** due to acidic imidazole protons suggests that the cage is filled with two molecules of resorufin, i.e., a homoternary inclusion complex **2**_2_⊂**1** forms. The 2:1 stoichiometry of the complex was confirmed by additional three sets of experiments, as described below.

First, we obtained single crystals of the complex by slow water evaporation from an aqueous solution of **2**_2_⊂**1**. Interestingly, the crystals featured both the inclusion complex and the free guest in an alternating fashion, i.e., (**2**_2_⊂**1**)⋅**2**, giving rise to an overall guest/host ratio of 3:1 (Fig. 1e; all X-ray structures reported herein are included as Supplementary Data 1). This finding indicates that upon crystallization, **2**_2_⊂**1** undergoes a ‘disproportionation’ reaction:

$$3n\,[{{{\bf{ 2}}}}_{2}\!\subset\! {\bf {1}}]\to 2 \,\, [({\bf {2}}_{2}\!\subset\! {\bf {1}})\!\cdot\! {\bf {2}}]_{n}\!\!\downarrow +\,n\,{\bf {1}}$$

where the symbol “↓” denotes a solid, crystalline phase, and “*n* **1**” denotes unoccupied **1** left in solution. More impressively, when the crystallization experiment was repeated using **2**_2_⊂**1** in the presence of an excess of free **1**, we once again observed the formation of [(**2**_2_⊂**1**)⋅**2**]_*n*_. This result emphasizes the unique ability of one molecule of **2**_2_⊂**1** to “extract” free guest **2** from another and to incorporate it within the crystalline lattice.

Remarkably, when crystallization was induced by diffusing acetone vapor into the aqueous solution of **2**_2_⊂**1**, we found that each **2**_2_⊂**1** moiety was surrounded by not one but four molecules of **2**, which were not shared with the neighboring cages (Fig. 1f). Therefore, the composition of the crystals obtained by antisolvent diffusion is [(**2**_2_⊂**1**)⋅**2**_4_]_*n*_ (i.e., a guest/host ratio of 6:1) and the process of crystal formation coupled with guest extraction can be written as

$$3n\,\,[{\bf {2}}_{2}\!\subset\! {\bf {1}}]\to [({{{\bf{2}}}}_{2}\!\subset\! {{{\bf{1}}}})\!\cdot\! {{{\bf{2}}}}_{4}]_{n}\!\downarrow +\,2n\,{{{\bf{1}}}}$$

The four molecules of unencapsulated **2** fit well within the shallow cavities formed by the outer surfaces of the TImB panels and the peripheral TMEDA groups (Fig. 1f). Analysis of the intermolecular packing in the crystal revealed the presence of infinite π–π stacks having the stoichiometry [**2′**⋅⋅⋅TImB⋅⋅⋅**2**⋅⋅⋅**2**⋅⋅⋅TImB⋅⋅⋅**2′**]_*n*_, where **2** denotes encapsulated resorufin, **2′** – resorufin residing between cages, and TImB – a triimidazole wall of cage **1** (for a more detailed analysis of the structure of (**2**_2_⊂**1**)⋅**2**_4_, see Supplementary Fig. 13). It is interesting to add that the initial formation of the red crystals of [(**2**_2_⊂**1**)⋅**2**]_*n*_ or [(**2**_2_⊂**1**)⋅**2**_4_]_*n*_ (during crystallization by water evaporation or acetone diffusion, respectively) was followed by the formation of colorless crystals of the empty **1**. The strong preference for crystallizing [(**2**_2_⊂**1**)⋅**2**]_*n*_ and [(**2**_2_⊂**1**)⋅**2**_4_]_*n*_ is evidenced by the formation of these two cocrystals from different mixtures of **2** and **1**, including ones containing an excess of **1**.

Despite the different amounts of free **2** in the crystals of (**2**_2_⊂**1**)⋅**2** and (**2**_2_⊂**1**)⋅**2**_4_, the **2**_2_⊂**1** units appeared nearly identical (Supplementary Fig. 14), with an antiparallel alignment of the two guests (Supplementary Fig. 15). Importantly, the structure of **2**_2_⊂**1** elucidated by X-ray crystallography is in good agreement with the solution NMR data, e.g.: (i) the signals due to **2**’s **α** protons are significantly (by ~3.0 ppm) more upfield-shifted than those of **2**_**β**_ and **2**_**γ**_ (~1.4 and ~1.1 ppm, respectively; Fig. 1b vs. 1c), which can be explained by the presence of protons **2**_**α**_ in the center of **1**’s cavity, where they are further shielded by the local magnetic field originating from the aromatic ring current of the other guest molecule; (ii) the equatorial acidic-imidazole protons of **1** (the empty circles in Fig. 1) show a weak through-space nuclear Overhauser (nOe) correlation with protons **2**_**α**_ (Supplementary Fig. 9; the shortest distance in the crystal structure between these two protons is relatively large, at 3.42 Å); and (iii) the axial acidic-imidazole protons of **1** (the black circles in Fig. 1) show a strong nOe correlation with protons **2**_**γ**_ (Supplementary Fig. 9; the shortest distance in the crystal structure between these two protons is 3.22 Å). Additional nOe correlations consistent with host–guest contacts in the crystal structure are shown in Supplementary Fig. 10.

**Fig. 2:**
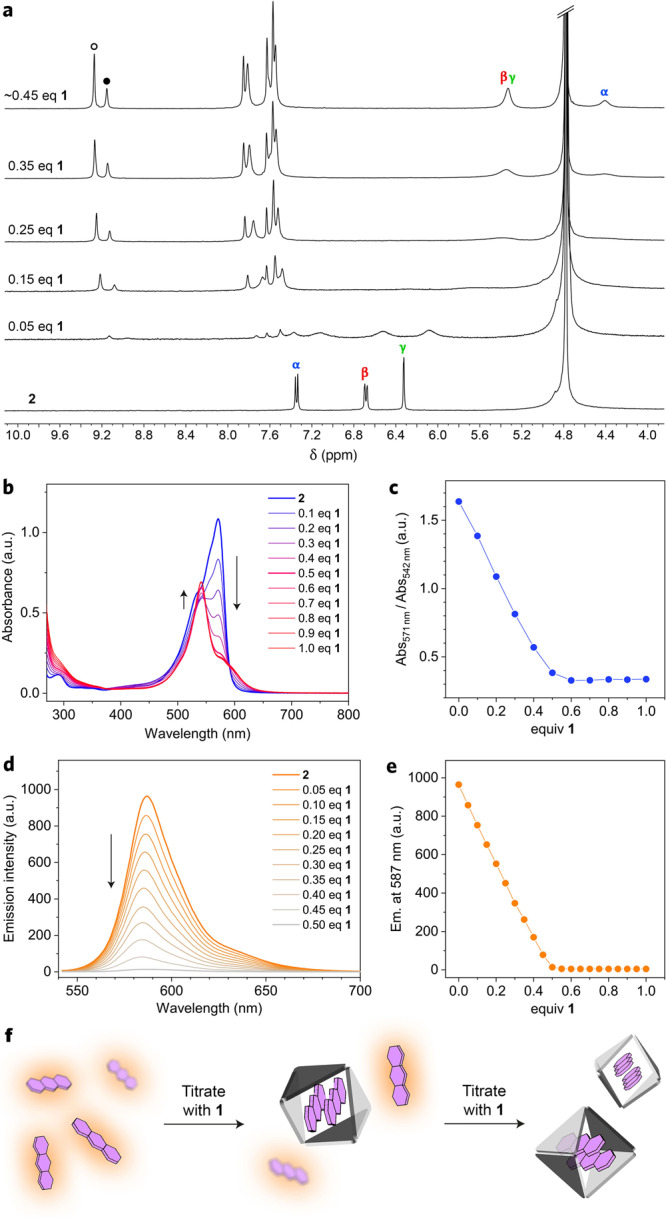
Spectroscopic characterization of the resorufin complex. **a** *Bottom to top:* ^1^H NMR spectra of **2** in the presence of increasing amounts of cage **1** (400 MHz, D_2_O, 298 K). **b** A series of UV–Vis absorption spectra accompanying the titration of **2** with cage **1**. No changes in the >400 nm region are visible beyond ~0.5 equiv of **1**, indicating a 2:1 stoichiometry of the complex. **c** Decrease in the ratio of absorbance at 571 nm (Abs_571nm_) (high for **2**; low for **2**_2_⊂**1**) to absorbance at 542 nm (Abs_542nm_) (high for **2**_2_⊂**1**; low for **2**) as a function of the amount of **1** added. **d** A series of fluorescence spectra (*λ*_exc_ = 530 nm) accompanying the titration of **2** with cage **1**. **e** Decrease of emission at 587 nm as a function of the amount of **1** added. **f** Cartoon representation of fluorescence quenching due to inclusion complex formation during the titration of **2** with cage **1**.

Second, we followed the evolution of **2**’s peaks during NMR titration with cage **1** (Fig. 2a). Upon the addition of a small amount of the cage (0.05 equiv), **2**’s peaks shifted upfield and broadened, which can be attributed to the exchange between free and encapsulated **2**, with the exchange dynamics commensurate with the NMR time scale. When the molar fraction of encapsulated **2** approached that of free **2** (0.15–0.25 equiv **1**), **2**’s signals became so broad that they were practically invisible. Further addition of the cage caused the reemergence and gradual sharpening of **2**’s peaks; no further changes to these peaks were seen beyond ~0.5 equiv of **1** (Fig. 2a), indicating a quantitative encapsulation of **2**.

Third, the 2:1 stoichiometry is also evident from the UV-Vis and fluorescence titrations of aqueous solutions of **2** with cage **1**. The blue trace in Fig. 2b shows a UV–Vis absorption spectrum of **2** solubilized in water with 1.5 equiv of a base (TMEDA), with a strong absorption centered at *λ*_max_ = 571 nm. Upon the gradual addition of **1**, the intensity of this peak decreased, concomitant with an increase of a less intense, blue-shifted band at *λ*_max_ = 542 nm. These changes are characteristic of the formation of an H-aggregate (here, H-dimer), as observed previously for a series of BODIPY dyes^41^. Notably, the 571 nm peak stopped decreasing upon the addition of 0.5 equiv of the cage, indicating that free **2** had been consumed (Fig. 2c). This result was confirmed by a fluorescence titration, in which we followed the decrease of the characteristic emission of **2** at *λ*_max_ = 587 nm (Fig. 2d). We observed a roughly linear decrease in emission intensity, which transformed the highly emissive **2** into a virtually non-emissive H-dimer once 0.5 equiv of the cage was added (Fig. 2e, f). This linear relationship suggests i) a high affinity of **2** to cage **1** and ii) a strongly cooperative binding of **2** to **1**, with the 2:1 complex-forming preferentially^42^ to a putative 1:1 complex^43^ (even in the presence of an excess of the guest; note that for a 1:1 complex, fluorescence should be preserved, at least partially^26,31,43–45^).

Fourth, we performed isothermal titration calorimetry (ITC) experiments, in which we gradually added cage **1** to a solution of **2** (solubilized in water using 1 equiv of TMEDA). By integrating the raw data, we confirmed the 2:1 binding stoichiometry (Supplementary Fig. 17) and derived the association constants, *K*_a1_ = 2.8(±1.0) × 10^5^ m^−1^ and *K*_a2_ = 4.0(±0.4) × 10^6^ m^−1^, i.e., in the range of *K*_a_ values typical of homo-^46–49^ and heteroternary^50–53^ inclusion complexes of cucurbit[8]uril and other large macrocyclic hosts. The binding cooperativity can be expressed quantitatively in terms of an interaction parameter *α* (= 4*K*_a2_/*K*_a1_ for a 2:1 stoichiometry)^54,55^ and the Hill coefficient^56^, *n*_H_ = 2/[1 + (*K*_a1_/*K*_a2_)^1/2^]. The high values of *α* ≈ 57 and *n*_H_ ≈ 1.58 confirm the strongly cooperative nature of the binding of **2** within **1** (note that for a 2:1 binding stoichiometry, *n*_H_ ranges from 0 to 2). The facile formation of a 2:1 complex can be understood by analyzing the crystal structures of **2**_2_⊂**1**, which show (Fig. 1e, f) that the size of **1**’s cavity is commensurate with two stacked guest molecules. Although DFT calculations support the existence of a 1:1 complex, a single molecule of **2** is too small to efficiently fill the cavity of **1**, even after a significant structural deformation of the cage (Supplementary Figs. 18 and 19; see also Supplementary Data 1).

We also attempted to determine the association constants by analyzing the fluorescence titration data using the online fitting tool BindFit^55,57^. Curve fitting with the 2:1 binding model afforded *K*_a1_ = 7.2(±2.4) × 10^5^ m^−1^ and *K*_a2_ = 1.5(±0.2) ×10^7^ m^−1^, i.e., values slightly (by a factor of ~2.5) higher than those obtained from ITC titrations. One reason behind this discrepancy can be the large difference in the concentration of **1** in the fluorescence vs. ITC titration experiments (~10 μm and ~2 mm, respectively), which affects the equilibrium between free **2** and its inclusion complexes **2**⊂**1** and **2**_2_⊂**1**. We also point out that the values derived from the fluorescence titration experiments bear some uncertainty as the emission of the putative **2**⊂**1** species is unknown. Nevertheless, this analysis supports the cooperative nature of the binding of **2** within **1** (*K*_a2_ ≫ *K*_a1_), suggesting that the initial binding of one molecule of **2** is readily followed by encapsulation of another one. It is interesting to point out an analogy to our previous study, in which we found that the formation of 2:1 complexes comprising cage **1** and two compact azobenzenes proceeds significantly faster than the formation of 1:1 complexes of the same cage and a bulkier azobenzene^39^.

### Reversible disassembly and guest-templated reassembly of cage 1

**Fig. 3:**
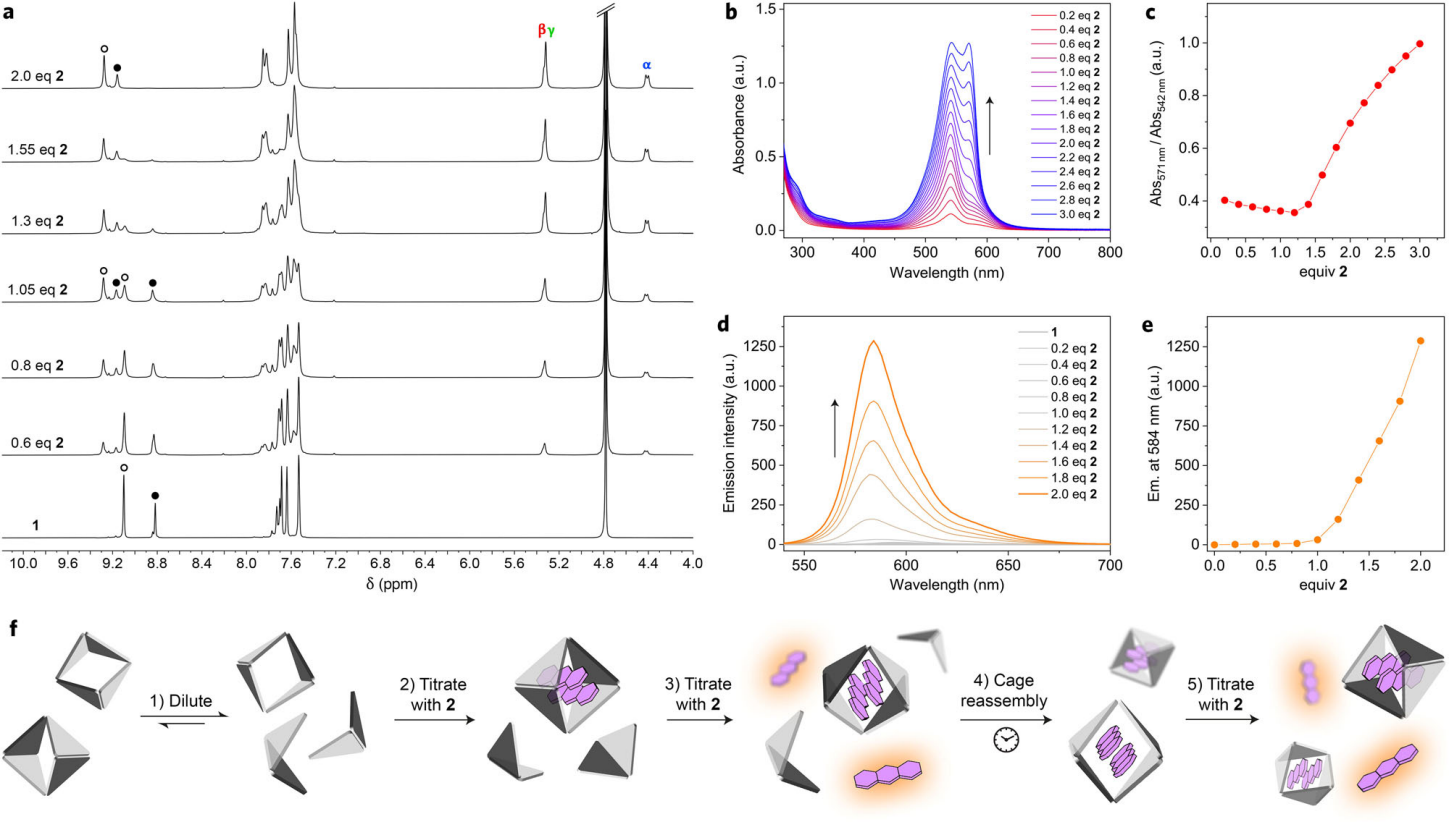
Reversible disassembly and guest-templated reassembly of cage 1. **a** *Bottom to top:* ^1^H NMR spectra of cage **1** in the presence of increasing amounts of guest **2** (deprotonated with excess TMEDA) (400 MHz, D_2_O, 298 K). **b** UV-Vis absorption spectra of cage **1** in the presence of increasing amounts of **2**. **c** Changes in the ratio of Abs_571nm_ (high for **2**; low for **2**_2_⊂**1**) to Abs_542nm_ (high for **2**_2_⊂**1**; low for **2**) as a function of the amount of **2** added. The sharp increase of Abs_571nm_/Abs_542nm_ at ~1.4 equiv **2** indicates that **2** added at that point is not complexed. **d** A series of fluorescence spectra (*λ*_exc_ = 530 nm) accompanying the titration of cage **1** with **2**. **e** Following the emission at 584 nm (due to uncomplexed **2**) as a function of the amount of **2** added. The sharp increase of emission at ~1.2 equiv **2** indicates that the added **2** is not complexed. **f** Cartoon representation of fluorescence fluctuations during the titration of a dilute solution of cage **1** with **2**.

Next, we studied the assembly of the **2**_2_⊂**1** complex by the reverse-titration method, whereby a solution of cage **1** (*c*_**1**_ ≈ 10 mm) is titrated with guest **2**. Figure 3 shows a series of ^1^H NMR spectra of **1** in the presence of increasing amounts of **2**. Notably, since **1** is present in excess with respect to **2** at all stages of the titration, there are no free guests in the system and the signals in the spectra remain sharp throughout the experiment (in contrast to the series of NMR spectra in Fig. 2a). After ~1 equiv of the guest had been added, the spectrum featured sharp peaks and resembled the spectrum of **2**_2_⊂**1** superimposed on that of free **1**. This result indicates a relatively slow (on the NMR timescale) shuttling of **2** between the cages (in contrast to the fast exchange between free and bound **2**; Fig. 2a). The spectrum at 2.0 equiv of **2** is nearly identical to the final spectrum of the experiment in Fig. 2a (i.e., the **2**_2_⊂**1** complex).

The formation of **2**_2_⊂**1** was also followed by UV-Vis and fluorescence titrations (at concentrations typical for these techniques; *c*_**1**_ ≈ 10 μm). Figure 3b shows a series of UV-Vis spectra of **1** in the presence of increasing amounts of **2**. Since all of the titrant added at the early stages of the titration is encapsulated, the ratio of Abs_571nm_ (high for **2**) to Abs_542nm_ (high for **2**_2_⊂**1**) is roughly constant, and it is expected to increase only beyond ~2 equiv of the guest added. Surprisingly, however, we repeatedly observed that the ratio Abs_571nm_/Abs_542nm_ increased after only 1–1.5 equiv of **2** were added (Fig. 3c). A similar conclusion was reached from the fluorescence titration experiments. Here, the strong emission characteristic of free **2** is expected to arise once all the cages have been doubly filled (i.e., at >2 equiv of **2** added), however, it was observed at an earlier point (Fig. 3e).

**Fig. 4:**
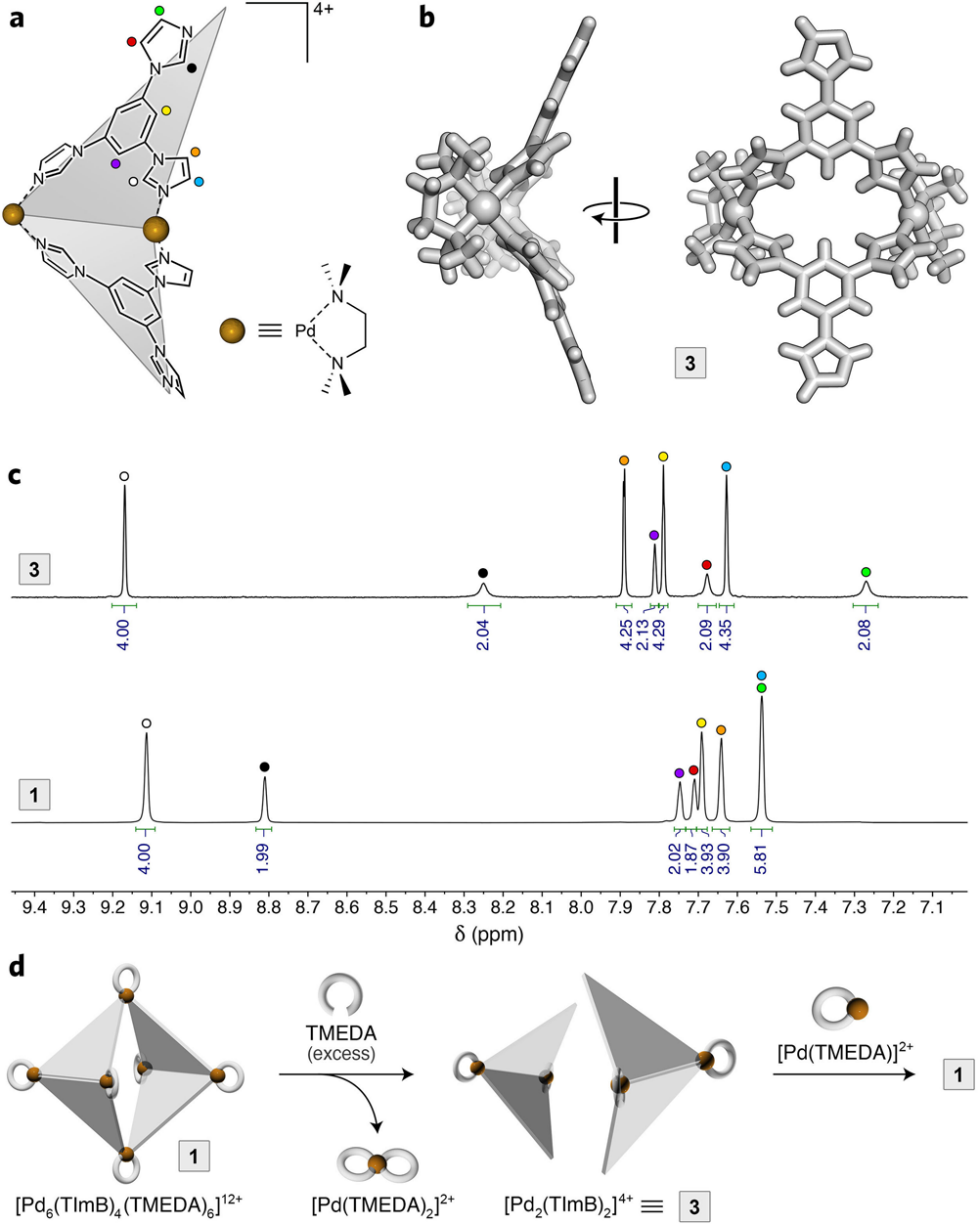
Characterization of “half-cage” 3. **a** Structural formula of **3**. **b** Crystal structure of **3**. Nitrate counterions and water molecules were omitted for clarity. **c** *Top:* Partial ^1^H NMR spectrum of **3** (600 MHz, D_2_O, 300 K) (for full-range ^1^H and ^13^C spectra, see Supplementary Figs. 20–22). *Bottom:* Partial ^1^H NMR spectrum of **1** (600 MHz, D_2_O, 300 K). **d** Cartoon representation of the extraction of axial Pd^2+^ centers from cage **1** using TMEDA, followed by reassembly of cage **1** from **3** using [Pd(TMEDA)]^2+^.

To explain the differences between the results of NMR vs. UV–Vis/fluorescence titrations, we note that the former experiments are carried out at a cage concentration three orders of magnitude higher than the latter. We also note that cage **1** is composed of multiple subcomponents held together by noncovalent interactions (**1** ⇌ 6 Pd^2+^ + 4 TImB + 6 TMEDA); thus, diluting the solution shifts the complexation equilibria towards the free metal ions and ligands. Indeed, we observed that diluting a typical NMR sample of **1** (*c* ≈ 8 mm) by a factor of 160 (down to ~0.05 mm) not only decreases the signal intensity, but it also greatly increases the complexity of the spectra, with many new signals appearing (Supplementary Fig. 27). We identified many of these signals as originating from “half-cage” [Pd_2_(TMEDA)_2_(TImB)_2_]^4+^, composed of two TImB panels “stapled” by two Pd^2+^ ions (**3** in Fig. 4; see below for characterization). This species, which arises at low concentrations (Fig. 3f, step 1), is incapable of forming inclusion complexes, which explains why titrations are complete at lower-than-expected amounts of guest **2**.

At a low concentration, the equilibrium is shifted towards disassembled **1**:

$${{{\bf{1}}}}\;\rightleftharpoons\; 2\;{{{\bf{3}}}}+2\,{[{{{{{\rm{Pd}}}}}}({{{{{\rm{TMEDA}}}}}})]}^{2+}$$

(where the two empty coordination sites on Pd^2+^ in [Pd(TMEDA)]^2+^ are occupied by water molecules; counterions = NO_3_^−^). Interestingly, however, the addition of **2** during the titration experiments can induce the reassembly of **1**,

$$2\;{{{\bf{3}}}}+2\;[{{{{{\rm{Pd}}}}}}({{{{{\rm{TMEDA}}}}}})]^{2+}+2\;{\bf {2}}\to {\bf {2}}_{2}\!\subset\! {\bf {1}}$$

We reached this conclusion based on the following experiments. First, we titrated a dilute solution of **1** with **2**, while monitoring the fluorescence. During the initial stages of the titration, the fluorescence remained weak due to the rapid encapsulation of the fluorescent titrant (step 2 in Fig. 3f). However, the solution became fluorescent at a substoichiometric amount of **2** (step 3). The titration was halted and the solution was allowed to equilibrate; interestingly, the emission slowly (within a few hours) disappeared, indicating that **2**-templated reassembly of cage **1** (specifically, the non-fluorescent complex **2**_2_⊂**1**; step 4) took place. The oscillations in fluorescence could be repeated for multiple cycles (we followed this process during NMR titration), but only when <2 equiv of the guest were present; at >2 equiv of **2**, all the cages were reassembled and the fluorescence only increased (Fig. 3f, step 5). In a similar experiment, we repeated the UV-Vis titration of dilute **1** with **2** (as in Fig. 3b) until 2.0 equiv of **2** were added. At that point, we allowed the solution to equilibrate overnight and remeasured the spectrum, obtaining a spectrum more reminiscent of the **2**_2_⊂**1** complex (Supplementary Fig. 16), which confirms that the presence of the guest facilitates the assembly of the cage.

To confirm the identity of **3**, we synthesized it in a pure form by reacting TImB with 1.0 equiv of [Pd(TMEDA)]^2+^(NO_3_^−^)_2_ (as opposed to 1.5 equiv, which affords cage **1**) (see Supplementary Methods, Section 3). Mass spectrometry analysis with electrospray ionization and negative ion detection afforded the expected molecular mass (Supplementary Figs. 29 and 30). Single crystals of **3** were prepared by slow water evaporation from the aqueous solution; Fig. 4b shows the X-ray crystal structure of **3**, which is highly reminiscent of the structure of the two adjacent halves of **1** (see, e.g., Fig. 1e). The ^1^H NMR spectrum of **3** (Fig. 4c, top) resembles that of **1** (Fig. 4c, bottom), except for the signals originating from the axial imidazoles, which are no longer coordinated to Pd (red, green, and black in Fig. 4). The structure of **3** was further confirmed by 2D NMR techniques (Supplementary Figs. 23–26). Interestingly, Fujita and co-workers proposed the existence of dinuclear macrocycles similar to **3** as putative intermediates during the self-assembly of cages resembling **1**^58^.

To further confirm the relationship between **1** and **3**, we performed a controlled disassembly of **1** into **3** and the reassembly of **1** from **3**, as schematically shown in Fig. 4d. To this end, we first treated **1** with a large excess (~350 equiv) of TMEDA, which preferentially sequestered the axial Pd^2+^ centers, disassembling the cage into **3**

$${{{\bf{1}}}}+{2}\;{{{{{\rm{TMEDA}}}}}}\to {2}\,\,{ {{\bf{3}}}}+{2}\;{[{{{{{\rm{Pd}}}}}}{({{{{{\rm{TMEDA}}}}}})}_{2}]}^{2+}$$

(over time, **3** is further decomposed as a result of the equatorial Pd^2+^ reacting with TMEDA and/or OH^−^). The enhanced reactivity at the axial locations is noteworthy, given that both the axial and the equatorial Pd^2+^ have the same immediate coordination environment (one TMEDA and two TImB ligands). We note, however, that whereas the cleavage of an imidazolyl–Pd_axial_ bond is akin to opening a macrocyclic ring, the dissociation of an imidazolyl–Pd_equatorial_ bond retains the overall architecture of the cage, resembling the chelate effect on a larger scale.

In the second experiment (Fig. 4d, right), we treated **3** with 1 equiv of [Pd(TMEDA)]^2+^ and observed an efficient formation of **1** (Supplementary Fig. 28). This strategy to assemble cage **1** is akin to the stepwise assembly of discrete metal–organic architectures reported before^59^.

### Encapsulation of resazurin within coordination cage 1

**Fig. 5:**
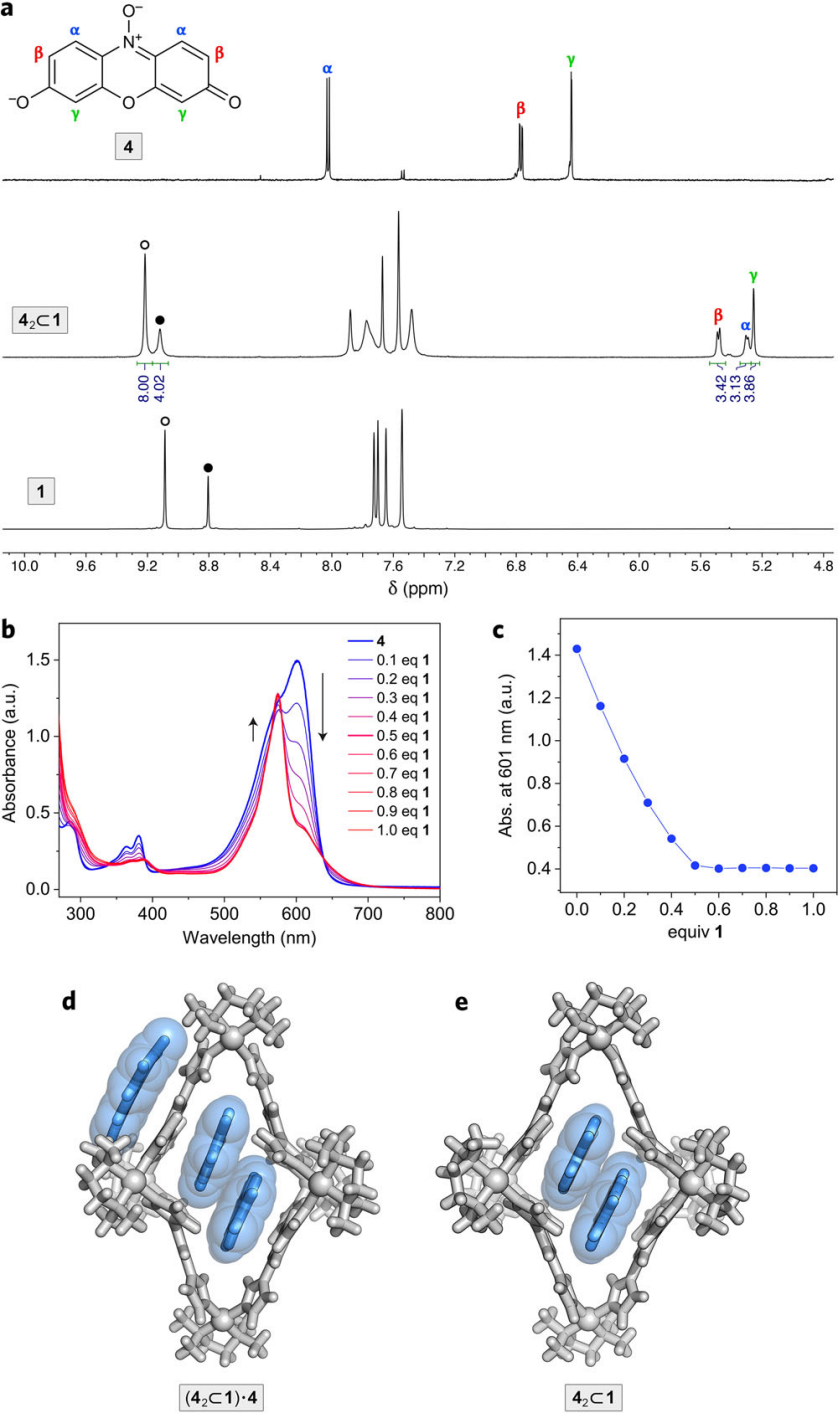
Encapsulation of resazurin within coordination cage 1. **a** *Top:* Structural formula and ^1^H NMR spectrum of resazurin **4** (600 MHz, D_2_O, 330 K). *Center:* Partial ^1^H NMR spectrum of **4**_2_⊂**1** (600 MHz, D_2_O, 330 K) (for full-range ^1^H and ^13^C spectra, see Supplementary Figs. 31 and 32). *Bottom:* Partial ^1^H NMR spectrum of **1** (600 MHz, D_2_O, 330 K). **b** A series of UV–Vis absorption spectra accompanying the titration of **4** with a concentrated solution of cage **1**. No changes in the >400 nm region are visible beyond 0.5 equiv of **1**, indicating a 2:1 stoichiometry of the complex. **c** Decrease of absorbance at 601 nm (high for **4**; low for **4**_2_⊂**1**) as a function of the amount of **1** added. **d** Crystal structure of **4**_2_⊂**1** co-crystallized with 1 equiv of unencapsulated **4**; i.e., (**4**_2_⊂**1**)⋅**4**. **e** Crystal structure of **4**_2_⊂**1**. Nitrate counterions and water molecules were omitted for clarity.

Prior to investigating the reactivity of encapsulated **2**, we studied the suitability of cage **1** for encapsulating the *N*-oxide counterpart of **2**, namely, resazurin **4** (Fig. 5). We found that the complexation characteristics of **4** were analogous to those of **2** in that **1** encapsulated two molecules of **4**, and all three signals in the ^1^H NMR spectrum of **4** became upfield-shifted upon encapsulation (with **4**_**α**_, located in the center of **1**’s cavity, exhibiting the largest shift of ~2.7 ppm; Fig. 5a). Notably, these changes are only observed upon encapsulation within the cage; both **2** and **4** are highly soluble in water in their anionic forms, with no signs of aggregation observed even at the high-millimolar concentrations of the unencapsulated dyes. Further NMR characterization of **4**_2_⊂**1** is provided in Supplementary Figs. 33–43.

Analogously to (**2**_2_⊂**1**)⋅**2**, we observed the tendency of **4**_2_⊂**1** to form crystals with a stoichiometry of (**4**_2_⊂**1**)⋅**4** (Fig. 5d); these crystals were obtained by the diffusion of acetone layered on top of an aqueous solution of **4**_2_⊂**1**. However, when we repeated the experiment by diffusing acetone vapors into the same solution of **4**_2_⊂**1**, we instead observed the crystallization of **4**_2_⊂**1** without any extra uncomplexed **4** (Fig. 5e). The structures of the inclusion complex in both (**4**_2_⊂**1**)⋅**4** and **4**_2_⊂**1** were nearly identical (Supplementary Fig. 44), with an antiparallel alignment of the two guests (Supplementary Fig. 45). It is worth pointing out that the smaller amount of unencapsulated **4** within the crystals of **4**_2_⊂**1** (zero and one extra molecule per inclusion complex in the two crystals), compared with **2**_2_⊂**1** (one and four molecules, respectively), correlates with the higher aqueous solubility of **4** compared with that of **2**.

Compared with **2**, resazurin **4** has an extra oxygen atom located at the center of the molecule; to accommodate the extra steric demand, the host undergoes a more significant structural distortion, developing two “compartments”, each accommodating one molecule of the guest (see Fig. 5d, e). We have previously observed a similar deformation of cage **1**, accompanying the encapsulation of two molecules of tetra-*o*-fluoroazobenzene^39^.

The association constants of **4** to **1** were determined using ITC (analogously to the binding of **2** to **1**; see above). We found that the binding strength of both guests within **1** is comparable, in agreement with their similar molecular shapes and sizes. The binding of the first molecule of **4** (*K*_a1_ = 1.0(±0.4) × 10^5^ m^−1^) is slightly weaker than for **2**, which could be due to the higher solubility of free **4**. In contrast, the binding of the second guest (*K*_a2_ = 1.2(±0.2) × 10^7^ m^−1^) was stronger than for **2**, which can be explained by more efficient filling of the cage’s cavity by the slightly larger **4** (compare Fig. 5d, e with Fig. 1e, f). This combination of a lower *K*_a1_ and a higher *K*_a2_ give rise to **4**’s much higher binding cooperativity of *α* ≈ 480 and *n*_H_ ≈ 1.83 (compared with *α* ≈ 57 and *n*_H_ ≈ 1.58 for **2**), whereas the overall stabilities of both 2:1 complexes are nearly the same (cumulative binding constants, *K*_a1_*K*_a2_ = 1.1 × 10^12^ m^−2^ for **2**_2_⊂**1** and 1.2 × 10^12^ m^−2^ for **4**_2_⊂**1**). The association constants and thermodynamic parameters for the binding of cage **1** to both guests are listed in Table 1. Further characterization of **4**_2_⊂**1** is provided in Supplementary Figs. 46–50.

**Table 1:** Binding constants and thermodynamic parameters for the binding of cage 1 to guests 2 and 4, derived from ITC titrations.

Reaction	*K*_a_ (m^−1^)	Δ*H* (kJ mol^−1^)	*−T*Δ*S* (kJ mol^−1^)	Δ*G* (kJ mol^−1^)
**2** + **1** → **2**⊂**1**	2.8 (±1.0) × 10^5^	−17.5 (±3.9)	−13.9 (±4.5)	−31.4 (±0.9)
**2**⊂**1** + **2** → **2**_2_⊂**1**	4.0 (±0.4) × 10^6^	−33.7 (±1.0)	−4.3 (±1.3)	−38.0 (±0.3)
**4** + **1** → **4**⊂**1**	1.0 (±0.4) × 10^5^	−13.6 (±0.8)	−15.2 (±1.8)	−28.8 (±1.0)
**4**⊂**1** + **4** → **4**_2_⊂**1**	1.2 (±0.2) × 10^7^	−33.3 (±0.9)	−7.5 (±1.3)	−40.8 (±0.4)

### Photoreduction of resazurin and resorufin in the absence and presence of cage 1

**Fig. 6:**
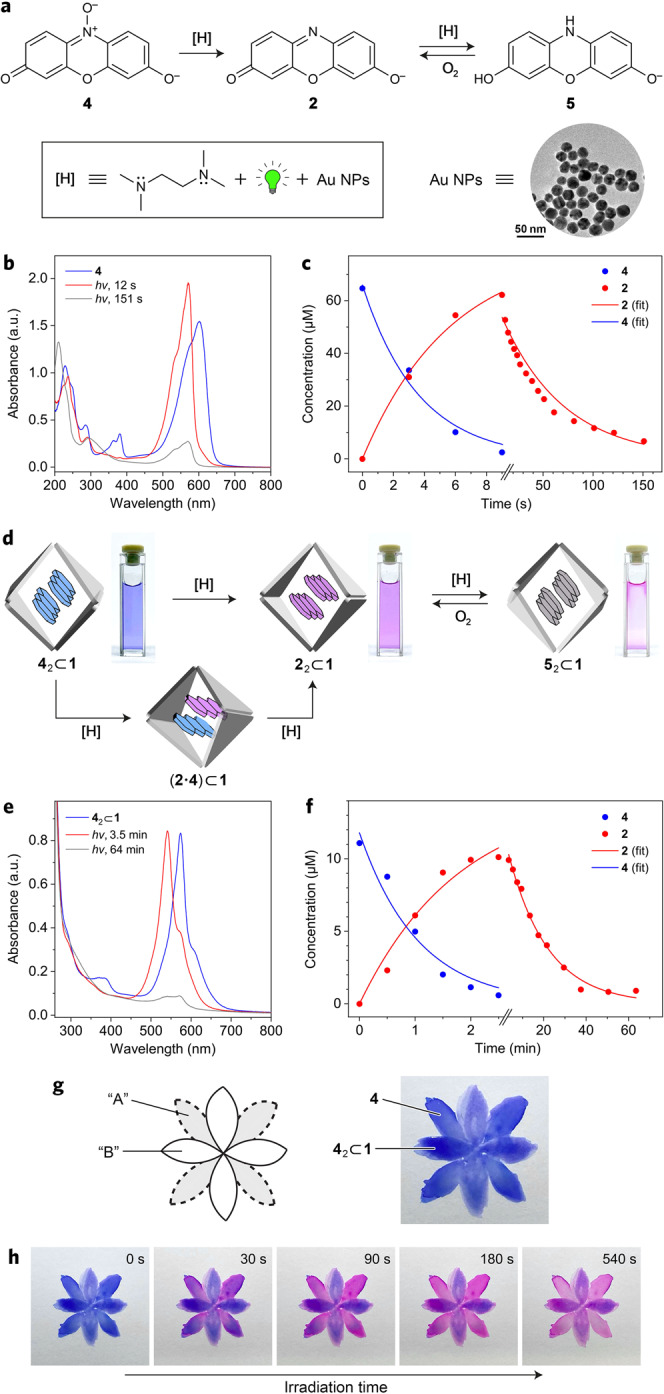
Modulating the reactivity of resazurin and resorufin by encapsulation within cage 1. **a** Reduction of resazurin **4** to resorufin **2** and the subsequent reduction of **2** to dihydroresorufin **5**. Inset: A transmission electron micrograph of 21 nm citrate-capped Au NPs. **b** Representative UV–Vis absorption spectra of **4** before (blue) and after exposure to green light in the presence of TMEDA and Au NPs for 12 s (red; mostly **2**) and for 151 s (gray; mostly **5**). Note that due to the rapid oxidation of **5** back to **2**, it was very challenging to eliminate the residual absorption at ~550 nm due to **2**. Note also that Au NPs are used in such a small amount that their contribution to UV–Vis spectra is not visible. **c** Concentrations of **2** and **4** (extracted from UV–Vis spectra) at various reaction times (markers) and fits to first-order kinetics (lines). **d** Cartoon representation of the sequential reduction of **4**_2_⊂**1** into **2**_2_⊂**1** and then into **5**_2_⊂**1** and the corresponding photographs of the reaction mixtures. The pink color at the water/air interface for **5**_2_⊂**1** is due to the rapid oxidation to **4**_2_⊂**1**. **e** Representative UV–Vis absorption spectra of **4**_2_⊂**1** before (blue) and after exposure to green light in the presence of TMEDA and Au NPs for 3.5 min (red; mostly **2**_2_⊂**1**) and for 64 min (gray; mostly **5**_2_⊂**1**). **f** Concentrations of encapsulated **2** and **4** (extracted from UV–Vis spectra) at various times of the reaction (markers) and fits to first-order kinetics (lines). **g** Photograph of a flower painted using a combination of **4** and **4**_2_⊂**1** dyes (4.5 and 2.25 mm, respectively). The paper was soaked with TMEDA (18 mm) and Au NPs (0.11 nm). **h** Effect of green light irradiation on the flower’s color.

Having characterized both inclusion complexes, we proceeded to study the reduction of **4**_2_⊂**1** into **2**_2_⊂**1**. A widely used system for converting **4** into **2** is based on hydroxylamine as the reducing agent and gold nanoparticles (NPs) under green light as the photocatalyst^60–66^. In this system, green light excites the localized surface plasmon resonance band of Au NPs^67,68^, facilitating the electron transfer from NH_2_OH to **4**. The reduction of **4** into **2** has also been achieved using triethylamine under otherwise the same conditions (Au NPs, green light)—a finding^63^ that inspired us to consider the use of TMEDA as the reductant. Here, we note that each molecule of cage **1** contains six molecules of TMEDA—also a tertiary amine—as the capping ligands on the Pd^2+^ nodes; we therefore hypothesized that an elegant (i.e., limiting the number of components in the system) way to accomplish the reduction of **4** would be to use an extra amount of TMEDA (Fig. 6a).

To this end, we first studied the reduction of **4** in the absence of the cage (unless stated otherwise, all reactions were carried out in deoxygenated solutions). When a dilute, blue solution of **4** containing 4 equiv of TMEDA and 4.3 × 10^−7^ equiv of citrate-capped 21 nm Au NPs was exposed to green LED irradiation for only 12 s, it turned pink, and a UV–Vis absorption spectrum identical to that of **2** was obtained (Fig. 6b), indicating that the conversion **4** → **2** took place quantitatively. We confirmed that the same reaction occurred using other tertiary amines—triethylamine and *N*,*N*-diisopropylethylamine (DIPEA)—although it was somewhat slower, most likely because of an increased steric hindrance (Supplementary Fig. 54).

Owing to the light-sensitive properties of **4** and the complexity of the system, it was necessary to conduct several control experiments. First, we treated **4** with TMEDA (4 equiv) in the dark and in the absence of NPs and found that only ~10% of **4** was reduced to **2** overnight (Supplementary Fig. 55a; compare with a quantitative reduction within 12 s for the catalyzed reaction). Second, an aqueous solution of **4** was irradiated with green light in the absence of Au NPs and TMEDA. Here, we observed slow photobleaching of **4**, with the absorbance of the main band at 601 nm decreasing by ~10% (without any appreciable formation of **2**) over the initial 9 min of irradiation (Supplementary Fig. 55b). A similar result was obtained when photoirradiation was repeated in the presence of TMEDA (Supplementary Fig. 55c). Finally, **4** was exposed to light in the absence of TMEDA but with Au NPs; we observed a ~15% decrease in intensity during the initial 10 min of irradiation (Supplementary Fig. 55d). These control experiments show that irradiation with green light induces slow photobleaching of **4**, irrespective of the presence of either TMEDA or Au NPs; nevertheless, this decomposition reaction is much slower than the reduction to **2** in the presence of both TMEDA and NPs (Fig. 6b). Thus, we can conclude that all three components of the system—TMEDA, Au NPs, and green light—have to be present for a facile **4** → **2** reduction to take place.

The experimentally observed absorption changes were converted into concentration vs. time profiles using the Lambert–Beer law (based on the spectra of pure **2** and **4**). The left-hand side of Fig. 6c shows representative profiles for the decay of **4** and the production of **2**; when plotted on a log scale, the concentration of **4** decreased linearly, indicating a pseudo-first-order reaction, with a rate constant *k*_1_ = 17.5 ± 2.6 min^−1^. Interestingly, once the absorption due to **2** stabilized at ~10 s, it started to decay when photoirradiation was continued significantly longer (Fig. 6b, c), eventually turning the solution colorless. Similar to the first reduction step, the decay of **2** followed first-order kinetics, with *k*_2_ = 0.88 ± 0.11 min^−1^. The second reduction was reversible—after having been shaken in air, the pink solution and UV–Vis spectrum of **2** rapidly recovered. These results are indicative of the reversible reduction^4,69–71^ of **2** into dihydroresorufin **5**, and they demonstrate that the TMEDA/Au NPs/light system can reduce **4** all the way to **5**. After the irreversible reduction of **4** into **2**, the conversion between **2** and **5** could be repeated for many cycles simply by alternately exposing the system to green light and oxygen (see Supplementary Figs. 52 and 53). The two-step reduction of **4** into **2** and then into **5** could also be followed by monitoring the fluorescence of the solution (low for **4**; high for **2**; no emission for **5**); representative examples are shown in Supplementary Figs. 52 and 53.

Finally, we investigated the reduction of encapsulated **4** (Fig. 6d–f). First, it was important to confirm that cage **1** remains stable under the conditions used for reducing **4** into **2**. To this end, free **1** was subjected to green light irradiation in the presence of 8 equiv of TMEDA and the same amount of Au NPs as in the above experiments; no significant changes in the UV–Vis spectra were observed (Supplementary Fig. 56), demonstrating that reduction of Pd^2+^ into Pd^0^ does not take place. However, when **4**_2_⊂**1** was subjected to the same reducing conditions, the blue color characteristic of **4**_2_⊂**1** turned pink/purple, indicating the transformation of **4**_2_⊂**1** into **2**_2_⊂**1**. Similar to the reduction of free **4**, this reaction was found to follow first-order kinetics, albeit with a significantly lower rate constant of *k*_1_′ = 0.70 ± 0.18 min^−^^1^ (i.e., ~25 times lower than for free **4** → **2**). Upon prolonged irradiation, further reduction to **5**_2_⊂**1** was observed, with the solution turning colorless. The first-order reaction rate constant for the second step (*k*_2_′ = 0.057 ± 0.013 min^−1^) was once again much slower (by a factor of ~15.5) compared with free **2** → **5**. Although **5**_2_⊂**1** was too challenging to characterize, owing to its rapid oxidation back to **4**_2_⊂**1**, we demonstrated the ability of **1** to form a 2:1 inclusion complex with a more stable analog of **5**, namely, phenoxazine **6**, which lacks the OH substituents and thus is less prone to oxidation (Supplementary Figs. 58–66).

Since the reduction of **4** to **2** and of **2** to **5** most likely occurs on the surface of an NP, it is reasonable to assume that the reaction involving guests bound inside the cage would be very slow (if it occurs at all). Instead, the reaction most likely occurs on a small fraction of free **4**/**2** in equilibrium with the respective inclusion complex, with its rate determined by the kinetics of the unbinding of **4** and **2** from the cage. Let us consider two extreme cases: first, if the binding/unbinding events were infinitesimally slow, the reduction deceleration factor would correspond to the ratio of the equilibrium concentrations of encapsulated/free species, i.e., ~10^4^. If, on the other hand, the kinetics of encapsulation/release were extremely fast (relative to the rate of reduction), there would be no difference between the reduction rate in the presence or the absence of the cage. Our finding that the reactions slowed down by factors of ~25 and ~15.5 indicate fast binding/unbinding of guests within/from cage **1** (see also the guest exchange experiment described in the next paragraph) – in agreement with the open structure (i.e., large windows) of the cage. To confirm this reasoning, we repeated the reaction **4**_2_⊂**1** → **2**_2_⊂**1** (i.e., **4** + 0.5 equiv of cage **1**) in the presence of increasing amounts of a free cage (an additional 1, 2, and 5 equiv). The extra cage decreases the availability of free **4** and it is expected to further decelerate the reaction. Indeed, we found that the reaction rate was inversely proportional to the amount of **1** in the system (Supplementary Fig. 57). We note that if the reaction occurred on the encapsulated **4**, the rate would be independent of the amount of free **1** in the system.

We note that since each cage molecule is filled with two guests, the reduction of **4**_2_⊂**1** into **2**_2_⊂**1** most likely proceeds via a ternary complex (**2**⋅**4**)⊂**1** (Fig. 6d, bottom). To demonstrate the existence of this heterodimer, we first recorded a UV–Vis spectrum of a 1:1 mixture of **2**_2_⊂**1** and **4**_2_⊂**1** (see Supplementary Note). If the stability of the two homodimers was significantly higher than that of (**2**⋅**4**)⊂**1**, or if the kinetics of guest exchange were slow, the resulting spectrum would correspond to a superposition of the two homodimer spectra; however, we observed the emergence of a distinct absorption band centered at 556 nm, which can be attributed to the heterodimer (Supplementary Fig. 67). The band retained shoulders on both sides, indicating that (**2**⋅**4**)⊂**1** exists in equilibrium with the two homodimers. Interestingly, the modified spectrum was observed instantly after mixing **2**_2_⊂**1** and **4**_2_⊂**1**, confirming the rapid kinetics of guest exchange. Second, we found that the ^1^H NMR spectrum obtained after mixing **2**_2_⊂**1** and **4**_2_⊂**1** was more complex than the sum of the spectra of the two homodimers; in addition to two sets of three signals (due to **2** co-encapsulated with **2**, and **4** co-encapsulated with **4**), we observed a new set of six signals, which can be attributed to the guests within (**2**⋅**4**)⊂**1** (Supplementary Fig. 69; see also Supplementary Fig. 70). Importantly, the intensities of all 12 signals were similar, indicating that mixing **2**_2_⊂**1** and **4**_2_⊂**1** results in a roughly statistical mixture of the two homodimers and the heterodimer – as might be expected from the similar binding constants of **2** and **4** within **1**. The DOSY spectrum revealed that all three inclusion complexes—**2**_2_⊂**1**, **4**_2_⊂**1**, and (**2**⋅**4**)⊂**1**—diffused at approximately the same rate, as expected (Supplementary Fig. 71). Last, we found that the emission intensity of the sample obtained upon mixing **2**_2_⊂**1** and **4**_2_⊂**1** was slightly higher than that of both homodimers (Supplementary Fig. 68). This observation not only confirms the formation of (**2**⋅**4**)⊂**1**; it also indicates that the proximity of **4** quenches the emission of **2** in a way similar to that observed within the **2**_2_⊂**1** homodimer.

Finally, we present a visually appealing demonstration of how cage **1** modulates the reduction kinetics of **4** to **2** and then **2** to **5**. To this end, we painted a blue, eight-petal flower on vellum paper soaked with TMEDA and a minute amount of Au NPs (far below the detection limit of the human eye). Although the flower appeared uniformly blue, its petals were painted using free and encapsulated **4**, in an alternating fashion (regions “A” and “B” in Fig. 6g, respectively). However, following green light irradiation for 90 s, the contrast between regions “A” and “B” became apparent (Fig. 6h) since a substantial fraction of free **4** underwent reduction to the pink **2**—a reaction that was suppressed in the presence of the cage in regions “B”, where **4**_2_⊂**1** remained largely unaffected. After an additional 450 s of light exposure, the pink color in regions “A” faded as **2** underwent further reduction to **5** (which was difficult to capture because of the rapid back-oxidation of **5** to **2**). During the same time, petals “B” turned purple owing to the partial reduction of **4**_2_⊂**1** to **2**_2_⊂**1** (Fig. 6h). Note that residual and/or atmospheric water is important at both stages of the reduction, which entail the loss of an oxygen atom and the addition of hydrogen atoms, respectively. No color changes were observed in the absence of either TMEDA or Au NPs.

## Conclusions

In sum, we investigated the supramolecular complexation of the resazurin and resorufin dyes by a Pd^2+^/triimidazole-based metal–organic cage **1**. We found that the cage interacts strongly with both chromophores, forming complexes with a 1:2 stoichiometry; hence it enables the formation of minimal aggregates (i.e., dimers) of both dyes, dramatically changing their optical properties. In particular, we found that the strong emission of free resorufin is effectively quenched. Although the cage was previously shown to form 1:2 complexes with various flat, aromatic molecules^35,39–41^, only the use of water-soluble guests reported here allowed us to explore the cooperative nature of binding within **1**, which we confirmed independently by ITC and fluorescence titration experiments. We also observed contrasting behaviors in host-to-guest vs. guest-to-host titrations and attributed them to the disassembly of the cage at concentrations typical for UV–Vis and fluorescence spectroscopy. Remarkably, at these low concentrations, nearly half of the cage undergoes disassembly into species incapable of forming inclusion complexes. However, the addition of strongly binding guests shifted the equilibrium back to the assembled cage, demonstrating the guests’ ability to template the formation of the cage^72^. These results can be described by the effect of the template on lowering the cage’s critical self-assembly concentration^73,74^. We found that the encapsulation of resazurin within the cage decreased the rate of reduction to resorufin by a factor of ~25 and that the subsequent reduction to dihydroresorufin proceeded ~15 times slower. These results indicate that the cage can protect the guest against chemical transformation, analogously to the previously reported stabilization of radical initiators^75^, photochromic compounds^76^, and other sensitive species^77^. The limited degree of protection is in agreement with the open structure of cage **1**, which results in the fast kinetics of guest exchange. Overall, our results have the potential to expand the versatility of molecular probes for following redox processes in biologically relevant environments through the use of supramolecular hosts.

## Methods

### Materials

All commercial chemicals were used as received unless stated otherwise. Cage **1** was synthesized according to ref. ^76^. Briefly, TMEDA (50 mg, 0.43 mmol) was dissolved in 6 mL of DMSO and Pd(NO_3_)_2_·*x*H_2_O (100 mg, 0.43 mmol) was added. The mixture was heated at 80 °C until Pd(NO_3_)_2_ dissolved (~10 min). Next, TImB (74 mg, 0.27 mmol) was added, and the mixture was heated for an additional 2 h. The solution was then filtered through cotton wool to remove a dark flocculate, and 25 mL of ethyl acetate was added to the filtrate. The resulting precipitate was centrifuged, washed 4–5 times with anhydrous acetone, and dried under vacuum, resulting in a white powder.

### Nuclear magnetic resonance (NMR) spectroscopy

NMR spectra were recorded in D_2_O or DMSO-*d*_6_. ^1^H NMR spectra were recorded on a Bruker Avance III 400 MHz spectrometer, a Bruker Avance III HD 500 MHz spectrometer, and a Bruker Avance III 600 MHz spectrometer. ^13^C NMR spectra and all 2D NMR spectra (^1^H DOSY, ^1^H–^1^H COSY, ^1^H–^1^H NOESY, ^1^H–^1^H TOCSY, ^1^H–^1^H ROESY, and ^1^H–^13^C HSQC) were recorded on a Bruker Avance III 600 MHz spectrometer. Chemical shifts (*δ*) are expressed in parts-per-million (ppm) and reported relative to the resonance of residual solvent (4.79 ppm at room temperature; for spectra recorded at higher temperatures, the resonance of residual solvent was set to 4.55 ppm for 320 K and 4.45 ppm for 330 K, according to ref. ^78^). For ^1^H NMR spectra recorded at high dilution, a water suppression program was used.

### UV–Vis absorption and fluorescence spectroscopy

UV–Vis absorption spectra were recorded on an Agilent Cary 60 spectrophotometer using quartz cuvettes with 1 and 0.4 cm pathways. Emission and excitation spectra were recorded on a Shimadzu RF-5301 PC spectrofluorophotometer.

### Isothermal titration calorimetry (ITC)

ITC experiments were performed on a MicroCal PEAQ-ITC instrument under ambient conditions (*T* = 25 °C). Stock solutions of **1** (2.34 mm), **2** (0.44 mm; deprotonated with 5 equiv of TMEDA), and **4** (0.44 mm) in double-distilled water were prepared (the solution of **1** was allowed to equilibrate for 1 day). The syringe was charged with 100 μL of the stock solution of **1** and the cell was charged with 300 μL of the stock solution of **2** or with 300 μL of the stock solution of **4**. In both titration experiments, 39 aliquots of the stock solution of **1** were injected; the first aliquot was 0.4 μL; the remaining 38 were 1 μL each. Each injection was carried out over 4 s, with the injections separated by 150 s. The stirring rate was 750 rpm, the feedback was set to high, and the reference power was 10 μW. The blanks (stock solution of **1** added to pure water) were also recorded and then subtracted from the titration data with the point-to-point tool. Subtracting the second blank (pure water added to a solution of the guest; verified for **4**) did not affect the quality of the fit. Data analysis was performed using MicroCal PEAQ-ITC Analysis Software; the Two Sets of Sites model was used to estimate the binding constants. The results are shown in Supplementary Figs. 17 and 49 for **2** and **4**, respectively.

### Mass spectrometry

High-resolution mass spectra of **3** were recorded on a Bruker Apex-Ultra FT-ICR mass spectrometer with electrospray ionization and negative-ion detection using water as the solvent.

### Transmission electron microscopy (TEM)

TEM images were acquired on a JEOL JEM-2100 microscope operating at 200 kV. Nanoparticle size and dispersity were determined using ImageJ.

### Synthesis of citrate-capped gold NPs

Citrate-capped gold NPs were synthesized following a previously reported literature procedure^79^. First, a stock solution of gold(III) was prepared by dissolving 40 mg of HAuCl_4_·3H_2_O in 4 mL of water. Gold seeds were obtained by injecting, under vigorous stirring, 0.5 mL of the gold(III) stock solution into 75 mL of a boiling water solution containing 42 mg of sodium citrate. After 10 min, the mixture was cooled down to 90 °C. Then, 0.5 mL of 60 mm sodium citrate solution and 0.5 mL of gold(III) stock solution were sequentially injected under continuous stirring, and the reaction mixture was kept at 90 °C for 30 min. Then, the injection of the same sodium citrate and gold(III) solutions were repeated and the mixture was stirred at 90 °C for an additional 30 min. Finally, the mixture was cooled down to room temperature by placing it in a water bath. The obtained NPs had a diameter of 21.2 (±3.2) nm (for representative TEM images, see Fig. 6a and Supplementary Fig. 51). The concentration of the final NP solution is 0.332 mm in terms of Au atoms and 1.13 nm in terms of NPs.

### Photoreduction experiments

As the light source, we used a Prizmatix 520 nm Ultra High Power Mic-LED light-emitting diode (LED) (collimated LED power of 900 mW). Double-distilled, degassed water was used as the solvent in all photoreduction experiments. Water was degassed by repeated freeze-pump-thaw cycles. In a typical experiment, 40 nmol **4** (free or in the presence of 20 nmol **1**, i.e., the **4**_2_⊂**1** complex) was dissolved in 4 mL of water. A 160 nmol of TMEDA and 9.0 × 10^−7^ nmol of citrate-capped Au NPs (0.265 nmol in terms of Au atoms; 0.8 μL of the stock solution) were added. The vial was purged with argon, tightly sealed, and exposed to a green LED. The progress of the reaction was monitored by UV–Vis absorption spectroscopy. For photoreduction of **2**/**4** on a paper (Fig. 6g, h), a picture of a flower was painted with a paintbrush for watercolor on a Strathmore 400 Series Mixed Media 300 g/m^2^ vellum paper. As the “paint”, we used aqueous solutions of **4** and **4**_2_⊂**1**. The concentrations of the solutions were 4.5 and 2.25 mm, respectively, corresponding to the same concentration of the dye. Then, the paper was uniformly soaked with TMEDA and citrate-capped Au NPs by applying an 18 mm TMEDA solution and a 0.11 nm NP solution using a paintbrush. Finally, the image was exposed to green light under irradiation conditions specified above.

### X-ray data collection and structure refinement

Single crystals of (**2**_2_⊂**1**)⋅**2**, **3**, and **6**_2_⊂**1** were obtained by slow evaporation of water from the aqueous solutions of **2**_2_⊂**1**, **3**, and **6**_2_⊂**1**, respectively. Single crystals of (**2**_2_⊂**1**)⋅**2**_4_ were obtained by slow diffusion of acetone vapors into an aqueous solution of **2**_2_⊂**1** in the presence of several equivalents of TMEDA. Single crystals of **4**_2_⊂**1** were obtained by slow diffusion of acetone vapors into an aqueous solution of **4**_2_⊂**1** in a refrigerator. Single crystals of (**4**_2_⊂**1**)⋅**4** were obtained by slow diffusion of acetone layered on top of an aqueous solution of **4**_2_⊂**1** in a refrigerator. Data collection was performed under a stream of nitrogen at 100 K using a Rigaku XtaLAB AFC12 (RINC) diffractometer or a Rigaku XtaLAB Synergy-R diffractometer using Cu-K_α_ radiation (1.54184 Å) (except **6**_2_⊂**1**, for which 0.71073 Å Mo-K_α_ radiation was used) and processed with CrysAlisPRO. The structures were solved by direct methods using SHELXT. All non-hydrogen atoms were further refined by SHELXL^80^. The crystal of **6**_2_⊂**1** was twinned and was treated as such. The SQUEEZE procedure^81^, implemented into PLATON^82^ or OLEX2^83^, was applied for disordered lattice solvent molecules, one heavily disordered nitrate in (**2**_2_⊂**1**)⋅**2**_4_, and two nitrates in **3**. Crystals of **3** were particularly unstable outside the mother liquor; thus, the single crystal was directly transferred to a diffractometer and frozen with the mother liquor (additional ice cover formed). The single crystal was twinned, but the separation of a single component did not improve the results. The crystal data and the structure refinement data are summarized in Supplementary Table 1.

## Supplementary information

Supplementary Information

Description of Additional Supplementary Files

Supplementary Data 1

## Data Availability

The datasets generated and/or analyzed during the current study are available from the corresponding authors on reasonable request. The X-ray crystallographic coordinates for structures reported in this Article have been deposited at the Cambridge Crystallographic Data Centre (CCDC), under deposition numbers CCDC 2045607, 2045611, 2048454, 2045608, 2045606, and 2045610. These data can be obtained free of charge from The Cambridge Crystallographic Data Centre via www.ccdc.cam.ac.uk/data_request/cif.
