# Supplementary material for: Encapsulation within a coordination cage modulates the reactivity of redox-active dyes
Source: Commun Chem. 2022 Mar 30;5:44. doi: 10.1038/s42004-022-00658-8 (PMC9814915; doi:10.1038/s42004-022-00658-8)
Supplement: Supplementary file 1 — Supplementary Information [file 42004_2022_658_MOESM1_ESM.pdf]

Supplementary Information for:

# Encapsulation within a coordination cage modulates the reactivity of redox-active dyes

Oksana Yanshyna,[a] Michał J. Białek,*[ab] Oleg V. Chashchikhin[a] and Rafal Klajn*[a]

[a]Department of Organic Chemistry, Weizmann Institute of Science, Rehovot 76100, Israel
[b]Department of Chemistry, University of Wrocław 14 F. Joliot-Curie St., 50383 Wrocław, Poland

## Table of contents

**Supplementary Methods** .......... 2
1. Synthesis and characterization of $\mathbf{2}_2\subset\mathbf{1}$ .......... 2
   1.1. NMR spectroscopy .......... 2
   1.2. Solid-state structure characterization .......... 9
   1.3. UV-Vis titration experiments .......... 11
   1.4. Fluorescence titration experiments .......... 12
   1.5. Isothermal titration calorimetry (ITC) .......... 12
2. DFT calculations of the 1:1 complex $\mathbf{2}\subset\mathbf{1}$ .......... 13
3. Synthesis and characterization of **3** .......... 13
   3.1. NMR spectroscopy .......... 14
   3.2. MS characterization .......... 21
4. Synthesis and characterization of $\mathbf{4}_2\subset\mathbf{1}$ .......... 22
   4.1. NMR spectroscopy .......... 22
   4.2. Solid-state structure characterization .......... 31
   4.3. UV-Vis titration experiments .......... 32
   4.4. Fluorescence titration experiments .......... 33
   4.5. Isothermal titration calorimetry (ITC) .......... 35
   4.6. VT-NMR spectroscopy .......... 36
5. Redox switching of free and encapsulated **2** and **4** .......... 37
   5.1. Control experiments .......... 37
   5.2. Reduction in the presence of extra cage .......... 41
6. Synthesis and characterization of $\mathbf{6}_2\subset\mathbf{1}$ .......... 42
   6.1. NMR spectroscopy .......... 42
   6.2. Solid-state structure characterization .......... 47
7. Crystal data and structure refinement data .......... 48
**Supplementary Note** Proving the existence of heterodimer $(\mathbf{2}\cdot\mathbf{4})\subset\mathbf{1}$ .......... 49
**Supplementary References** .......... 52

# Supplementary Methods

## 1. Synthesis and characterization of $\mathbf{2}_2\subset\mathbf{1}$

Resorufin **2** was dissolved in water using 1.5 equiv of TMEDA. To this solution, 0.5 equiv of cage **1** was added.

### 1.1. NMR spectroscopy characterization

$^1$H NMR (600 MHz, $D_2O$, 320 K): δ (ppm) = 9.25 (s, 8H, $\mathbf{1}_4$), 9.15 (s, 4H, $\mathbf{1}_1$), 7.88 (s, 4H, $\mathbf{1}_3$), 7.81 (s, 8H, $\mathbf{1}_7$), 7.66 (s, 4H, $\mathbf{1}_2$), 7.60 (s, 4H, $\mathbf{1}_8$), 7.58 (s, 8H, $\mathbf{1}_5$), 7.55 (s, 8H, $\mathbf{1}_6$), 5.38/5.37 (br, 4H, $\mathbf{2}_\beta$), 5.30 (s, 4H, $\mathbf{2}_\gamma$), 4.49 (br, 4H, $\mathbf{2}_\alpha$), 3.14 (s, 8H, $\mathbf{1}_{9,ax}$), 3.04 (s, 16H, $\mathbf{1}_{9,eq}$), 2.83 (s, 24H, $\mathbf{1}_{10,ax}$), 2.72 (s, 24H, $\mathbf{1}_{10,eq}$), 2.53 (s, 24H, $\mathbf{1}_{10,eq}$).

$^{13}$C NMR (151 MHz, $D_2O$, 320 K): δ (ppm) = 182.1 ($\mathbf{2}_\delta$), 148.3, 138.2, 137.7, 137.5 ($\mathbf{1}_4$), 136.7 ($\mathbf{1}_1$), 130.3, 130.1 ($\mathbf{2}_\alpha$), 129.9 ($\mathbf{1}_2$), 129.8, 129.5, 129.5 ($\mathbf{1}_5$), 126.0 ($\mathbf{2}_\beta$), 121.0 ($\mathbf{1}_3$), 120.9, 120.4, 120.3, 112.2 ($\mathbf{1}_8$), 111.4 ($\mathbf{1}_7$), 102.2 ($\mathbf{2}_\gamma$), 63.0 ($\mathbf{1}_{9,ax}$), 62.9 ($\mathbf{1}_{9,eq}$), 50.7 ($\mathbf{1}_{10,ax}$), 50.6 ($\mathbf{1}_{10,eq}$), 50.4 ($\mathbf{1}_{10,eq}$).

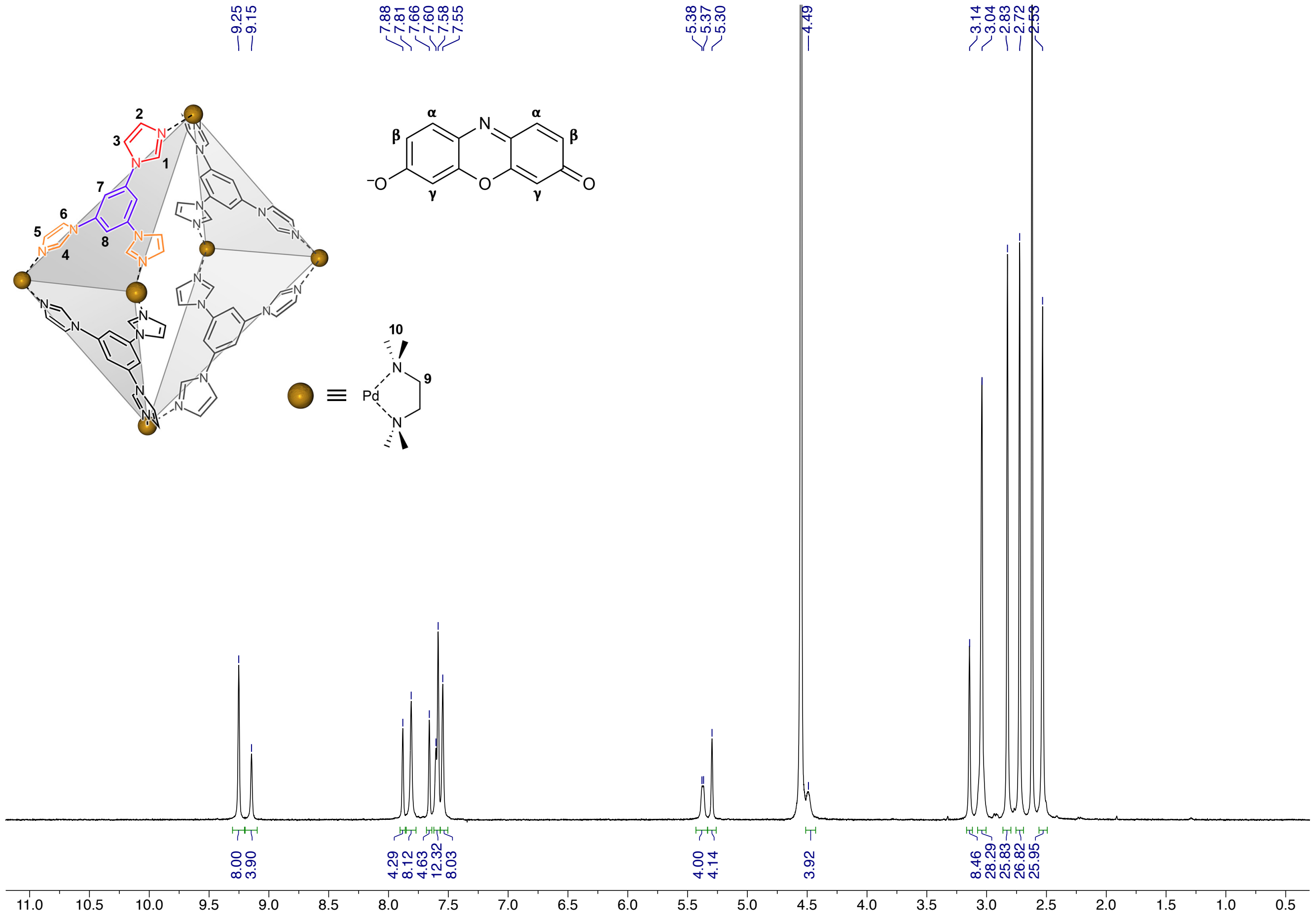


**Supplementary Fig. 1** $^1$H NMR spectrum of $\mathbf{2}_2\subset\mathbf{1}$ (600 MHz, $D_2O$, 320 K). The signal centered at 2.62 ppm originates from free TMEDA's C**H**$_3$ protons and integrates to ~37. The signal due to free TMEDA's C**H**$_2$ protons is centered at 3.04 ppm and overlaps with **1**'s equatorial TMEDA C**H**$_2$ protons ($\mathbf{1}_{9,eq}$).

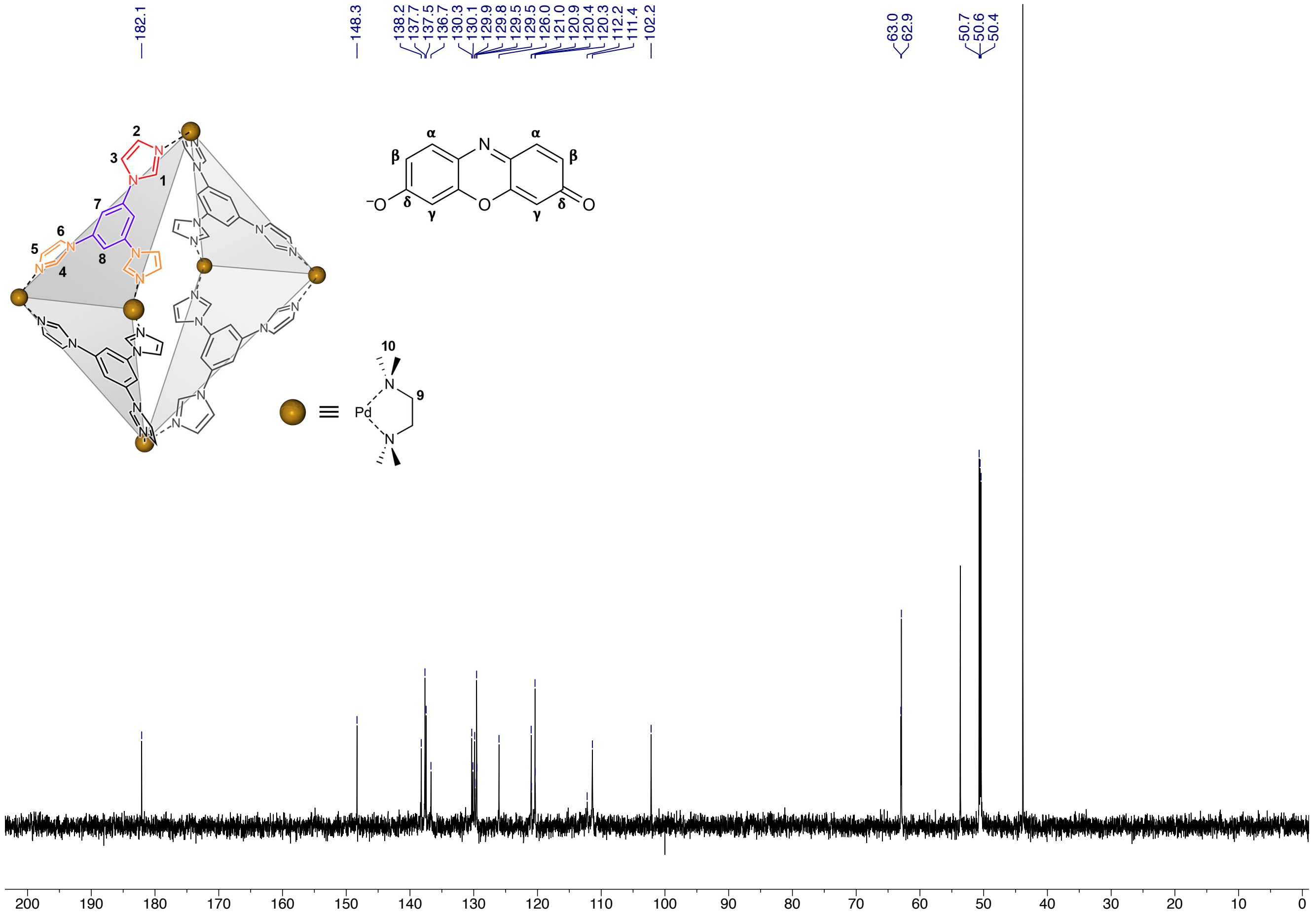


**Supplementary Fig. 2** $^{13}$C NMR spectrum of $\mathbf{2}_2\subset\mathbf{1}$ (151 MHz, $D_2O$, 320 K).

The peak assignment in the NMR spectra of $\mathbf{2}_2\subset\mathbf{1}$ is based on $^{1}$H, $^{13}$C, and $^{1}$H–$^{13}$C HSQC spectra of **2** in $D_2O$, which are shown below (**2** was solubilized in water upon deprotonation with TMEDA).

$^{1}$H NMR (600 MHz, $D_2O$, 320 K): δ (ppm) = 7.46 (d, $J$ = 9.2 Hz, 2H, $\mathbf{2}_{\alpha}$), 6.76 (dd, $J$ = 9.2, 2.4 Hz, 2H, $\mathbf{2}_{\beta}$), 6.40 (d, $J$ = 2.3 Hz, 2H, $\mathbf{2}_{\gamma}$).

$^{13}$C NMR (151 MHz, $D_2O$, 320 K): δ (ppm) = 182.9 ($\mathbf{2}_{\delta}$), 150.4, 132.7 ($\mathbf{2}_{\alpha}$), 131.4, 126.1 ($\mathbf{2}_{\beta}$), 103.7 ($\mathbf{2}_{\gamma}$).

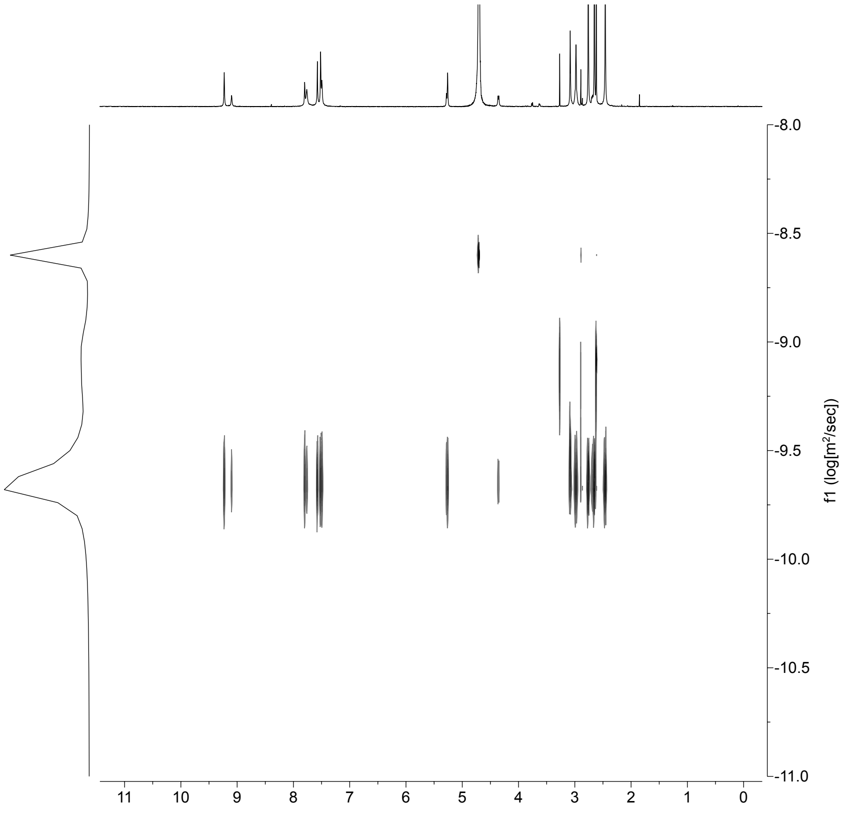


**Supplementary Fig. 3** $^{1}$H DOSY NMR spectrum of $\mathbf{2}_2\subset\mathbf{1}$ (600 MHz, $D_2O$, 300 K).

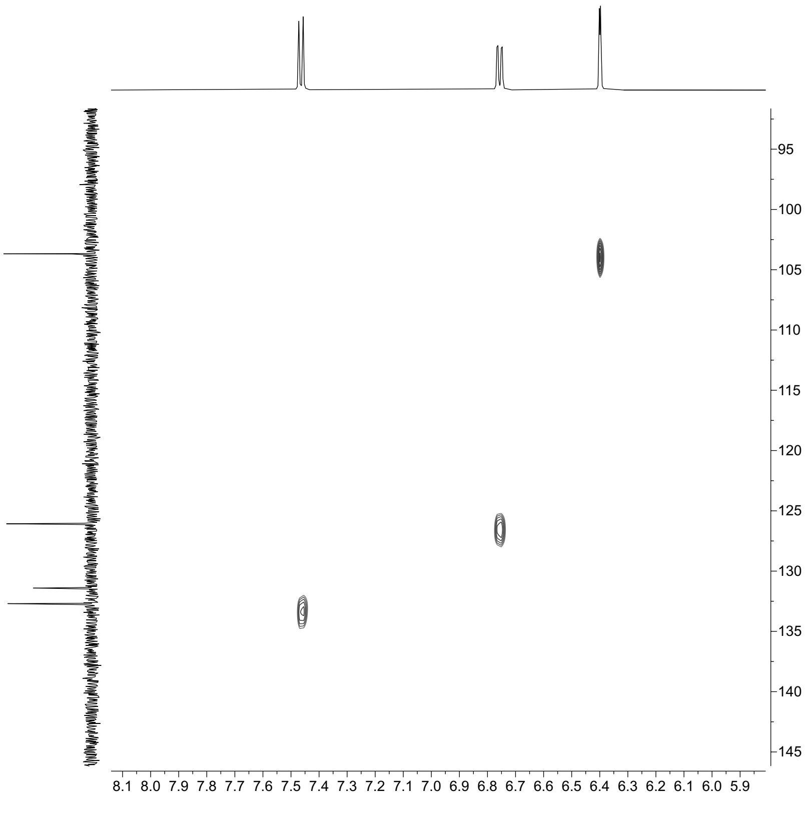


**Supplementary Fig. 4** $^{1}$H–$^{13}$C HSQC NMR spectrum of **2** (TMEDAH$^{+}$ salt) (600 MHz, $D_2O$, 320 K).

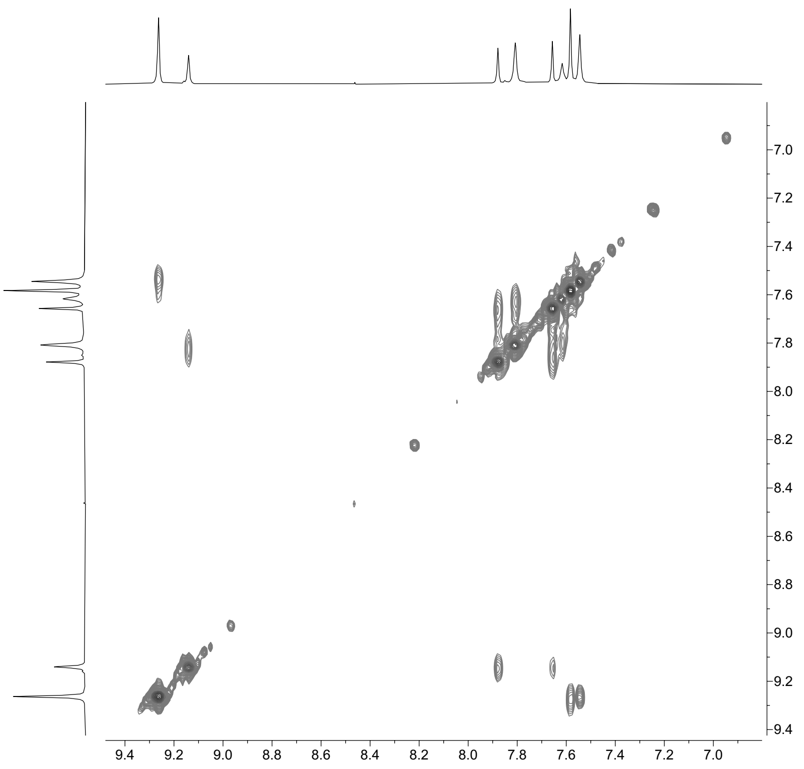


**Supplementary Fig. 5** Partial $^{1}$H–$^{1}$H COSY NMR spectrum of $\mathbf{2}_2\subset\mathbf{1}$ (600 MHz, $D_2O$, 320 K).

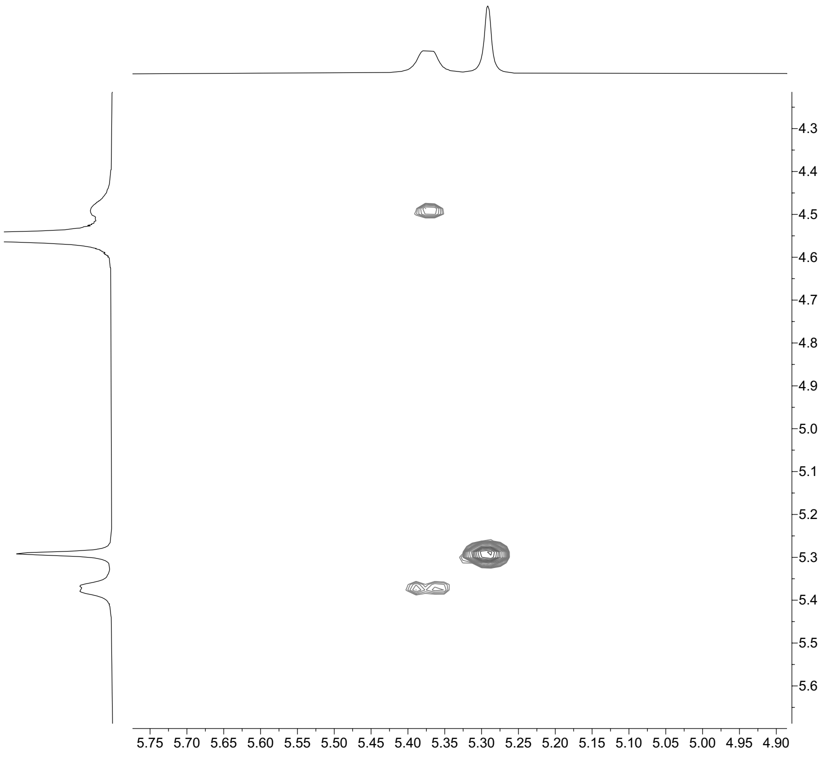


**Supplementary Fig. 6** Partial $^{1}$H–$^{1}$H COSY NMR spectrum of $\mathbf{2}_2\subset\mathbf{1}$ (600 MHz, $D_2O$, 320 K).

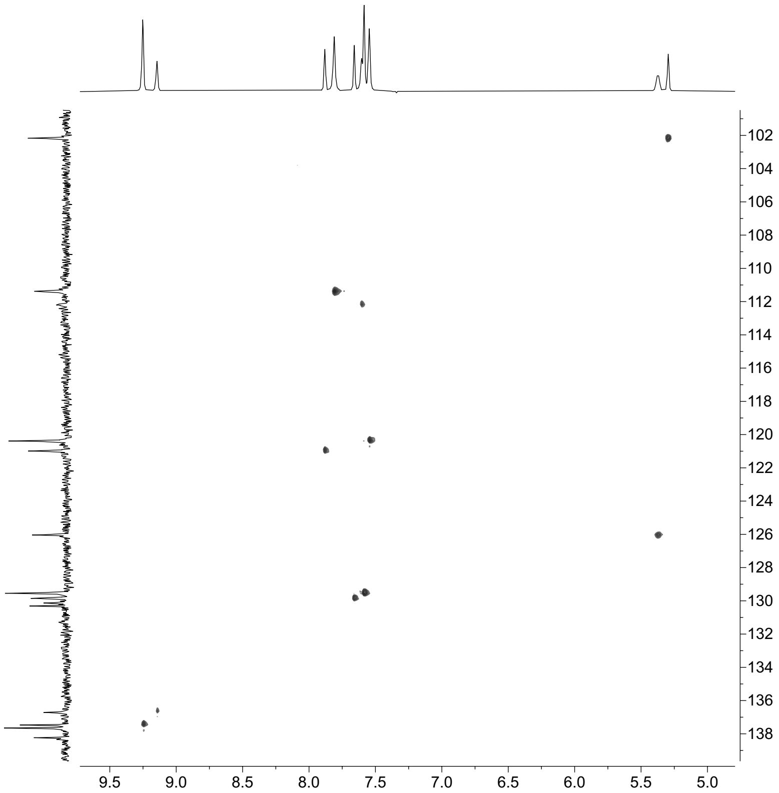


**Supplementary Fig. 7** Partial $^{1}H$–$^{13}C$ HSQC NMR spectrum of $\mathbf{2}_2\subset\mathbf{1}$ (600 MHz, $D_2O$, 320 K).

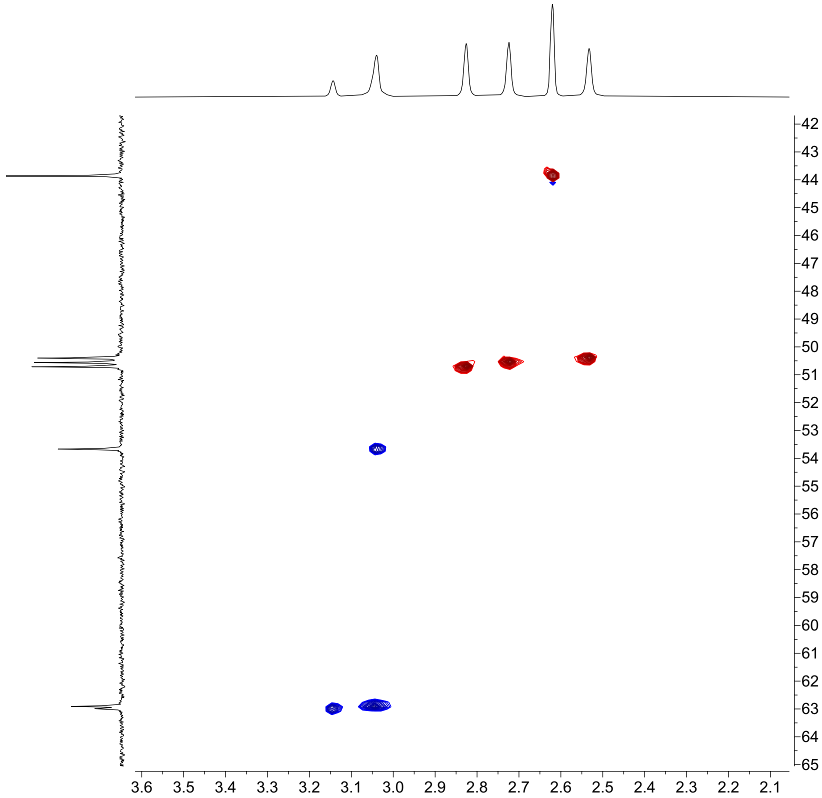


**Supplementary Fig. 8** Partial $^{1}H$–$^{13}C$ HSQC NMR spectrum of $\mathbf{2}_2\subset\mathbf{1}$ (600 MHz, $D_2O$, 320 K). Note that two signals merge at ~3.04 ppm: one due to free TMEDA's C**H**$_2$ protons and one due to **1**'s equatorial TMEDA C**H**$_2$ protons ($\mathbf{1}_{\mathbf{9},eq}$).

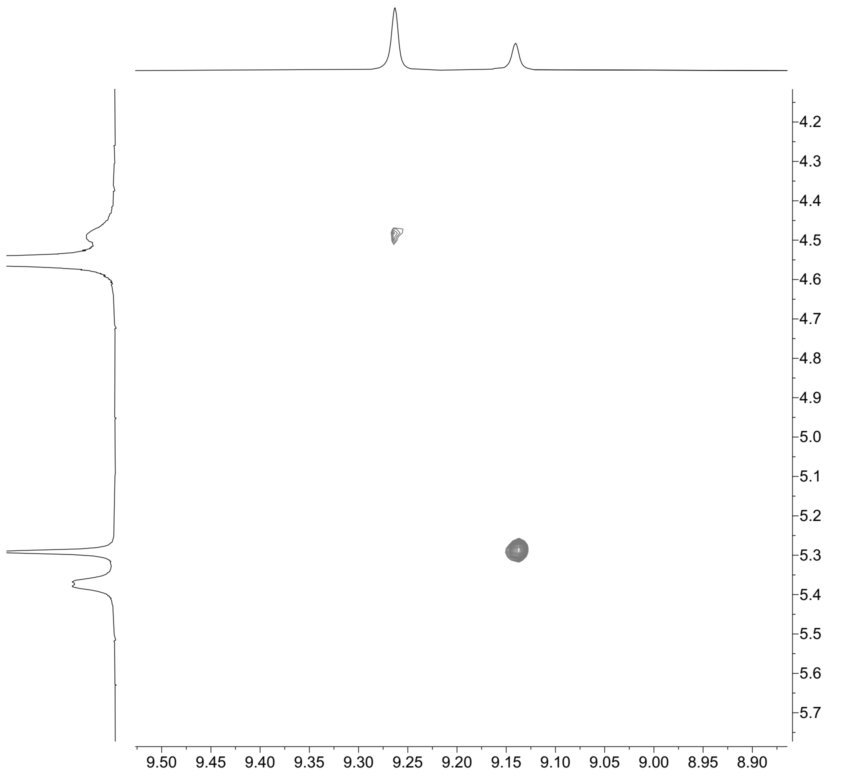


**Supplementary Fig. 9** Partial $^{1}$H–$^{1}$H NOESY NMR spectrum of $\mathbf{2}_2\subset\mathbf{1}$ (600 MHz, $D_2O$, 320 K).

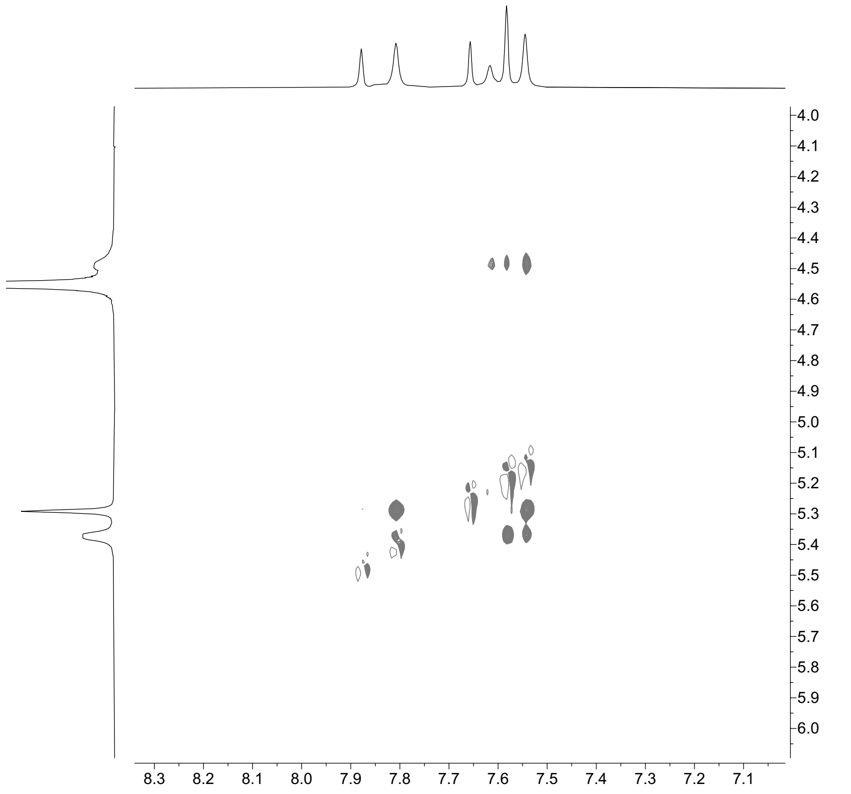


**Supplementary Fig. 10** Partial $^{1}$H–$^{1}$H NOESY NMR spectrum of $\mathbf{2}_2\subset\mathbf{1}$ (600 MHz, $D_2O$, 320 K).

To facilitate the interpretation of the 2D NMR data shown above, $^{1}H$ and $^{13}C$ spectra of free **2** are included below.

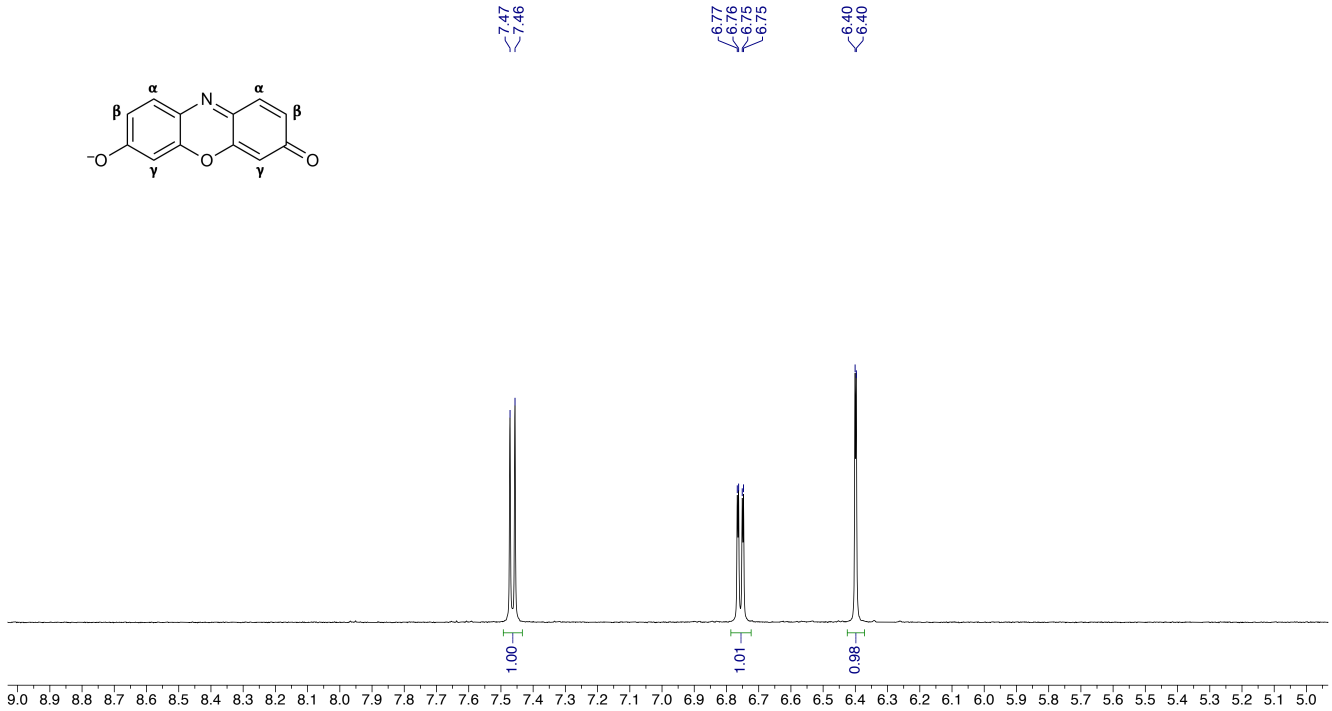


**Supplementary Fig. 11** $^{1}H$ NMR spectrum of **2** (TMEDAH$^{+}$ salt) (600 MHz, $D_2O$, 320 K).

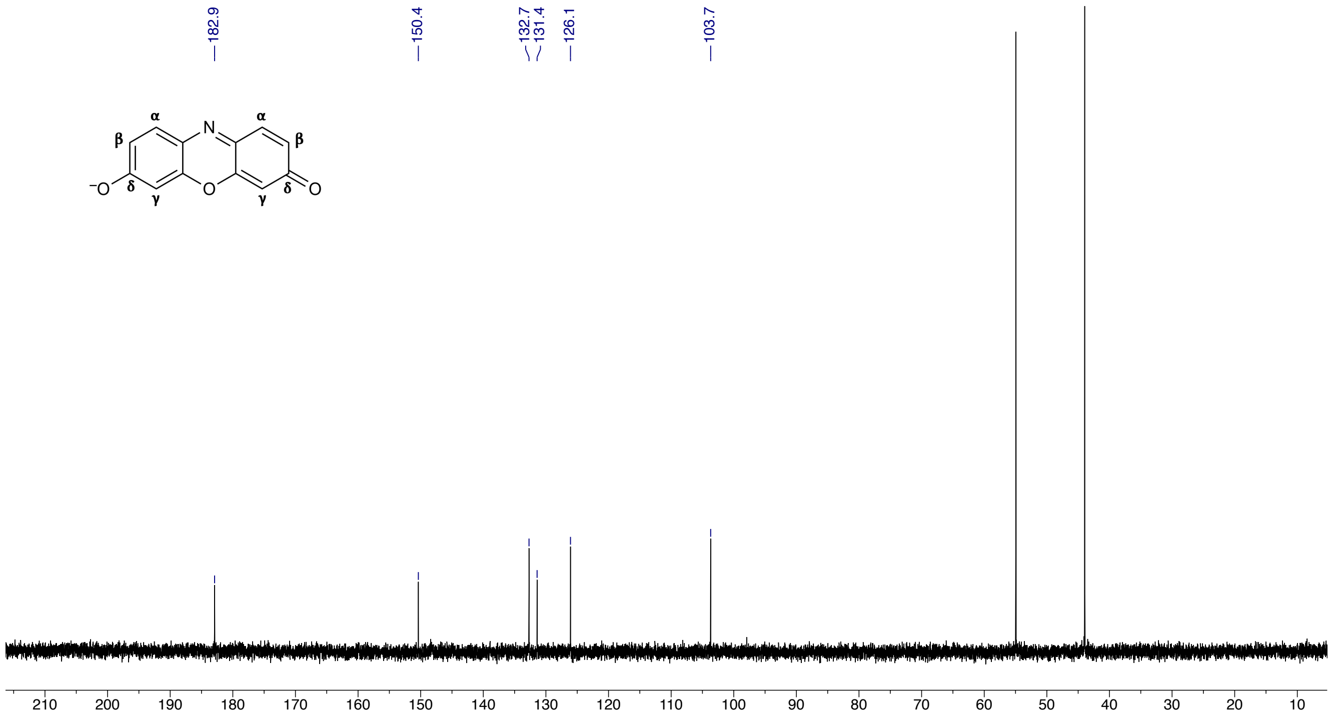


**Supplementary Fig. 12** $^{13}C$ NMR spectrum of **2** (TMEDAH$^{+}$ salt) (151 MHz, $D_2O$, 320 K).

## 1.2. Solid-state structure characterization

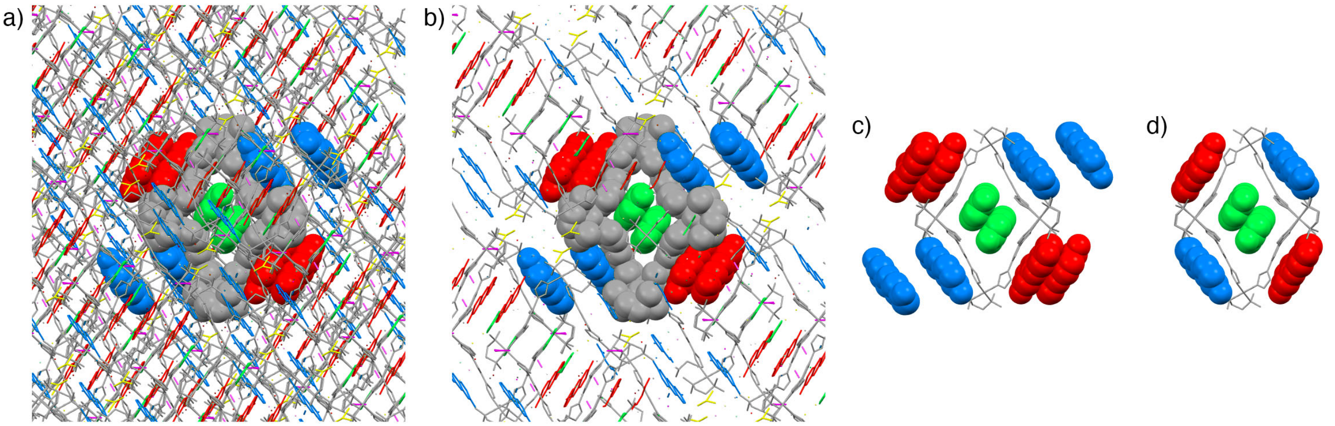


**Supplementary Fig. 13 a–d** Snapshots from the crystal structure of $(\mathbf{2}_2\subset\mathbf{1})\cdot\mathbf{2}_4$ showing different levels of structural detail. Cage **1** is denoted in gray, **2** encapsulated inside the cage in green, and **2** residing between cages in red and blue (hydrogens omitted for clarity). The snapshots show the same view of the structure, but with an increasingly larger number of elements removed. **d** The smallest repeating unit (with water molecules and nitrate ions omitted for clarity). Molecules of **2** residing outside the cages assume two different configurations. The molecules oriented parallel to the encapsulated **2** (shown in red) are in close contact with each other (plane-to-plane distance = 3.334 Å; compare with the plane-to-plane distance of 3.310 Å for the encapsulated **2**, shown in green), giving rise to extended $[\mathbf{2}_{red}\cdots\mathrm{TImB}\cdots\mathbf{2}_{green}\cdots\mathbf{2}_{green}\cdots\mathrm{TImB}\cdots\mathbf{2}_{red}]_\infty$ π–π stacks. In contrast, the molecules oriented perpendicular to the encapsulated **2** (blue) are separated by a relatively large distance (6.653 Å), with a monolayer of disordered nitrates between them.

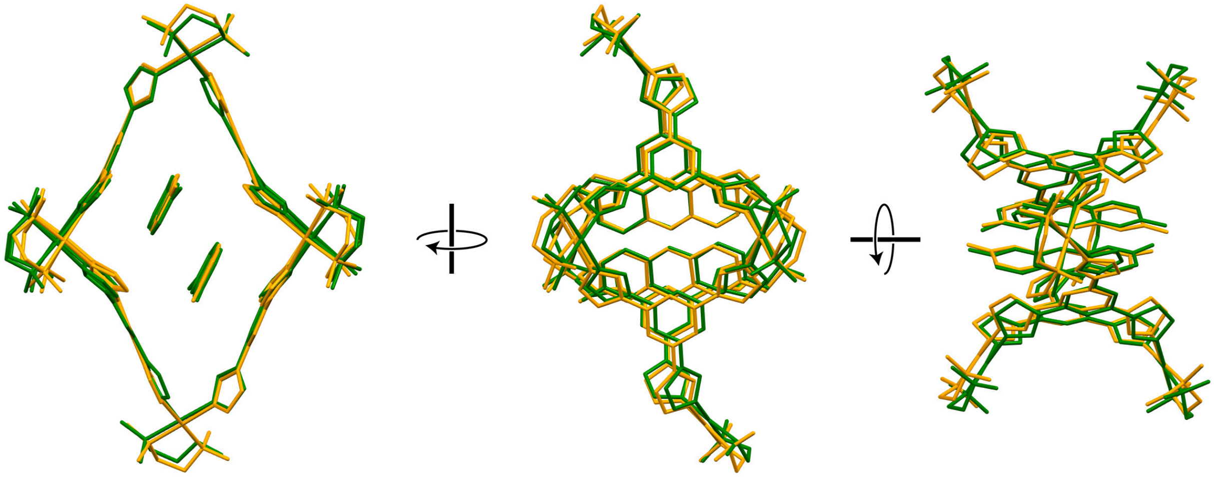

**Supplementary Fig. 14** Overlay of the crystal structures of $\mathbf{2}_2\subset\mathbf{1}$ extracted from $(\mathbf{2}_2\subset\mathbf{1})\cdot\mathbf{2}$ (yellow) and $(\mathbf{2}_2\subset\mathbf{1})\cdot\mathbf{2}_4$ (green). Hydrogen atoms, unencapsulated **2**, nitrates, and water molecules were omitted for clarity).

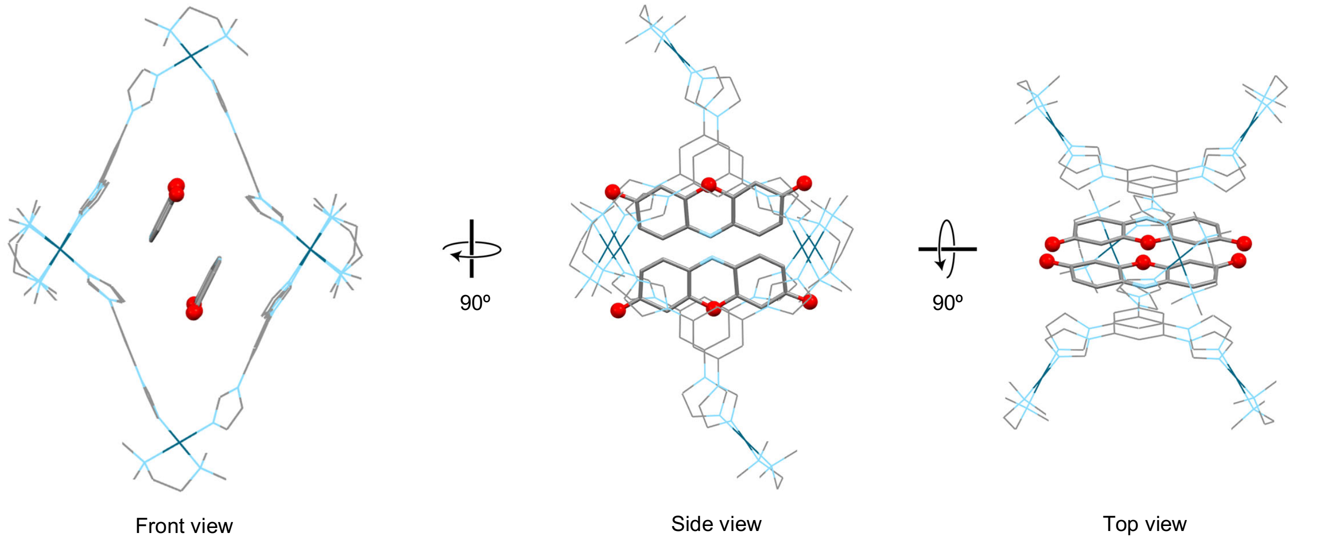


**Supplementary Fig. 15** X-ray structure of the $\mathbf{2}_2\subset\mathbf{1}$ inclusion complex (here, extracted from the solid-state structure of $(\mathbf{2}_2\subset\mathbf{1})\cdot\mathbf{2}$) along three different viewing directions. The oxygen atoms are indicated as red spheres to highlight the antiparallel alignment of the two guests. Hydrogens, nitrates, solvent molecules, and **2** residing outside the **1**'s cavity were omitted for clarity. Pd, dark-blue; C, gray; N, light-blue.

## 1.3. UV-Vis titration experiments

*Titration of resorufin with cage **1***: Titration experiments were carried out in double-distilled water. Cage **1** (10 mg) was dissolved in water (0.5 mL) and the solution was allowed to equilibrate for one day. 10 mg of **2** was dissolved in 5 mL of water containing 27 µL TMEDA (to ensure deprotonation and solubilization. 2 µL of the resulting solution was diluted with 1 mL of water and titrated with small aliquots of **1** (1.5 µL), each corresponding to 0.05 equiv of the guest with respect to the cage. The injection rate was 2 min per aliquot; after each injection, a UV-Vis absorption spectrum was recorded. Titration was continued until 1.0 equiv of **1** was added. The resulting UV-Vis absorption spectra are shown in Fig. 3b.

*Titration of cage **1** with resorufin*: Titration experiments were carried out in double-distilled water. Cage **1** (15 mg) was dissolved in water (0.5 mL) and the solution was allowed to equilibrate for one day. 0.65 µL of the resulting solution was diluted with 1 mL of water. TMEDA was used as the base to deprotonate and solubilize **2** in water. Specifically, 10 mg of **2** was dissolved in 5 mL of water containing 29 µL of TMEDA. The dilute solution of **1** was titrated with small aliquots of **2** (0.137 µL), each corresponding to 0.2 equiv with respect to **1**. The injection rate was 2 min per aliquot; after each injection, a UV-Vis absorption spectrum was recorded. Titration was continued until 3.0 equiv of **2** were added. The resulting UV-Vis absorption spectra are shown in Fig. 2b.

We repeated the titration of the dilute solution of **1** with **2** until 2.0 equiv of **2** were added (at this point of titration, UV-Vis absorption spectra consistently showed significant amounts of unencapsulated **2**; Supplementary Fig. 15a). We then let the solution equilibrate for 20 h and remeasured the spectrum, obtaining the green spectrum in Supplementary Fig. 16a. Analysis of the spectra (Supplementary Fig. 16b) showed that the amount of free **2** significantly decreases over 20 h at the expense of encapsulated **2**, which we attribute to the ability of **2** to template the formation of the cage.

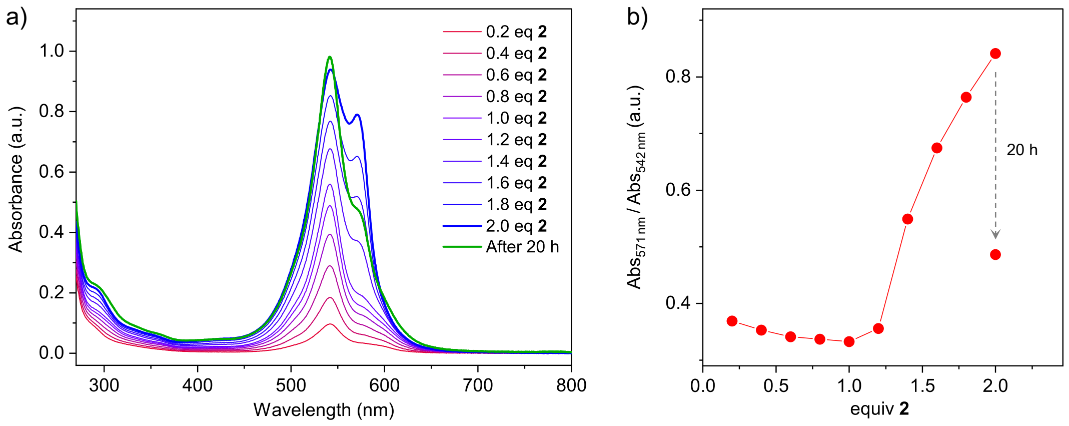


**Supplementary Fig. 16** Resorufin-templated reassembly of cage **1** followed by UV-Vis absorbance spectroscopy. **a)** Evolution of UV-Vis spectra of cage **1** upon the gradual addition of **2**. Once 2.0 equiv of **2** were added (thick blue line), the solution was allowed to equilibrate for 20 h, resulting in the spectrum shown in green. **b)** Analysis of the UV-Vis spectra. High absorbance at 571 nm indicates the presence of free **2**.

## 1.4. Fluorescence titration experiments

*Titration of resorufin with cage **1***: Titration experiments were carried out in double-distilled water. Cage **1** (10 mg) was dissolved in water (0.5 mL) and the solution was allowed to equilibrate for one day. 10 mg of **2** was dissolved in 5 mL of water containing 27 µL TMEDA (to ensure deprotonation and solubilization. 2 µL of the resulting solution were diluted with 1 mL of water and titrated with small aliquots of **1** (1.5 µL), each corresponding to 0.05 equiv of the guest with respect to the cage. The injection rate was 2 min per aliquot; after each injection, a fluorescence spectrum was recorded (excitation wavelength = 530 nm). Titration was continued until 1.0 equiv of **1** was added. The resulting fluorescence spectra are shown in Fig. 2d in the main text.

*Titration of cage **1** with resorufin*: Titration experiments were carried out in double-distilled water. Cage **1** (15 mg) was dissolved in water (0.5 mL) and the solution was allowed to equilibrate for one day. 0.65 µL of the resulting solution was diluted with 1 mL of water. TMEDA was used as the base to deprotonate and solubilize **2** in water. Specifically, 10 mg of **2** were dissolved in 5 mL of water containing 29 µL of TMEDA. The diluted solution of **1** was titrated with small aliquots of **2** (0.137 µL), each corresponding to 0.2 equiv of **2** with respect to **1**. The injection rate was 2 min per aliquot; after each injection, a fluorescence spectrum was recorded (excitation wavelength = 530 nm). Titration was continued until 2.0 equiv of **2** were added. The resulting fluorescence spectra are shown in Fig. 3d in the main text.

## 1.5. Isothermal titration calorimetry (ITC)

For the description of the experiments, see the Methods section.

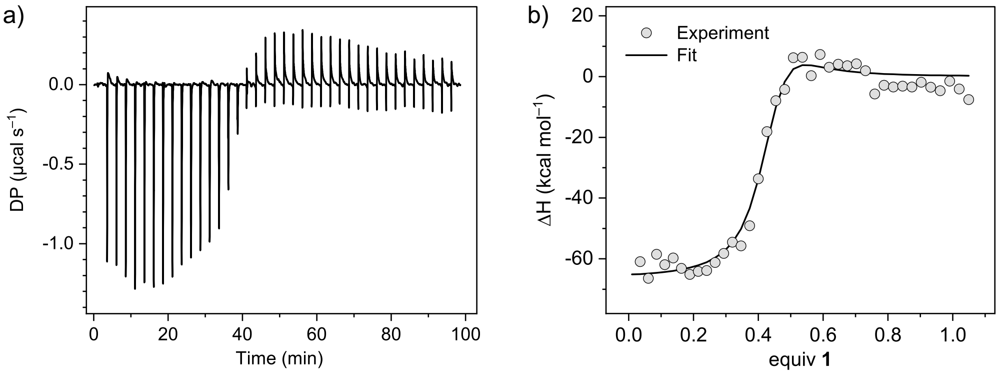


**Supplementary Fig. 17 a)** A representative plot of differential power (DP) vs. time for the titration of **2** (0.44 mM) with cage **1** (2.34 mM). **b)** The resulting plot of ΔH vs. the amount of **1** added, fitted to a 2:1 binding model.

## 2. DFT calculations of the 1:1 complex **2⊂1**

Two models were calculated, starting from either vertically (**2⊂1**) or horizontally (**2⊂1′**) aligned resorufin guest inside the cage (surrounded by 10 symmetrically distributed nitrates; overall a monocationic species). The models were optimized using DFT methods in Gaussian 16 using a dispersion-corrected B3LYP functional with a 6-31G(d,p)/LANL2DZ(Pd) basis set. The two models represent local energy minima (confirmed by frequency calculations), with **2⊂1** (Supplementary Fig. 18) more stable than **2⊂1′** (Supplementary Fig. 19) by 6.7 kcal/mol.

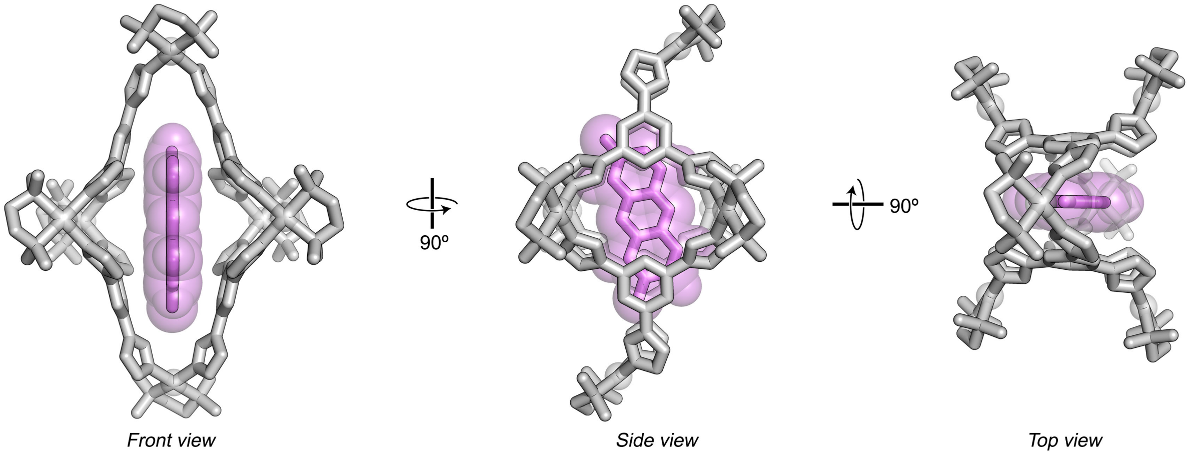


**Supplementary Fig. 18** Energy-optimized structure of the putative 1:1 complex **2⊂1**, calculated starting with a vertical orientation of **2** within **1**. Hydrogen atoms and nitrates were omitted for clarity.

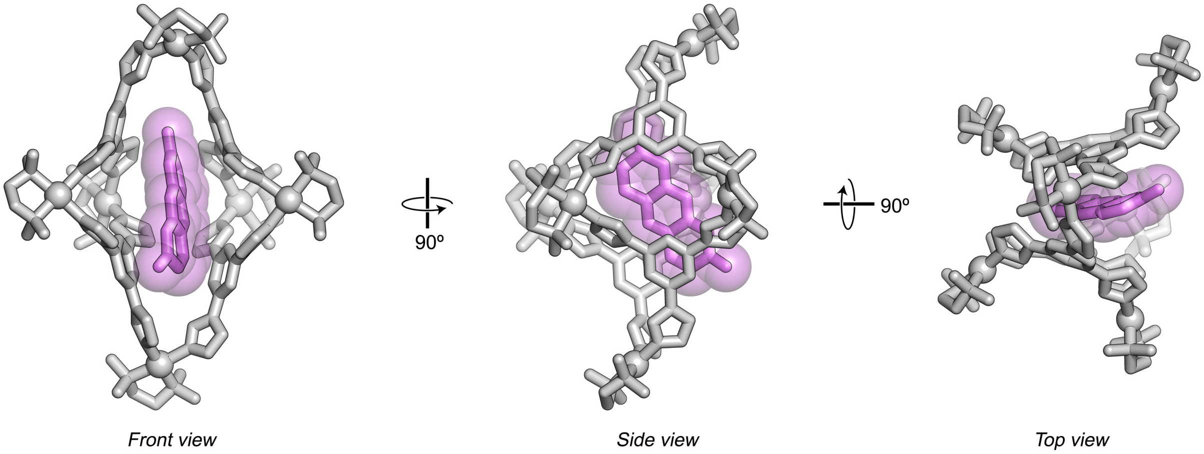


**Supplementary Fig. 19** Energy-optimized structure of the putative 1:1 complex **2⊂1′**, calculated starting with a horizontal orientation of **2** within **1**. Hydrogen atoms and nitrates were omitted for clarity.

## 3. Synthesis of and characterization of **3**

TMEDA (50 mg; 0.43 mmol) was dissolved in DMSO (6 mL), and $Pd(NO_3)_2 \cdot xH_2O$ (107 mg; 0.43 mmol) was added. The mixture was heated at 80 °C until all $Pd(NO_3)_2$ was dissolved (~10 min). Then, TImB (119 mg; 0.43 mmol) was added and the reaction mixture was heated at 80 °C for an additional 2 h. The resulting mixture was filtered to remove dark solids and ethyl acetate (25 mL) was added to the filtrate to precipitate the crude product

(mixture of **3** with cage **1**). Compound **3** was purified from **1** based on its low water solubility. Specifically, the mixture of solid **3** and **1** was treated with a minimal amount of water and the undissolved fraction was collected by filtration (the process was repeated if needed).

## 3.1. NMR spectroscopy characterization

$^1$H NMR (600 MHz, DMSO-*d*$_6$, 300 K): δ (ppm) = 9.51 (4H, $\mathbf{3}_4$), 8.52 (2H, $\mathbf{3}_1$), 8.26 (4H, $\mathbf{3}_6$), 8.09 (2H, $\mathbf{3}_8$), 8.07 (4H, $\mathbf{3}_7$), 7.99 (2H, $\mathbf{3}_3$), 7.66 (4H, $\mathbf{3}_5$), 7.21 (2H, $\mathbf{3}_2$), 3.05 (8H, $\mathbf{3}_9$), 2.66 (24H, $\mathbf{3}_{10}$).

$^1$H NMR (600 MHz, $D_2O$, 300 K): δ (ppm) = 9.17 (4H, $\mathbf{3}_4$), 8.25 (2H, $\mathbf{3}_1$), 7.89 (4H, $\mathbf{3}_6$), 7.81 (2H, $\mathbf{3}_8$), 7.79 (4H, $\mathbf{3}_7$), 7.68 (2H, $\mathbf{3}_3$), 7.63 (4H, $\mathbf{3}_5$), 7.27 (2H, $\mathbf{3}_2$), 3.15 (8H, $\mathbf{3}_9$), 2.79 (24H, $\mathbf{3}_{10}$).

$^{13}$C NMR (151 MHz, DMSO-*d*$_6$, 300 K): δ (ppm) = 139.0, 137.6, 137.4 ($\mathbf{3}_4$), 136.0 ($\mathbf{3}_1$), 130.5 ($\mathbf{3}_3$), 128.8 ($\mathbf{3}_5$), 120.3 ($\mathbf{3}_6$), 118.1 ($\mathbf{3}_2$), 111.1 ($\mathbf{3}_7$/$\mathbf{3}_8$), 110.8 ($\mathbf{3}_7$/$\mathbf{3}_8$), 62.2 ($\mathbf{3}_9$), 50.2 ($\mathbf{3}_{10}$).

Note that the peaks in the $^1$H NMR spectrum of **3** in DMSO are broadened and appear as singlets (in particular, for the imidazole group not coordinating to $Pd^{2+}$). Also note that the assignment of protons $\mathbf{3}_2$ vs. $\mathbf{3}_3$ could not be made unambiguously based on the spectra obtained; the assignment was made based on the observation that in the other pair of imidazole protons ($\mathbf{3}_5$ and $\mathbf{3}_6$), the imidazole proton more distant from the central benzene ring ($\mathbf{3}_5$) is more upfield shifted; the same is true with both pairs of imidazole protons in free cage **1** and in free TImB.[1]

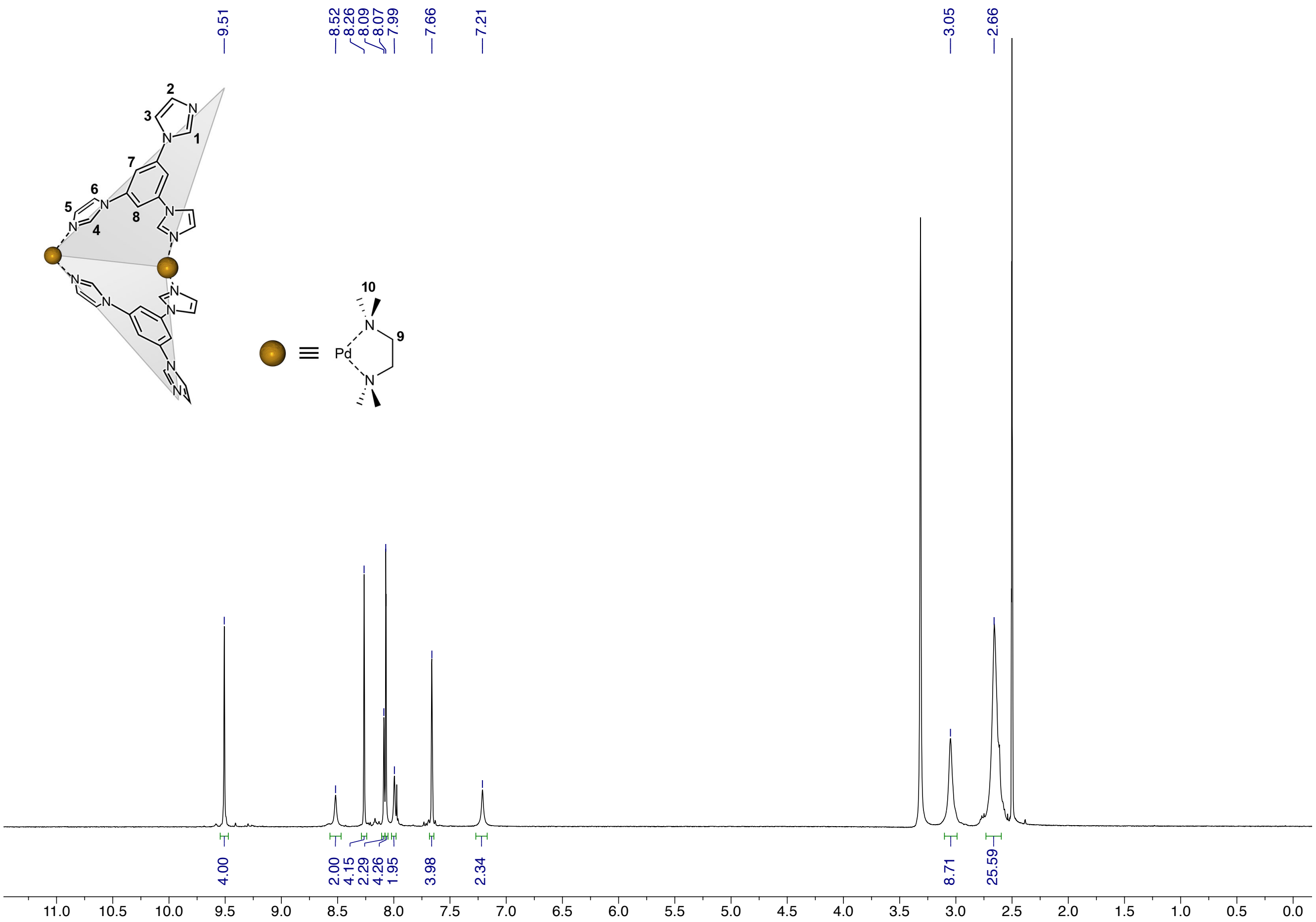


**Supplementary Fig. 20** $^1$H NMR spectrum of **3** (600 MHz, DMSO-*d*$_6$, 300 K).

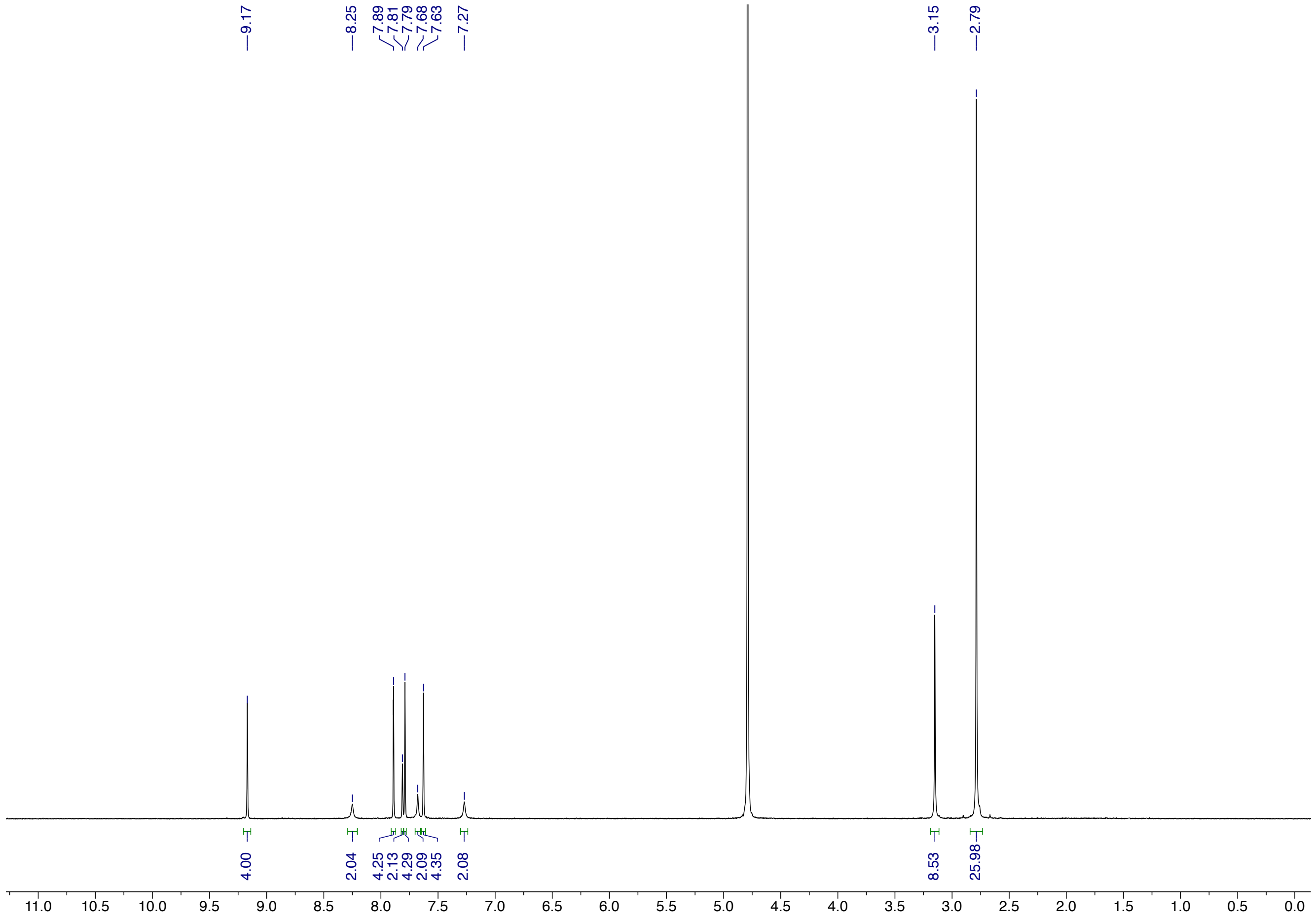


**Supplementary Fig. 21** $^1$H NMR spectrum of **3** (600 MHz, $D_2O$, 300 K).

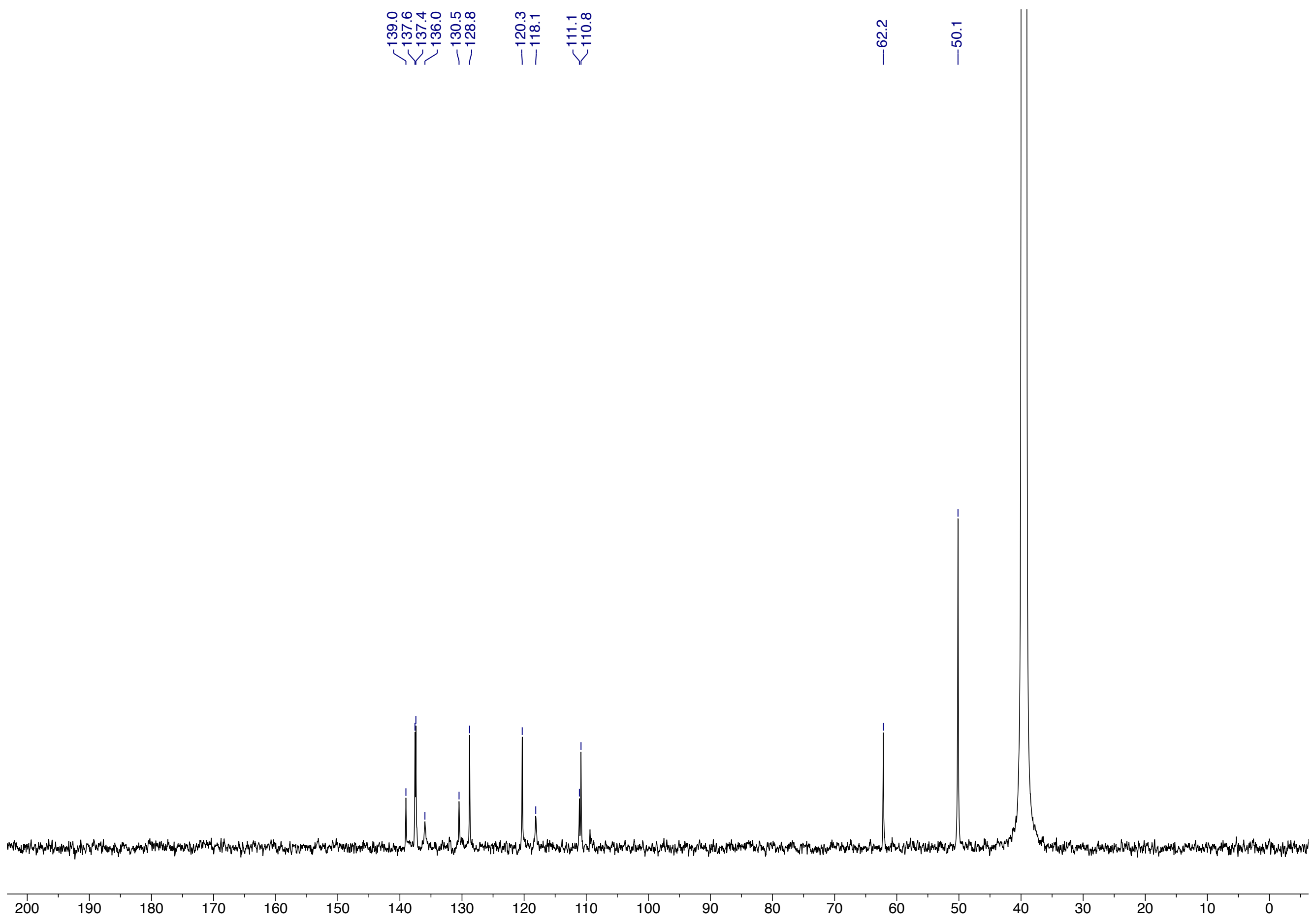


**Supplementary Fig. 22** $^{13}$C NMR spectrum of **3** (151 MHz, DMSO-$d_6$, 300 K).

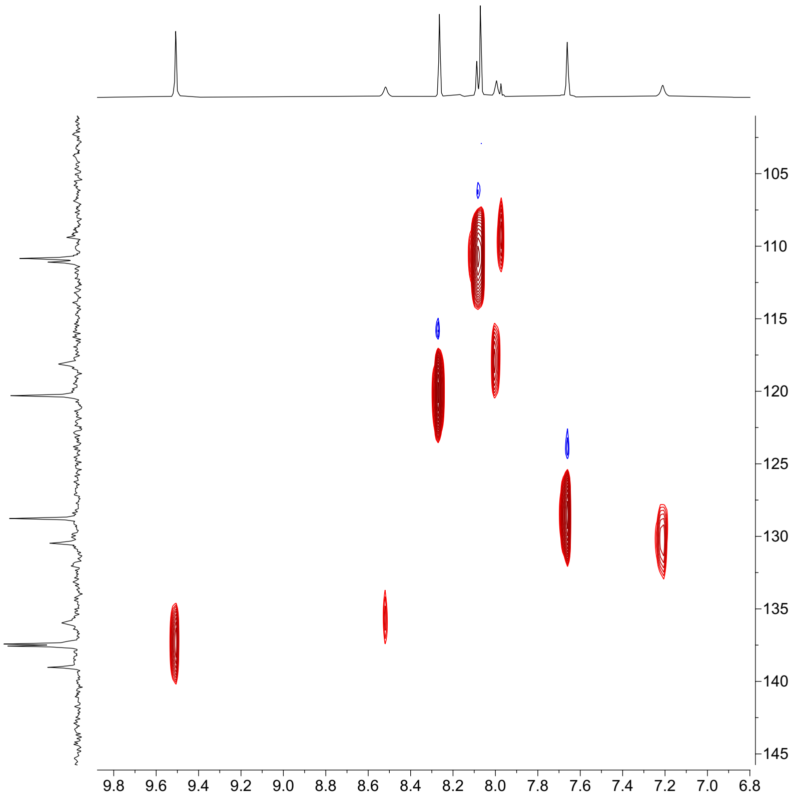


**Supplementary Fig. 23** Partial $^{1}$H–$^{13}$C HSQC NMR spectrum of **3** (600 MHz, DMSO-$d_6$, 300 K).

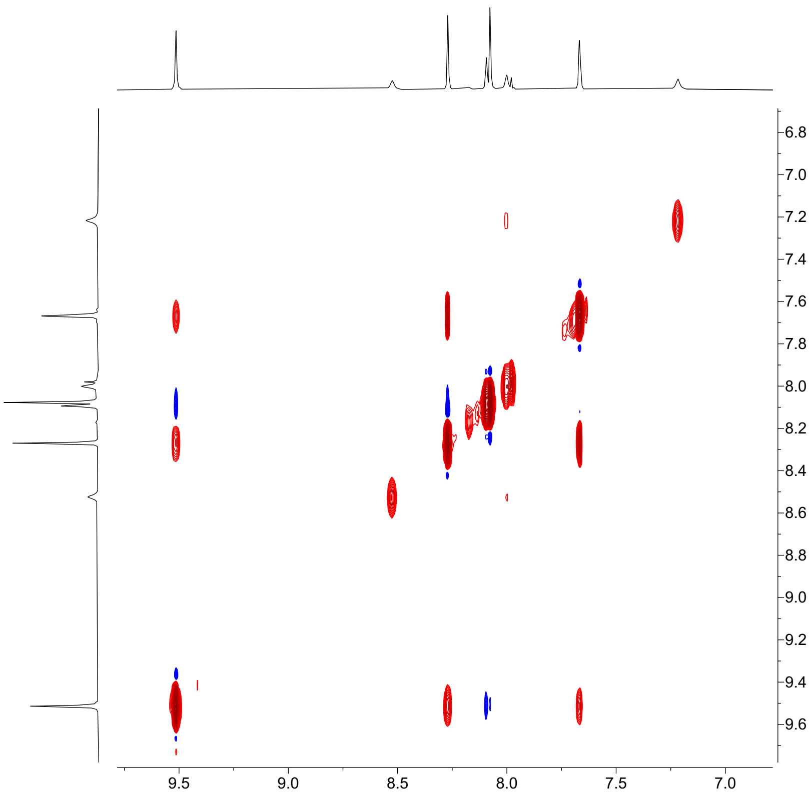


**Supplementary Fig. 24** Partial $^{1}$H–$^{1}$H TOCSY NMR spectrum of **3** (600 MHz, DMSO-$d_6$, 300 K).

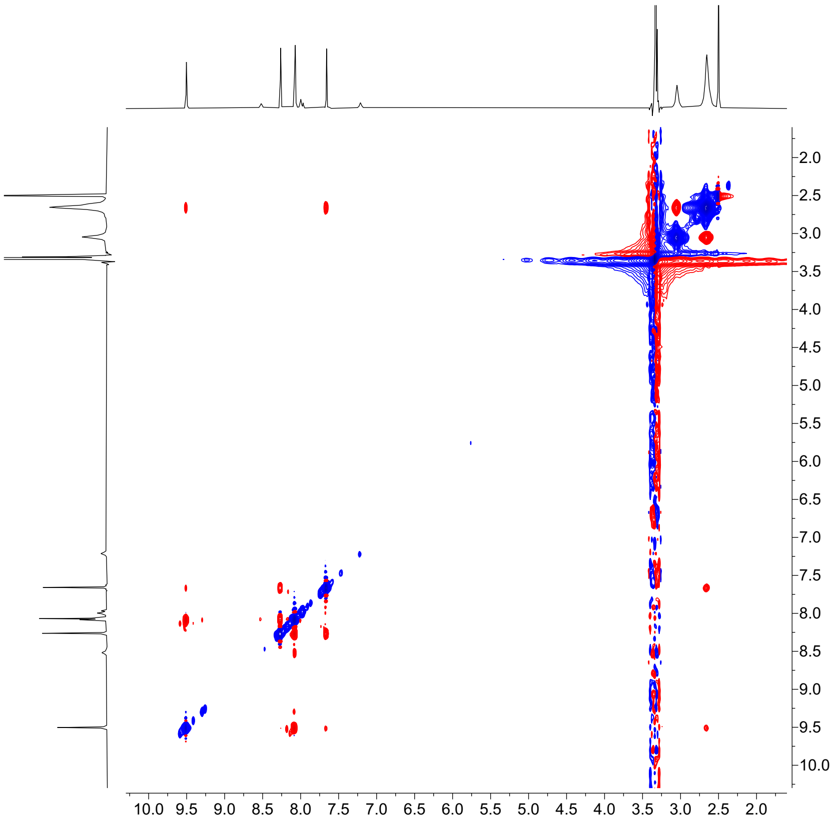


**Supplementary Fig. 25** $^{1}$H–$^{1}$H ROESY NMR spectrum of **3** (600 MHz, DMSO-$d_6$, 300 K).

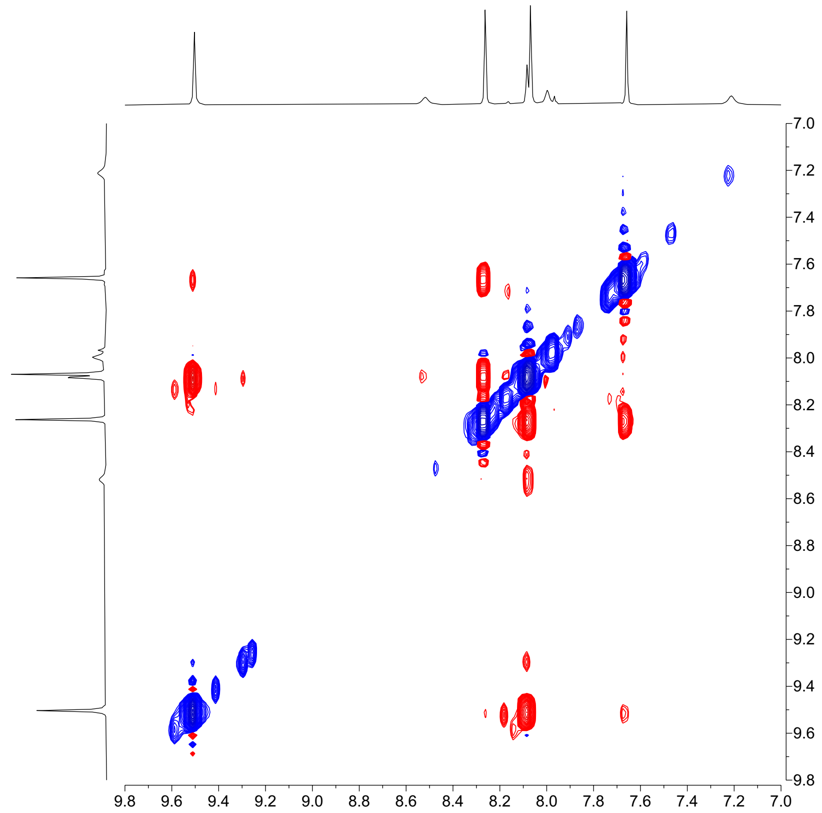


**Supplementary Fig. 26** Partial $^{1}$H–$^{1}$H ROESY NMR spectrum of **3** (600 MHz, DMSO-$d_6$, 300 K).

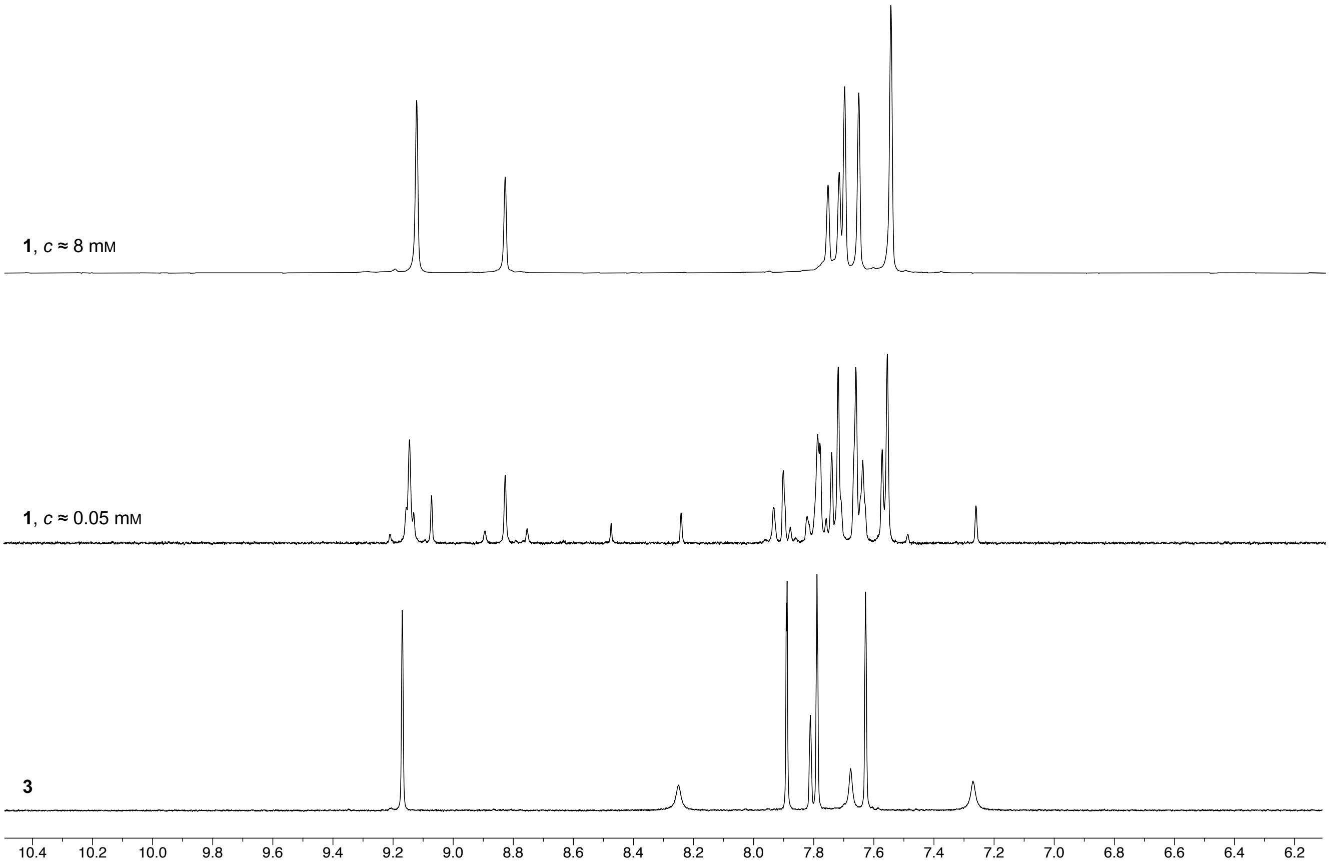


**Supplementary Fig. 27** $^{1}$H NMR spectra of **1** recorded at a high concentration (top) and at a low concentration (center) and an $^{1}$H NMR spectrum of **3** (bottom). Note the characteristic peaks of **3** at ~8.25 ppm and ~7.27 ppm, which are absent in the spectrum of concentrated **1**, but appear in the spectrum of diluted **1** (all spectra recorded at 600 MHz, $D_2O$, 300 K).

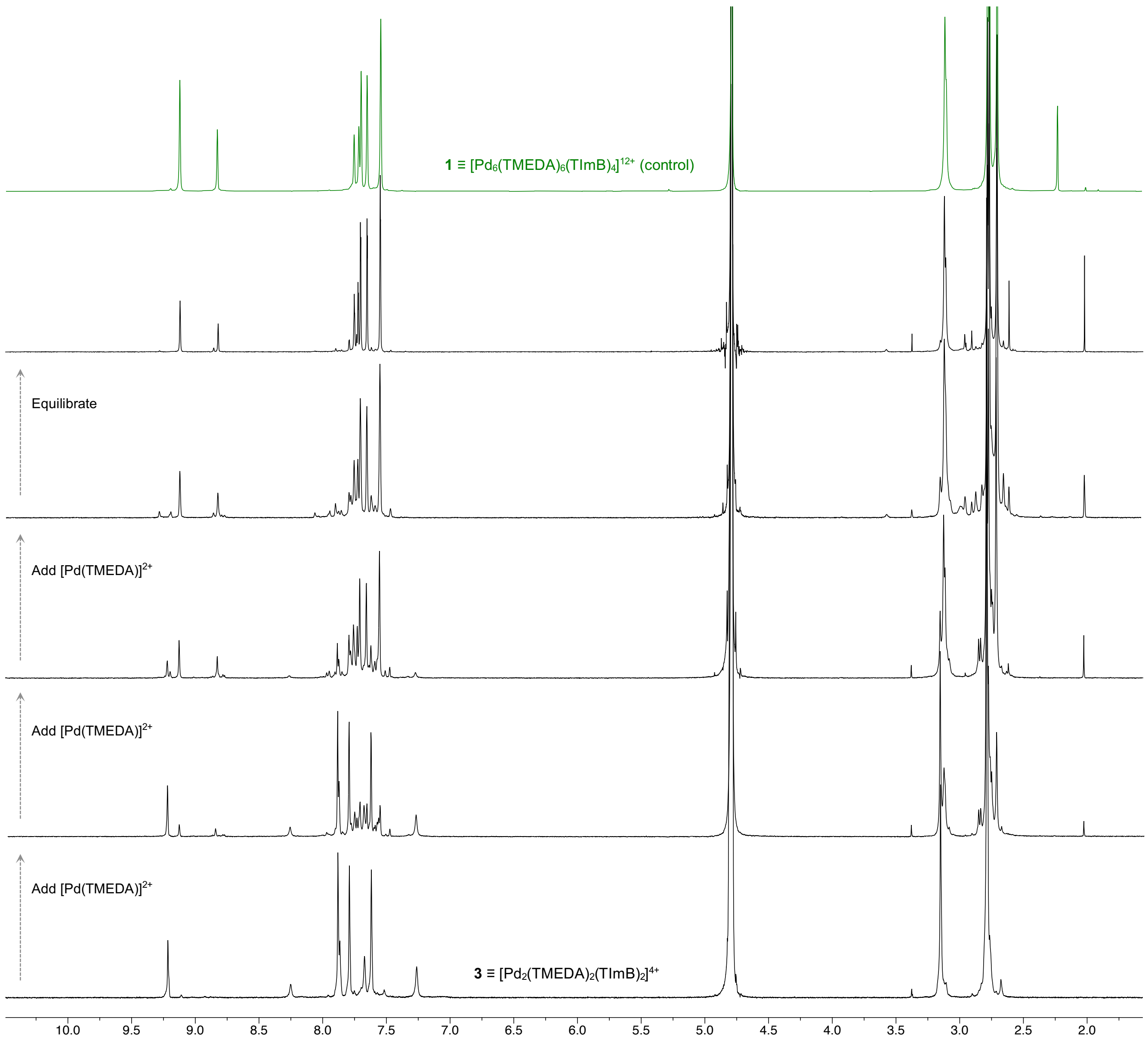


**Supplementary Fig. 28** Stepwise assembly of cage **1** as a confirmation of the identity of **3** (600 MHz, $D_2O$, 300 K). First, species **3** was assembled from a 1:1:1 mixture of $Pd^{2+}$, TImB, and TMEDA, as described on p. 13. Then, **3** was titrated with $[Pd(TMEDA)]^{2+}$, eventually giving rise to a spectrum reminiscent of that of pure **1** (shown in green).

## 3.2. MS characterization

For experimental details, see the Methods section of the main text. HR-MS (ESI$^-$): calcd for $C_{42}H_{56}O_{15}N_{21}Pd_2$ ($[M+5NO_3]^-$): 1306.2332; found: 1306.2345 (*m*/*z*).

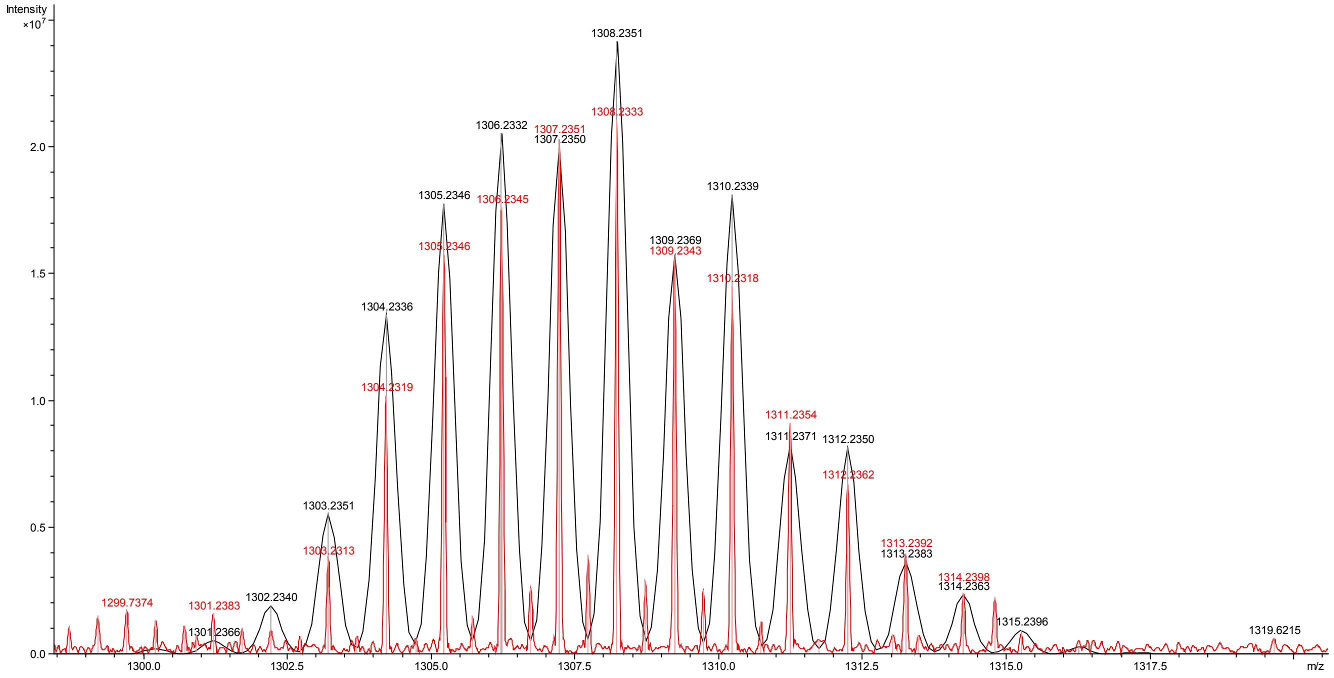


**Supplementary Fig. 29** Simulated (black) and experimental (red) HR-MS spectrum of **3** showing the characteristic isotope pattern of palladium. The corresponding full-range spectrum is shown below.

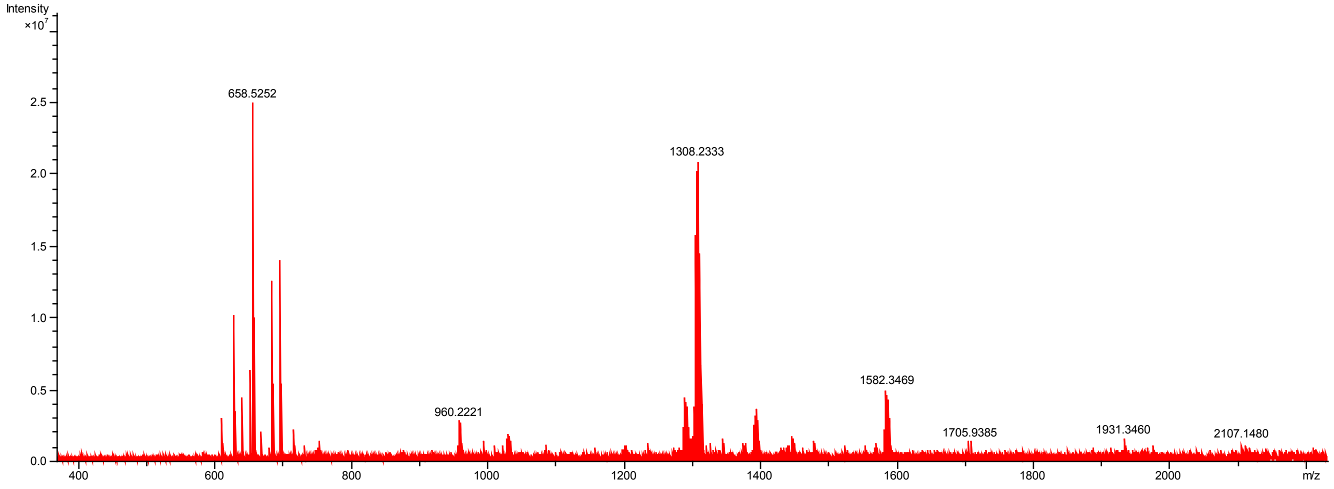


**Supplementary Fig. 30** Full-range HR-MS spectrum of **3**.

## 4. Synthesis and characterization of $\mathbf{4}_2\subset\mathbf{1}$

Inclusion complex $\mathbf{4}_2\subset\mathbf{1}$ was obtained by mixing an aqueous solution of cage **1** (0.5 eq) with resazurin **4** (sodium salt) dissolved in water.

_Note_: Commercial **4** contains a small amount of a radical contamination, which lowers the resolution of NMR spectra. To remove this contamination, **4** was dissolved in a small amount of methanol and the solution was stirred for several minutes under ambient conditions. Next, methanol was removed using a stream of nitrogen and the resulting solid was dried for several minutes under vacuum and dissolved in $D_2O$ for NMR experiments.

### 4.1. NMR spectroscopy characterization

$^1$H NMR (600 MHz, $D_2O$, 330 K): δ (ppm) = 9.22 (s, 8H, $\mathbf{1_4}$), 9.12 (s, 4H, $\mathbf{1_1}$), 7.88 (s, 4H, $\mathbf{1_3}$), 7.77 (s, 12H, $\mathbf{1_7}$+$\mathbf{1_8}$), 7.67 (s, 4H, $\mathbf{1_2}$), 7.57 (s, 8H, $\mathbf{1_5}$), 7.48 (s, 8H, $\mathbf{1_6}$), 5.49 (d, $J$ = 9.2 Hz, 4H, $\mathbf{4_\beta}$), 5.30 (d, $J$ = 8.5 Hz, 4H, $\mathbf{4_\alpha}$), 5.26 (s, 4H, $\mathbf{4_\gamma}$), 3.13 (s, 8H, $\mathbf{1}_{\mathbf{9},\mathrm{ax}}$), 3.05 (s, 16H, $\mathbf{1}_{\mathbf{9},\mathrm{eq}}$), 2.81 (s, 24H, $\mathbf{1}_{\mathbf{10},\mathrm{ax}}$), 2.73 (s, 24H, $\mathbf{1}_{\mathbf{10},\mathrm{eq}}$), 2.56 (s, 24H, $\mathbf{1}_{\mathbf{10},\mathrm{eq}}$).

$^{13}$C NMR (151 MHz, $D_2O$, 330 K): δ (ppm) = 181.1 ($\mathbf{4_\delta}$), 151.5, 138.2, 137.8, 137.6, 137.3 ($\mathbf{1_4}$), 136.7 ($\mathbf{1_4}$), 130.2, 129.9 ($\mathbf{1_2}$), 129.5 ($\mathbf{1_5}$), 125.6 ($\mathbf{4_\beta}$), 121.3, 121.1 ($\mathbf{1_3}$), 120.5 ($\mathbf{1_6}$), 119.2 ($\mathbf{4_\alpha}$), 111.9 ($\mathbf{1_7}$/$\mathbf{1_8}$), 111.3 ($\mathbf{1_7}$/$\mathbf{1_8}$), 102.0 ($\mathbf{4_\gamma}$), 63.14 ($\mathbf{1}_{\mathbf{9},\mathrm{ax}}$), 63.06 ($\mathbf{1}_{\mathbf{9},\mathrm{eq}}$), 50.8 ($\mathbf{1}_{\mathbf{10},\mathrm{ax}}$), 50.7 ($\mathbf{1}_{\mathbf{10},\mathrm{eq}}$), 50.5 ($\mathbf{1}_{\mathbf{10},\mathrm{eq}}$).

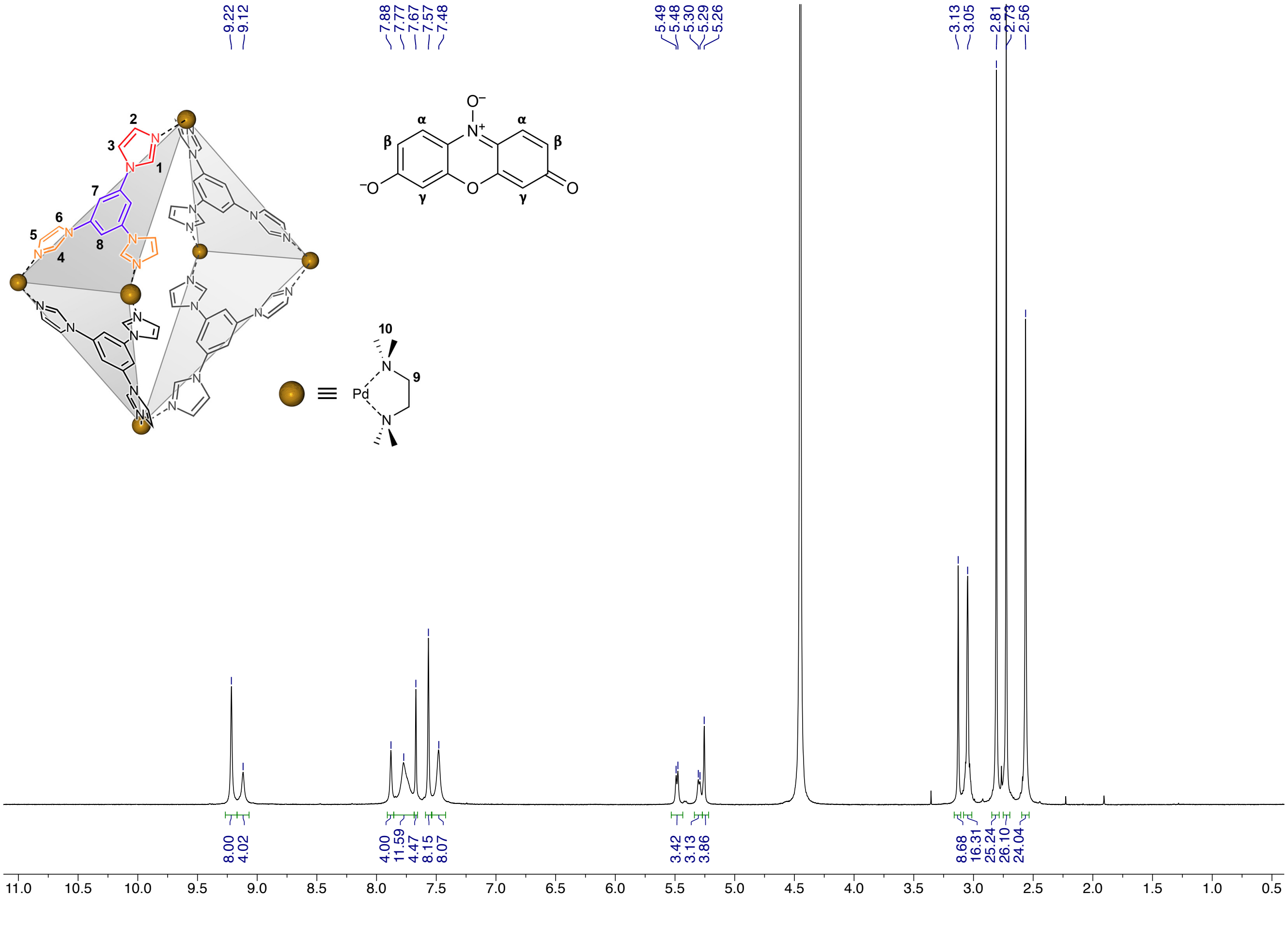


**Supplementary Fig. 31** $^1$H NMR spectrum of $\mathbf{4}_2\subset\mathbf{1}$ (600 MHz, $D_2O$, 330 K).

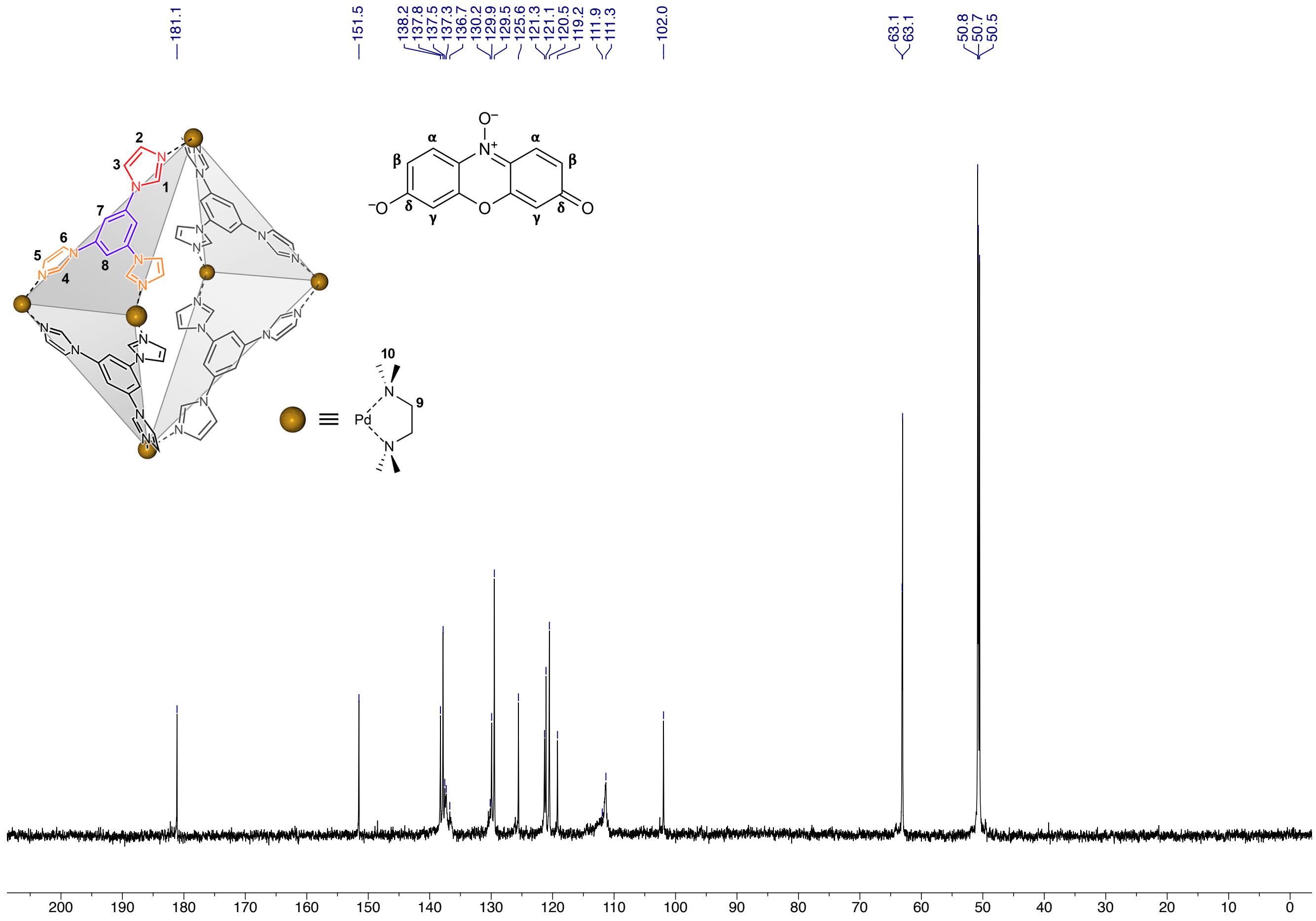


**Supplementary Fig. 32** $^{13}$C NMR spectrum of $\mathbf{4}_2\subset\mathbf{1}$ (151 MHz, $D_2O$, 330 K).

The peak assignment in the NMR spectra of $\mathbf{4}_2\subset\mathbf{1}$ is based on the $^{1}$H, $^{13}$C, and $^{1}$H–$^{13}$C HSQC spectra of **4** (sodium salt) in $D_2O$, which are shown below.

$^{1}$H NMR (600 MHz, $D_2O$, 330 K): δ = 8.02 (d, $J$ = 9.6 Hz, 2H, $\mathbf{4}_{\alpha}$), 6.77 (dd, $J$ = 9.7, 2.3 Hz, 2H, $\mathbf{4}_{\beta}$), 6.44 (d, $J$ = 2.3 Hz, 2H, $\mathbf{4}_{\gamma}$).

$^{13}$C NMR (151 MHz, $D_2O$, 330 K): δ = 181.7 ($\mathbf{4}_{\delta}$), 153.5, 125.6 ($\mathbf{4}_{\beta}$), 124.1, 122.3 ($\mathbf{4}_{\alpha}$), 103.2 ($\mathbf{4}_{\gamma}$).

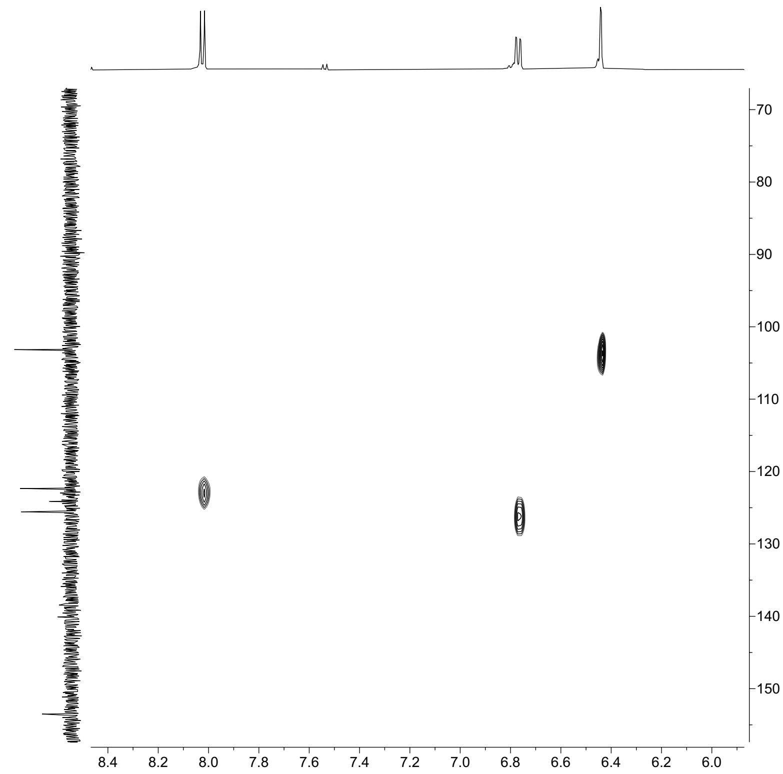


**Supplementary Fig. 33** $^{1}H$–$^{13}C$ HSQC NMR spectrum of **4** ($Na^{+}$ salt) (600 MHz, $D_2O$, 330 K).

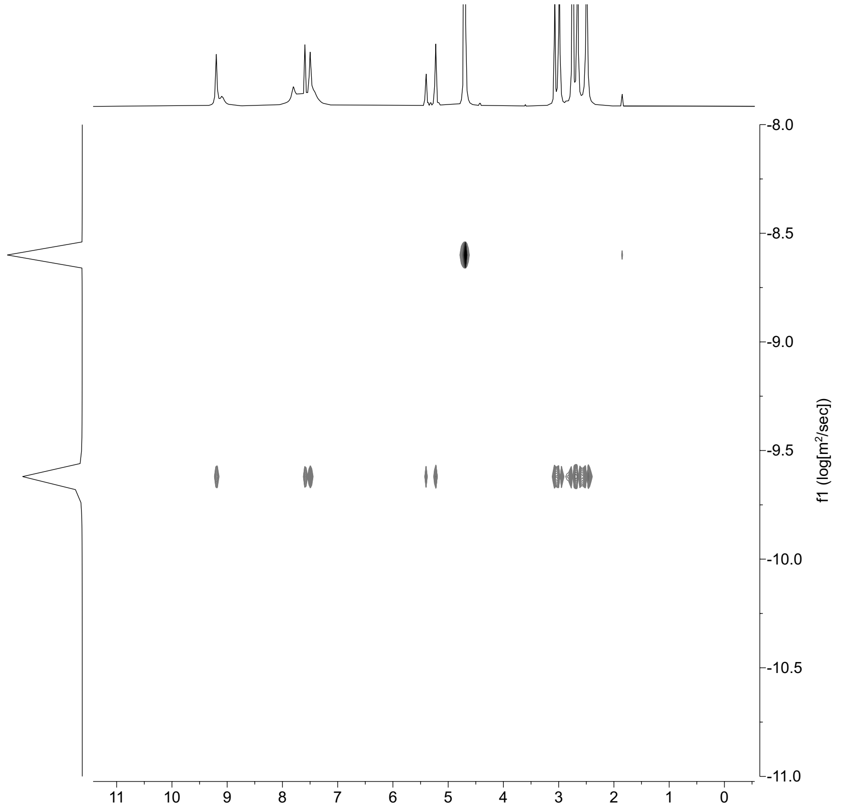


**Supplementary Fig. 34** $^{1}H$ DOSY NMR spectrum of **4**$_2$⊂**1** (600 MHz, $D_2O$, 300 K).

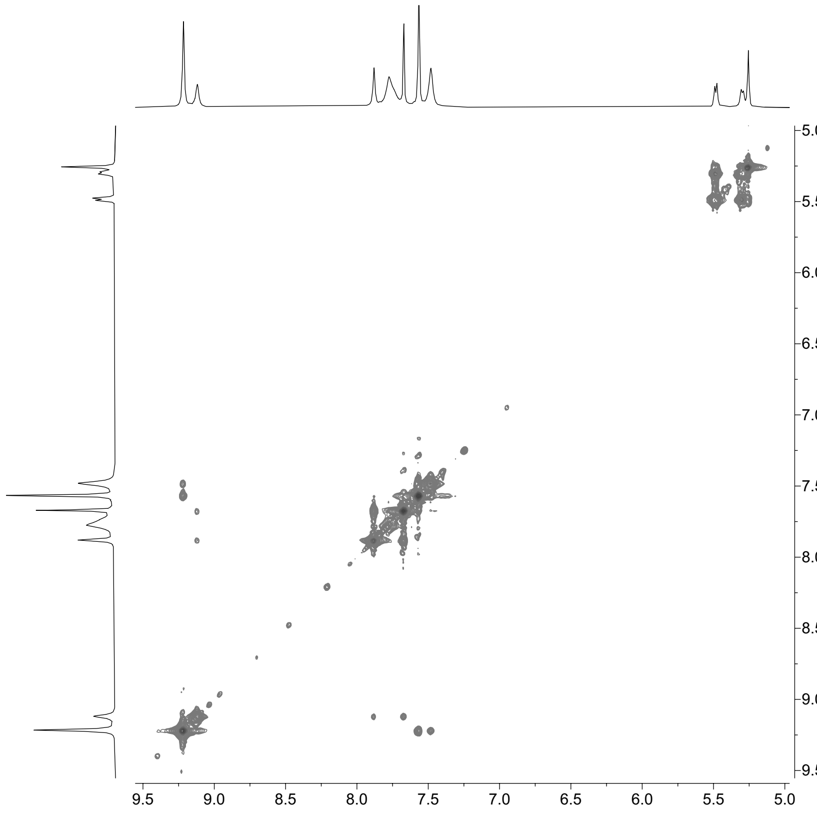


**Supplementary Fig. 35** Partial $^1$H–$^1$H COSY NMR spectrum of $\mathbf{4}_2{\subset}\mathbf{1}$ (600 MHz, $D_2O$, 330 K).

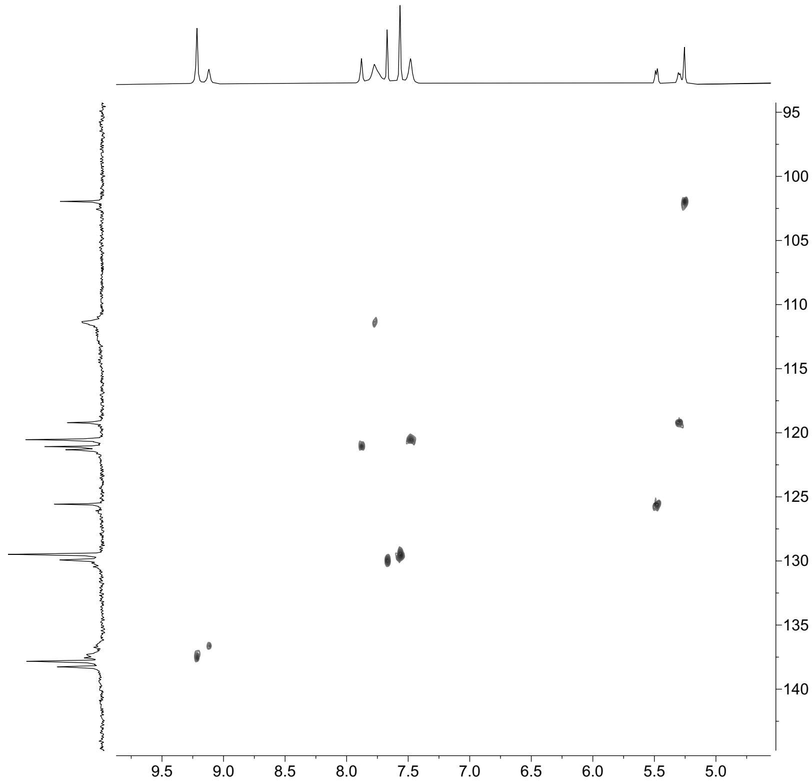


**Supplementary Fig. 36** Partial $^1$H–$^{13}$C HSQC NMR spectrum of $\mathbf{4}_2{\subset}\mathbf{1}$ (600 MHz, $D_2O$, 330 K).

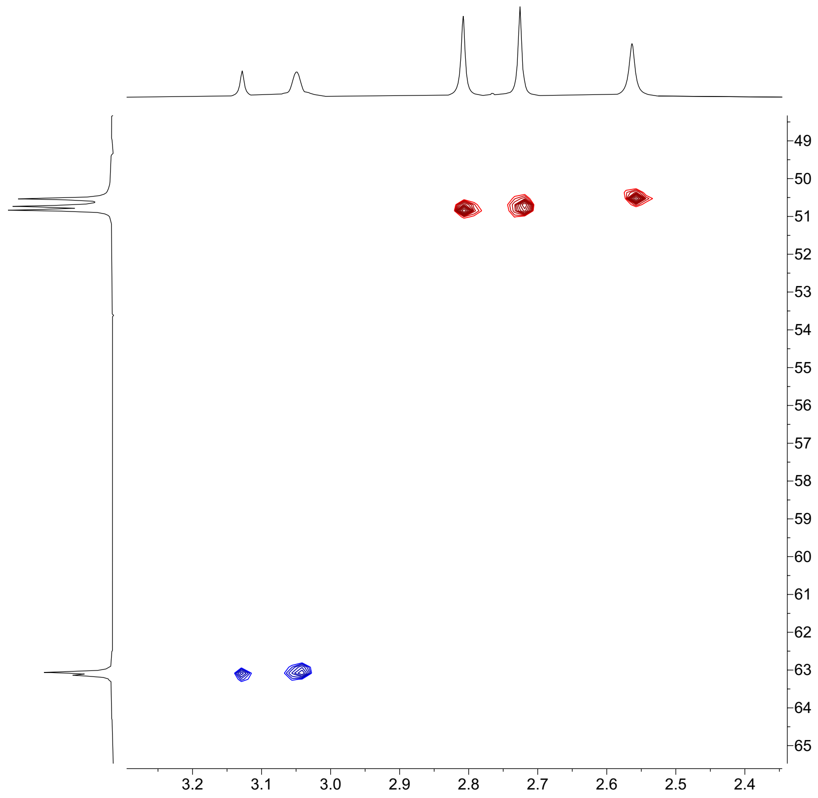


**Supplementary Fig. 37** Partial $^{1}H$–$^{13}C$ HSQC NMR spectrum of **$4_2$⊂1** (600 MHz, $D_2O$, 330 K).

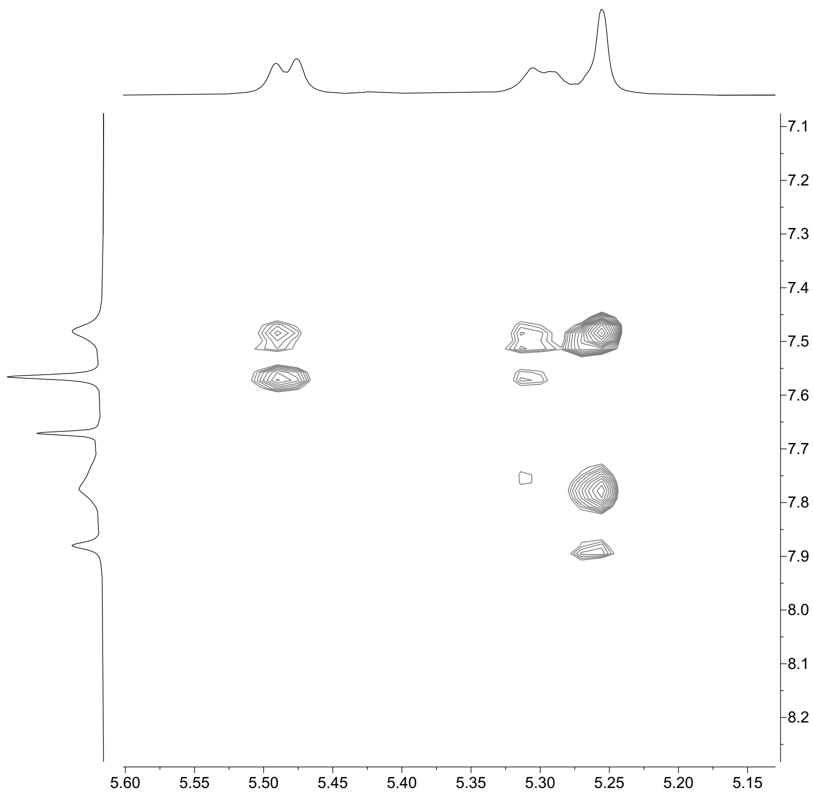


**Supplementary Fig. 38** Partial $^{1}H$–$^{1}H$ NOESY NMR spectrum of **$4_2$⊂1** (600 MHz, $D_2O$, 330 K).

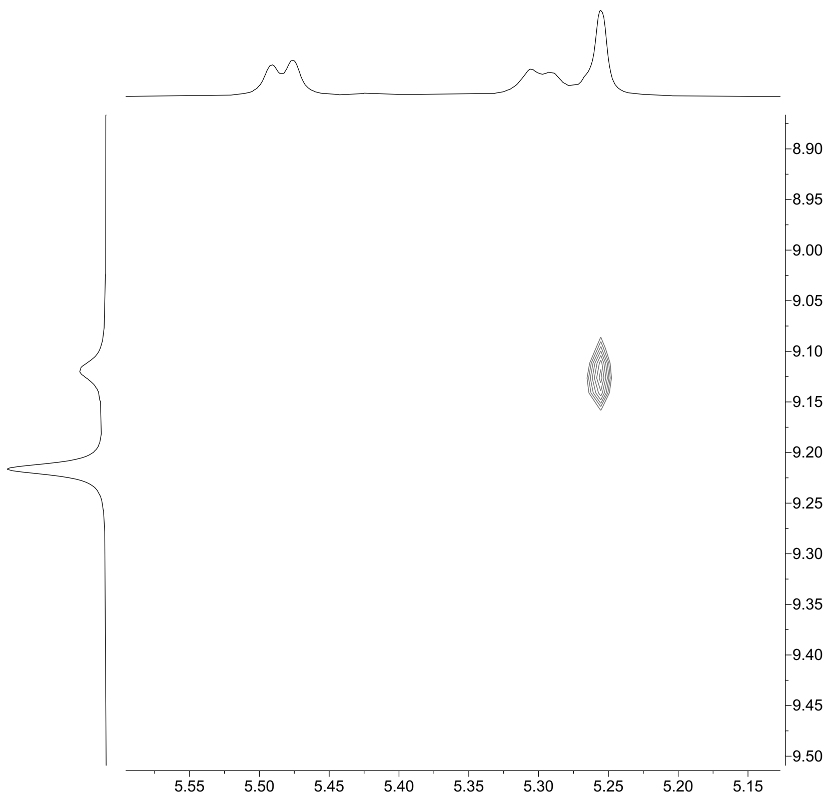


**Supplementary Fig. 39** Partial $^1$H–$^1$H NOESY NMR spectrum of $\mathbf{4}_2\subset\mathbf{1}$ (600 MHz, $D_2O$, 330 K).

To facilitate the interpretation of the 2D NMR data shown above, $^{1}H$ and $^{13}C$ spectra of free **4** are included below.

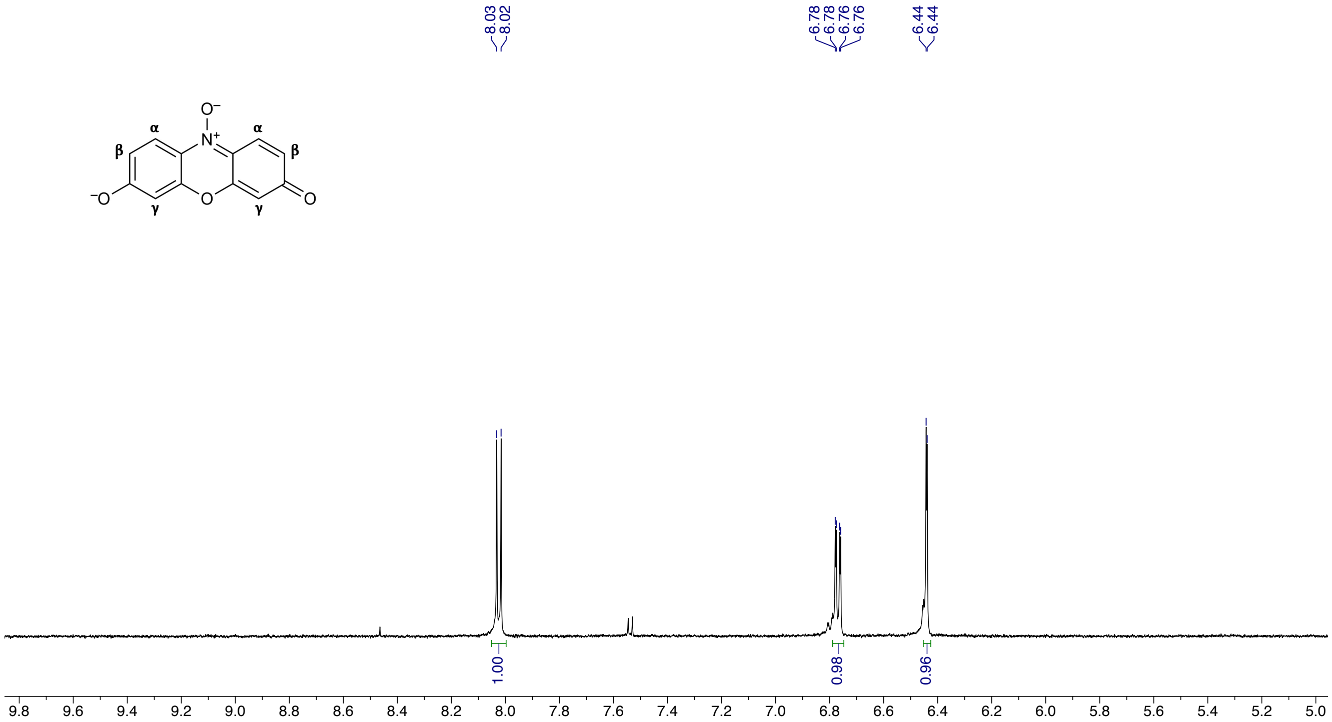


**Supplementary Fig. 40** $^{1}H$ NMR spectrum of **4** ($Na^{+}$ salt) (600 MHz, $D_2O$, 330 K).

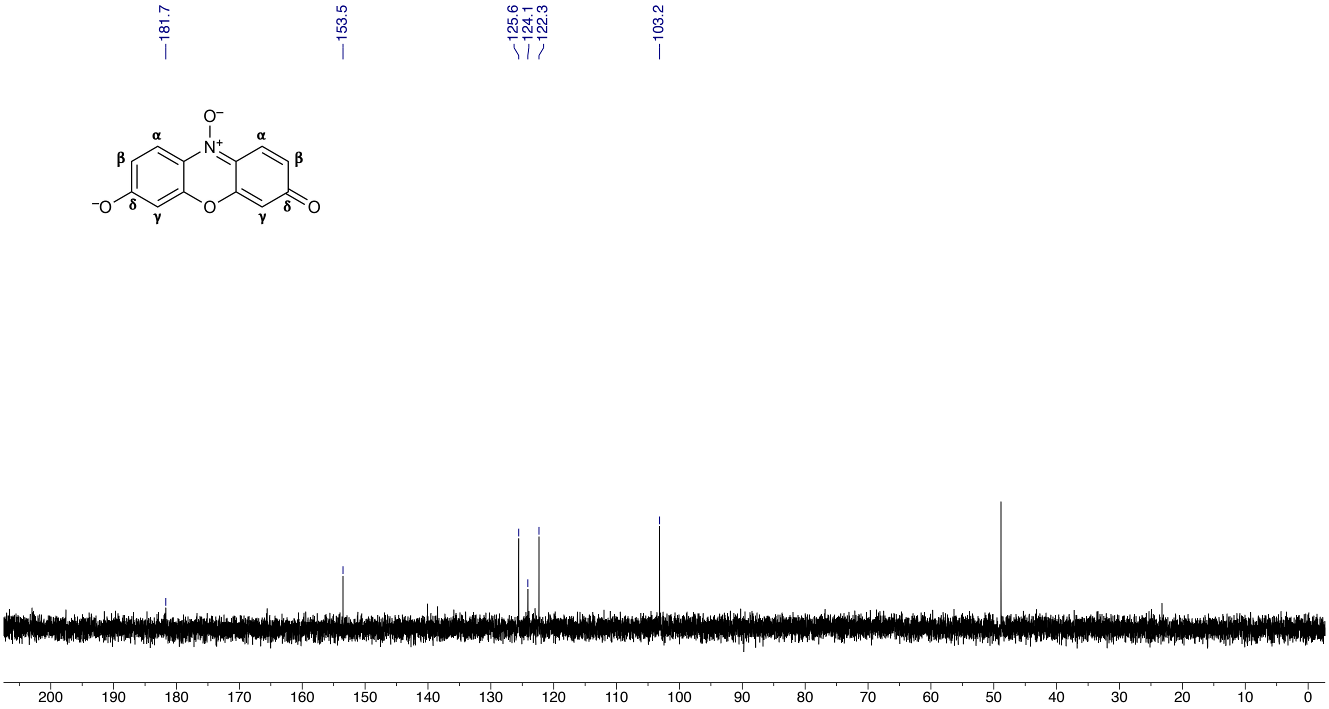


**Supplementary Fig. 41** $^{13}C$ NMR spectrum of **4** ($Na^{+}$ salt) (151 MHz, $D_2O$, 330 K).

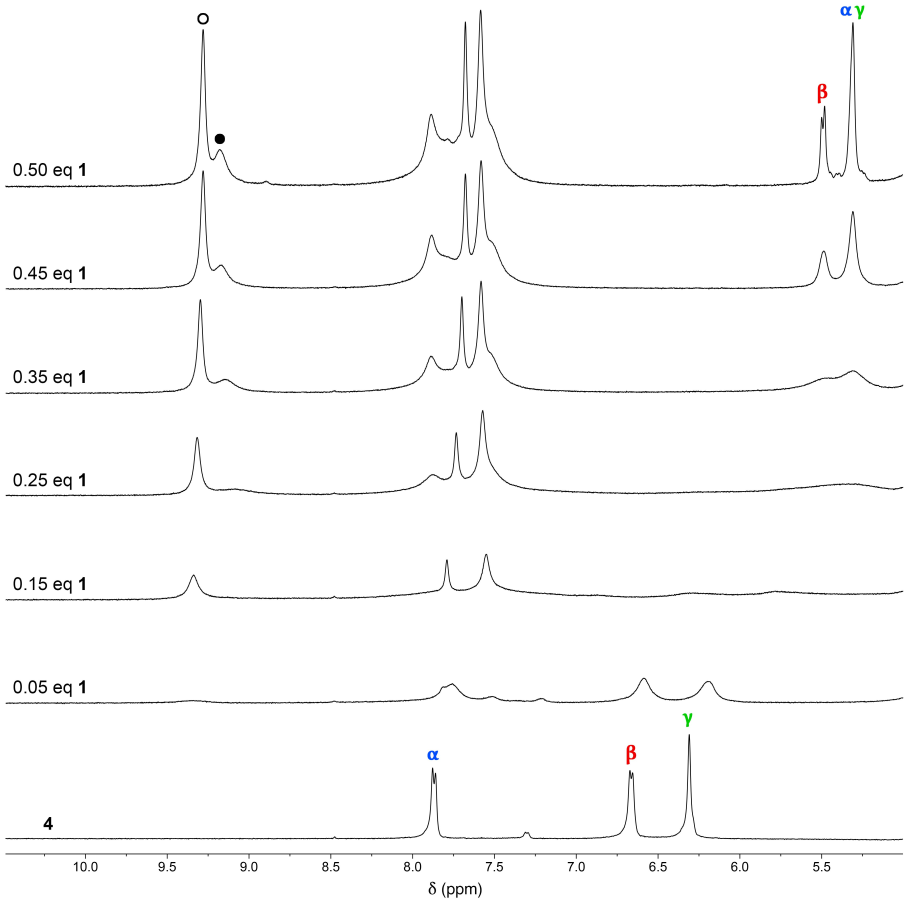


**Supplementary Fig. 42** Partial $^1$H NMR spectra (500 MHz, $D_2O$, 298 K) of **4** in the presence of increasing amounts (up to 0.5 equiv) of cage **1** (compare with Fig. 2a). The empty and solid circles denote the axial and equatorial acidic imidazole protons of **1**, respectively.

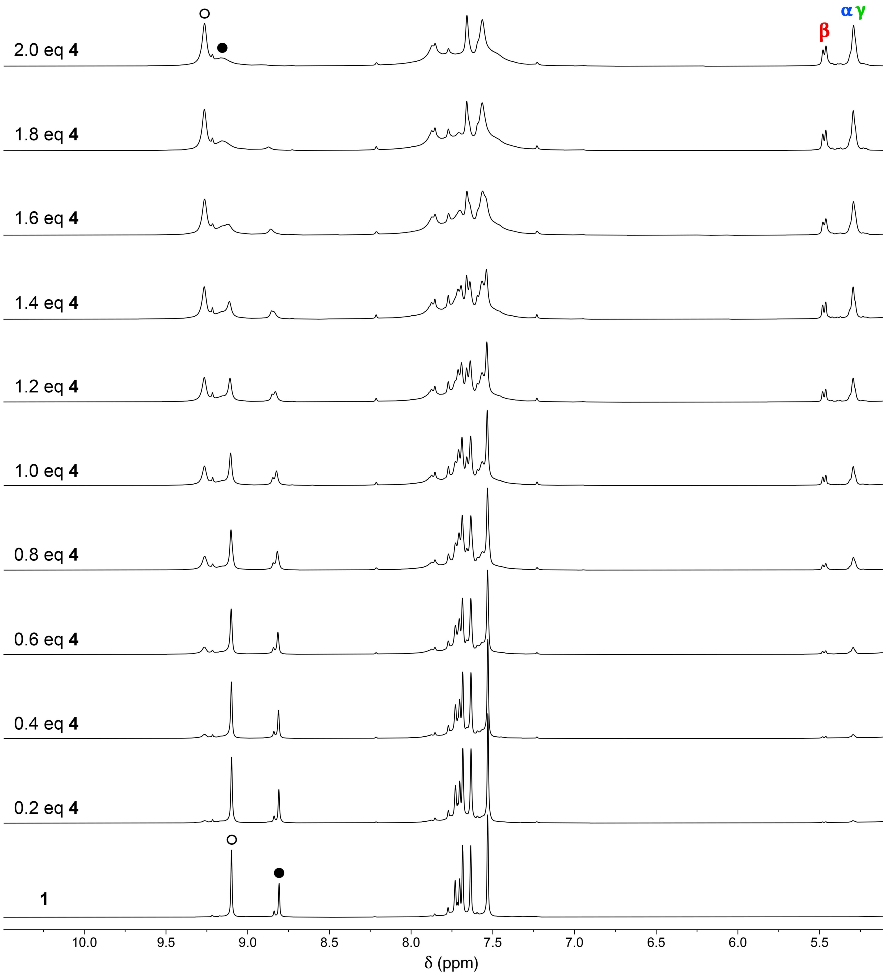


**Supplementary Fig. 43** Partial $^1$H NMR spectra (500 MHz, $D_2O$, 298 K) of **1** in the presence of increasing amounts (up to 2.0 equiv) of **4**. The empty and solid circles denote the axial and equatorial acidic imidazole protons of **1**, respectively.

## 4.2. Solid-state structure characterization

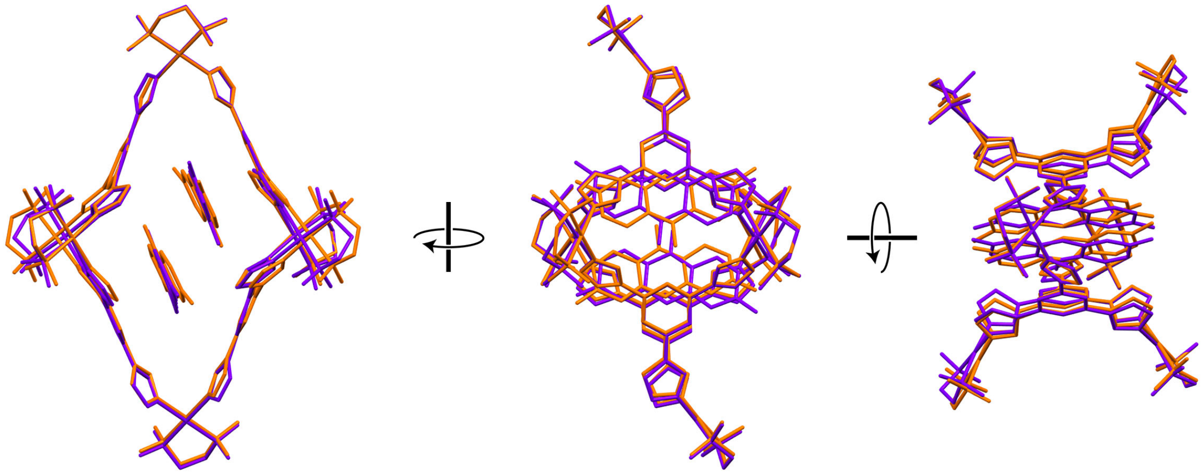

**Supplementary Fig. 44** Overlay of the structures of $\mathbf{4}_2{\subset}\mathbf{1}$ found in the crystals of ($\mathbf{4}_2{\subset}\mathbf{1}$)·**4** (purple) and crystals of $\mathbf{4}_2{\subset}\mathbf{1}$ free of unencapsulated **4** (orange). Hydrogen atoms, unencapsulated **4**, nitrates, and water molecules were omitted for clarity.

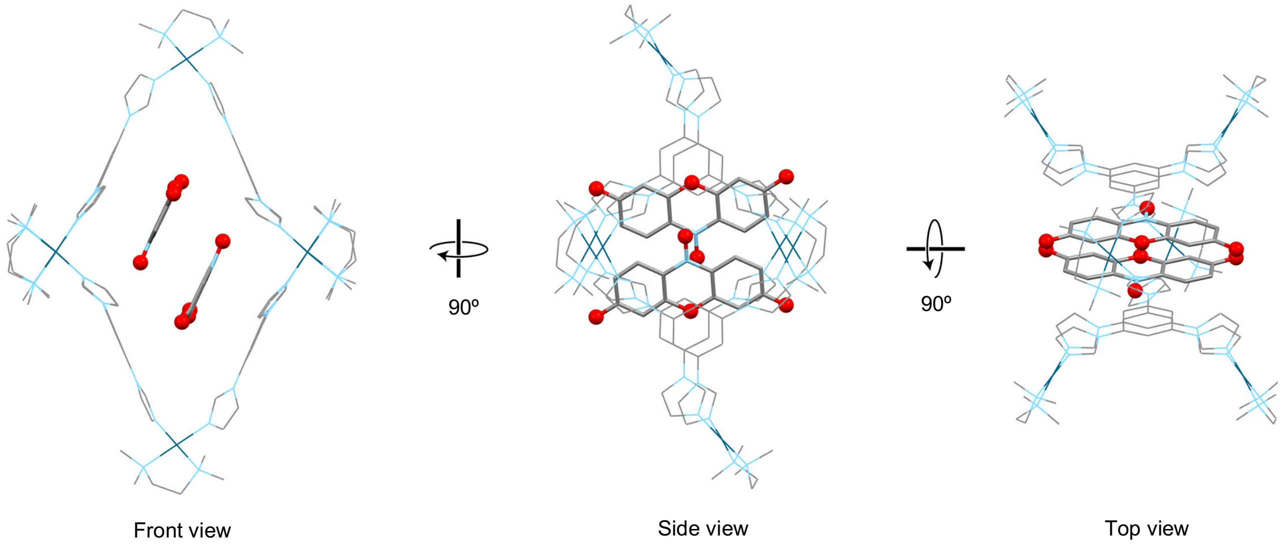


**Supplementary Fig. 45** X-ray structure of $\mathbf{4}_2{\subset}\mathbf{1}$ (here, in the crystal free of unencapsulated **4**) along three different viewing directions. The oxygen atoms are indicated as red spheres to highlight the antiparallel alignment of the two guests. Hydrogens, nitrates, and water molecules were omitted for clarity. Pd, dark-blue; C, gray; N, light-blue.

## 4.3. UV-Vis titration experiments

*Titration of resazurin with cage **1***: Titration experiments were carried out in double-distilled water. Cage **1** (10 mg) was dissolved in water (0.5 mL) and the solution was allowed to equilibrate for one day. 10 mg of **4** (sodium salt) was dissolved in 2 mL of water. 2 µL of the resulting solution was diluted with 1 mL of water and titrated with small aliquots of **1** (3.15 µL), each corresponding to 0.05 equiv of the guest with respect to the cage. The injection rate was 2 min per aliquot; after each injection, a UV-Vis absorption spectrum was recorded. Titration was continued until 1.0 equiv of **1** was added. The resulting UV-Vis absorption spectra are shown in Fig. 5b.

*Titration of cage **1** with resazurin*: Titration experiments were carried out in double-distilled water. Cage **1** (15 mg) was dissolved in water (0.5 mL) and the solution was allowed to equilibrate for one day. 0.65 µL of the resulting solution was diluted with 1 mL of water. A solution of 5 mg of **4** (resazurin sodium salt) in 5 mL of water was prepared. The diluted solution of **1** was titrated with small aliquots of **4** (0.3 µL), each corresponding to 0.2 equiv with respect to **1**. The injection rate was 2 min per aliquot; after each injection, a UV-Vis absorption spectrum was recorded. Titration was continued until 3.0 equiv of **2** were added. The resulting UV-Vis absorption spectra are shown in Supplementary Fig. 46a.

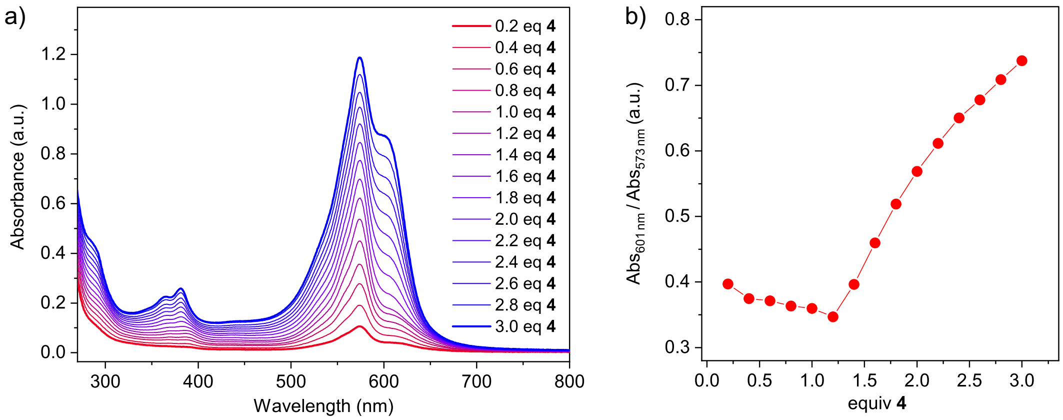


**Supplementary Fig. 46 a)** Evolution of the UV-Vis absorption spectra of cage **1** upon the gradual addition of **4**. **b)** Analysis of the UV-Vis spectra. High absorbance at 601 nm indicates the presence of free **4**.

We repeated the titration of the dilute solution of **1** with **4** until 2.0 equiv of **4** were added (at this point of titration, UV-Vis absorption spectra consistently showed significant amounts of unencapsulated **4**; Supplementary Fig. 47a). We then let the solution equilibrate for 19 h and remeasured the spectrum, obtaining the green spectrum in Supplementary Fig. 47a. Analysis of the spectra (Supplementary Fig. 47b) showed that the amount of free **4** decreases significantly over 19 h at the expense of encapsulated **4**, which we attribute to the ability of **4** to template the formation of the cage.

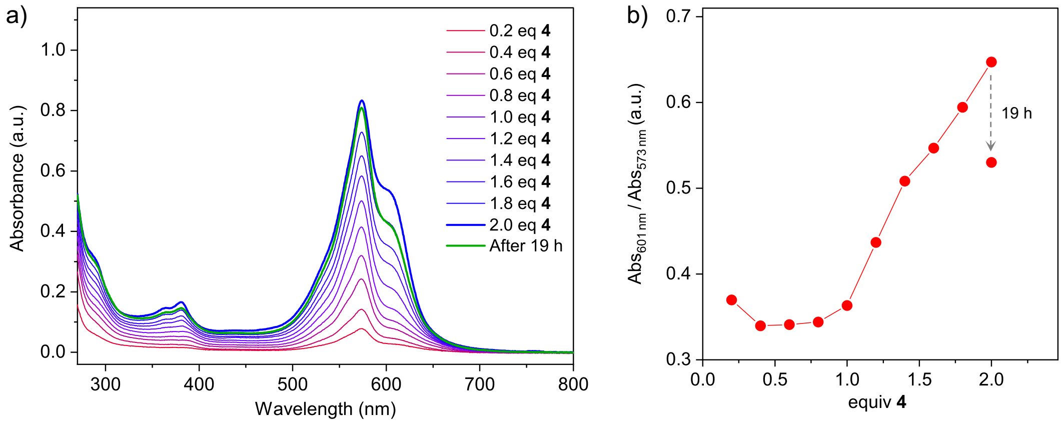


**Supplementary Fig. 47** Resazurin-templated reassembly of cage **1**. **a)** Evolution of the UV-Vis absorption spectra of cage **1** upon the gradual addition of **4**. Once 2.0 equiv of **4** were added (thick blue line), the solution was allowed to equilibrate for 19 h, resulting in the spectrum shown in green. **b)** Analysis of the UV-Vis spectra. High absorbance at 601 nm indicates the presence of free **4**.

## 4.4. Fluorescence titration experiments

*Titration of resazurin with cage **1***: Titration experiments were carried out in double-distilled water. Cage **1** (10 mg) was dissolved in water (0.5 mL) and the solution was allowed to equilibrate for one day. 10 mg of **4** (sodium salt) was dissolved in 2 mL of water. 2 µL of the resulting solution were diluted with 1 mL of water and titrated with small aliquots of **1** (3.15 µL), each corresponding to 0.05 equiv of the guest with respect to the cage. The injection rate was 2 min per aliquot; after each injection, a fluorescence spectrum was recorded (excitation wavelength = 530 nm). Titration was continued until 1.0 equiv of **1** was added. The resulting fluorescence spectra are shown in Supplementary Fig. 48a.

Note: In these experiments, we followed the relatively weak emission band at ~630 nm, which originates from **4**. The main emission band in the fluorescence spectra of **4** ($\lambda_{max}$ = 584 nm) originates from a small amount of the highly fluorescent **2**; this small amount of **2** is difficult to remove.[2] To estimate the amount of **2** in **4**, we prepared solutions of **4** (containing a residual amount of **2**) and pure **2** at the same concentration and found that the intensity of the $\lambda_{max}$ = 584 nm emission band was approximately 19 times lower in **4** than in pure **2**. This result indicates that **4** contains ~5% of **2**, which is hardly visible in the UV-Vis absorption spectra, but obscures the emission spectra. This level of residual **2** in **4** can also be observed in the NMR spectra of **4**; see, e.g., Fig. 5a.

*Titration of cage **1** with resazurin*: Titration experiments were carried out in double-distilled water. Cage **1** (15 mg) was dissolved in water (0.5 mL) and the solution was allowed to equilibrate for one day. 0.65 µL of the resulting solution was diluted with 1 mL of water. A solution of 5 mg of **4** (sodium salt) in 5 mL of water was prepared. The diluted solution of **1** was titrated with small aliquots of **4** (0.3 µL), each corresponding to 0.2 equiv with respect to **1**. The injection rate was 2 min per aliquot; after each injection, a fluorescence spectrum was recorded (excitation wavelength = 530 nm). Titration was continued until 2.0 equiv of **4** were added. The resulting fluorescence spectra are shown in Supplementary Fig. 48c.

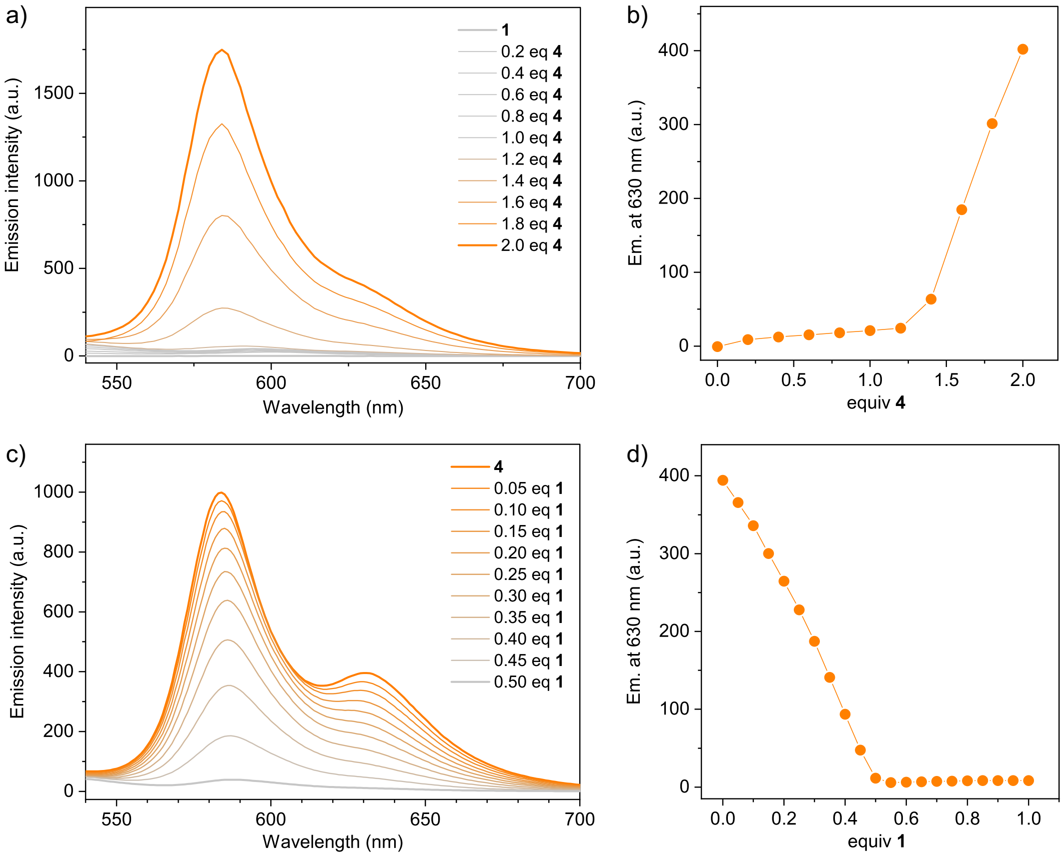


**Supplementary Fig. 48 a)** Evolution of fluorescence spectra (excitation wavelength = 530 nm) of **1** upon the gradual addition of **4**. **b)** Analysis of the spectra shown in a). The rapid increase in fluorescence at ~1.4 equiv of **4** indicates that all the existing cages were filled. **c)** Evolution of fluorescence spectra (excitation wavelength = 530 nm) of **4** upon adding a concentrated solution of cage **1**. **d)** Analysis of the spectra shown in c). The emission of 630 nm is quenched once 0.5 equiv was added, indicating that all the dyes were encapsulated.

## 4.5. Isothermal titration calorimetry (ITC)

For the description of the experiments, see the Methods section.

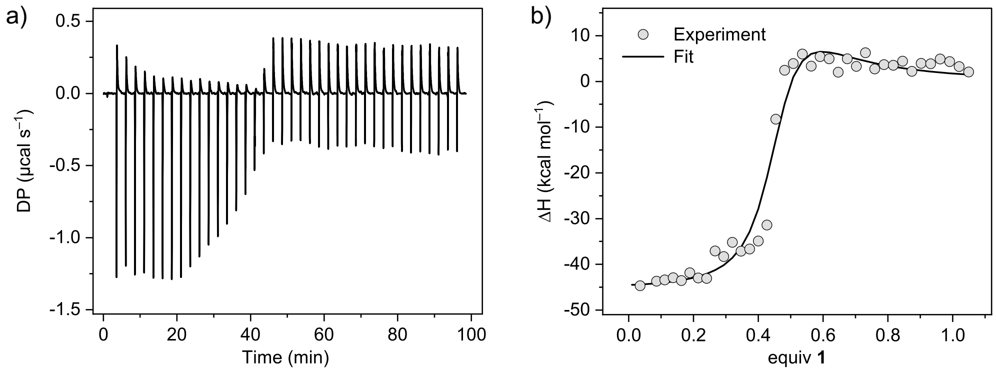


**Supplementary Fig. 49 a)** A representative plot of differential power (DP) vs. time for the titration of **4** (0.44 mM) with cage **1** (2.34 mM). **b)** The resulting plot of ΔH vs. the amount of **1** added, fitted to a 2:1 binding model.

## 4.6. VT-NMR spectroscopy

To ensure elevating temperature improves the quality of the NMR spectra without affecting the inclusion complexes, we recorded $^1$H NMR spectra of the $\mathbf{4}_2\subset\mathbf{1}$ at several different temperatures. As Supplementary Fig. 50 shows, raising the temperature from 10 ºC to 60 ºC gradually sharpened the signals and a high-quality spectrum could be obtained at 60 ºC. Importantly, we note that the chemical shifts of guest's protons did not change – e.g., the most distinct peak due to $\mathbf{4}_\gamma$ appeared at 5.29 ppm, 5.30 ppm, 5.30 ppm, and 5.28 ppm at 283 K, 298 K, 313 K, and 333 K, respectively; in comparison, the chemical shift of free $\mathbf{4}_\gamma$ = 6.40 ppm (Supplementary Fig. 40). Partial release of the guest at an elevated temperature would be accompanied by an appearance of an additional signal at 6.40 ppm and/or a downfield shift of the ~5.30 ppm signal (in case of a relatively slow exchange between free and encapsulated **4**), none of which was observed, indicating that the inclusion complex remains largely intact even at 333 K.

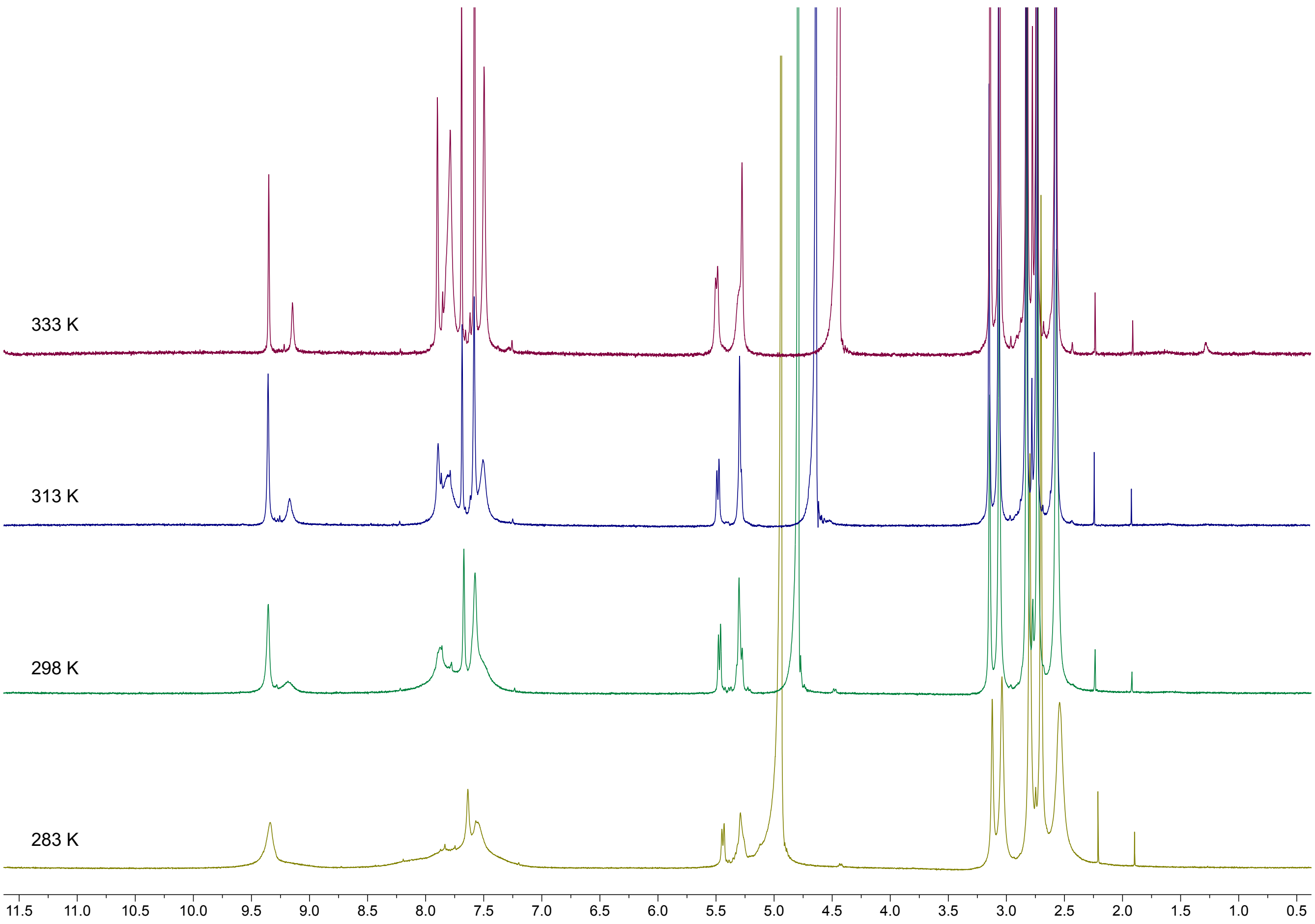


**Supplementary Fig. 50** $^1$H NMR spectra (500 MHz, $D_2O$) of $\mathbf{4}_2\subset\mathbf{1}$ acquired at different temperatures.

## 5. Redox switching of free and encapsulated **2** and **4**

Citrate-protected gold nanoparticles (NPs) were synthesized as described in the Methods section.

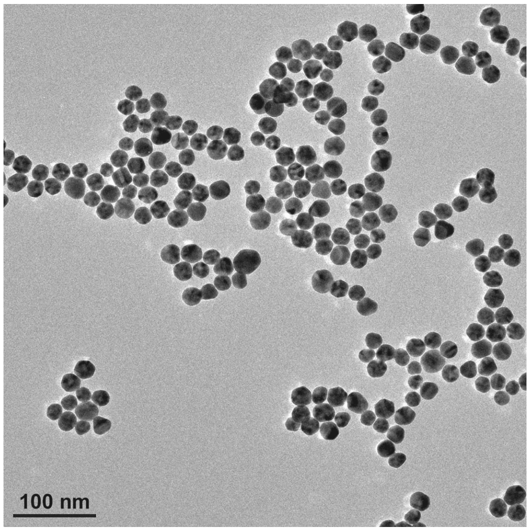


**Supplementary Fig. 51** Representative TEM image of 21 nm citrate-capped gold NPs.

### 5.1. Control experiments

First, we established a system that enables the facile reduction of **4** into **2** under visible light irradiation. To this end, we tested TMEDA as a potential reducing agent for **4**, in the presence of citrate-capped gold NPs and green light. This system was inspired by previous literature reports,[2,3] which described the reduction of **4** by $NH_2OH$ in the presence of gold NPs and a green light source, typically a laser. Interestingly, we found that the reduction proceeded smoothly with 4 equiv of TMEDA, a relatively low-intensity green light-emitting diode (LED), and a very small amount of gold NPs ($2.25 \cdot 10^{-8}$ equiv of particles with respect to **4**). Supplementary Fig. 52 shows the behavior of the system (in the absence of cage **1**). Initially, the vial is blue due to **4** (the NPs are too dilute to contribute to the color of the sample). After 131 s of green light irradiation, orange fluorescence (excited by green light) can be seen, indicating the formation of the highly fluorescent **2**. After 338 s of irradiation, the fluorescence largely disappears, which can be attributed to further reduction of **2** into the colorless, non-fluorescent **5**. Inspection of the sample under ambient light (348 s) revealed that **4** at the bottom of the vial was not reduced, probably due to incomplete mixing of the reagents. Note the thin pink layers of **2** at the water–air interface and at the interface between the **4**-rich and the **5**-rich layers. The **4**-rich layer can be attributed to the rapid reoxidation of **5** to **2** with atmospheric oxygen; the **5**-rich layer is likely due to a synproportionation reaction, **4** + **5** → **2** + **2** + $H_2O$. Indeed, when the vial was shaken, a solution of **2** was obtained (388 s; note that the reduction of **4** into **2** is, under these conditions, irreversible, with $O_2$ only capable of oxidizing **5** into **2**). At the beginning of the second cycle of irradiation, the orange fluorescence of **2** can be seen (429 s). The fluorescence disappears as **2** is reduced to **5** (566 s). Finally, the vial was left undisturbed under ambient light; the slow diffusion of oxygen gradually regenerates the pink **2** (966 s).

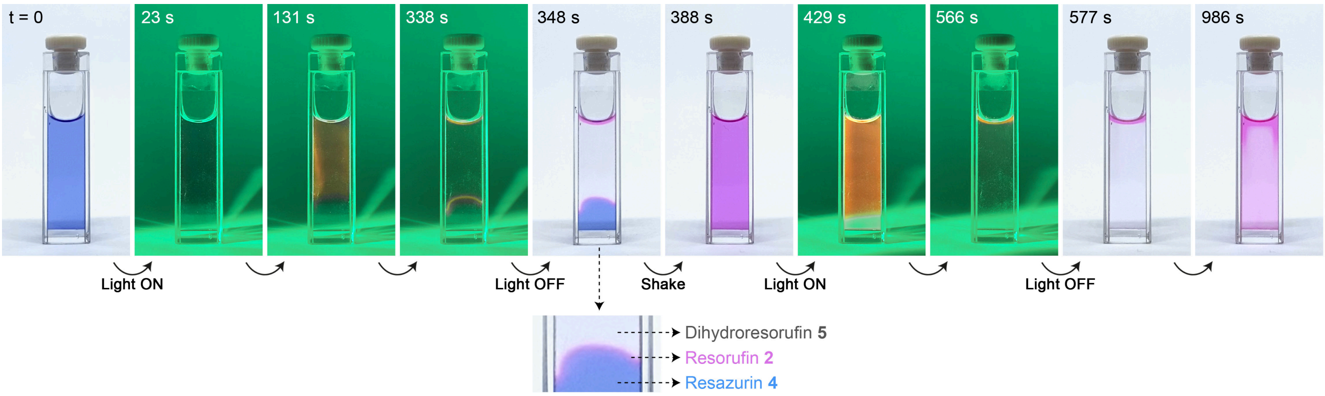


**Supplementary Fig. 52** Visual changes accompanying the NP-catalyzed reduction of resazurin **4** to resorufin **2** to dihydroresorufin **5**, using TMEDA as the reducing agent (example 1).

The first reduction in Supplementary Fig. 52 was slow, most likely due to the presence of oxygen in the initial solution. We then the experiment with a degassed (and more concentrated) solution of **4** (Supplementary Fig. 53). A complete reduction to **2** was observed within ~10 s. The orange-fluorescent solution seen at $t = 16$ s became non-fluorescent colorless as the irradiation was continued for another ~90 s. Diffusion of oxygen induced reoxidation to **2** (405 s).

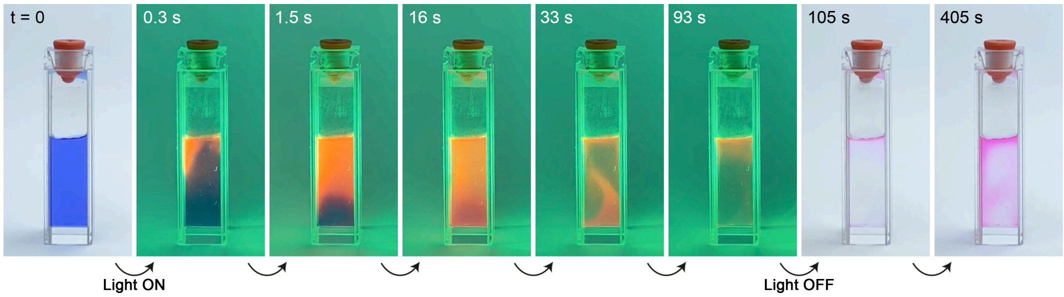


**Supplementary Fig. 53** Visual changes accompanying the NP-catalyzed reduction of resazurin **4** to resorufin **2** to dihydroresorufin **5**, using TMEDA as the reducing agent (example 2).

To better understand the reaction, we repeated it in the presence of increasingly smaller amounts of TMEDA. We found that a facile reduction of **4** into **2** proceeded in the presence of ~0.5 equiv of TMEDA. However, with ~0.3 equiv of TMEDA, only ~60% of **4** was rapidly reduced to **2**; the residual amount of **4** underwent a slow photo-decomposition reaction. Then, we repeated the reaction with ~0.3 equiv TMEDA and as soon as the formation of **2** stopped, we added an additional ~0.2 equiv of TMEDA and continued irradiation with green light, achieving a complete reduction of **4** into **2**. Based on these experiments, we concluded that the reaction occurs through a formal oxygen transfer from **4** to TMEDA to afford TMEDA dioxide.[4-6]

We also investigated the applicability of other tertiary amines, namely, triethylamine (TEA) and diisopropyl-ethylamine (DIPEA), for the NP-catalyzed reduction of **4** (Supplementary Fig. 54). Using TEA (4 equiv) as the reducing agent, under otherwise identical conditions (40 nmol **4**, 4 equiv amine, $2.25 \cdot 10^{-8}$ equiv NPs, green LED), the reduction proceeded significantly slower than with TMEDA; nevertheless, a complete conversion of **4** into **2** could be observed within ~3 min (Supplementary Fig. 54a) (compare with ~10 s for TMEDA; note, however, that TMEDA contains two tertiary amine groups). With DIPEA (4 equiv), a further decrease in the reaction kinetics was observed (Supplementary Fig. 54b). Owing to the relatively long irradiation time, photobleaching of **4** and/or **2** effectively competed with the reduction; consequently, the absorbance at ~570 nm did not reach the expected value (compare with Supplementary Fig. 54a).

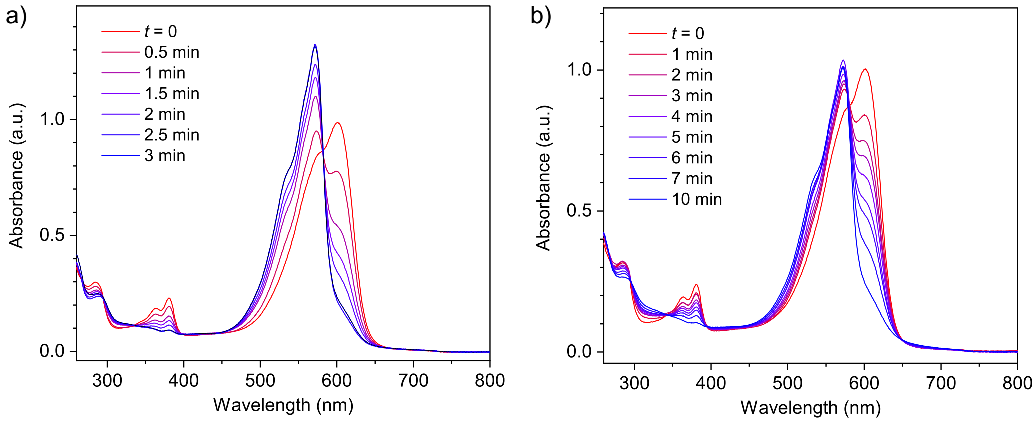


**Supplementary Fig. 54** Changes in the UV-Vis absorption spectra of solutions of **4** containing $2.25 \cdot 10^{-8}$ equiv of Au NPs and 4 equiv of **a)** TEA and **b)** DIPEA under green light irradiation.

To confirm that all three elements of the system (i.e., TMEDA, Au NPs, and green light) are required to efficiently reduce **4**, we performed a series of control experiments, in which one or more of these components were missing. These experiments are described in the main text of the manuscript; the results are shown below.

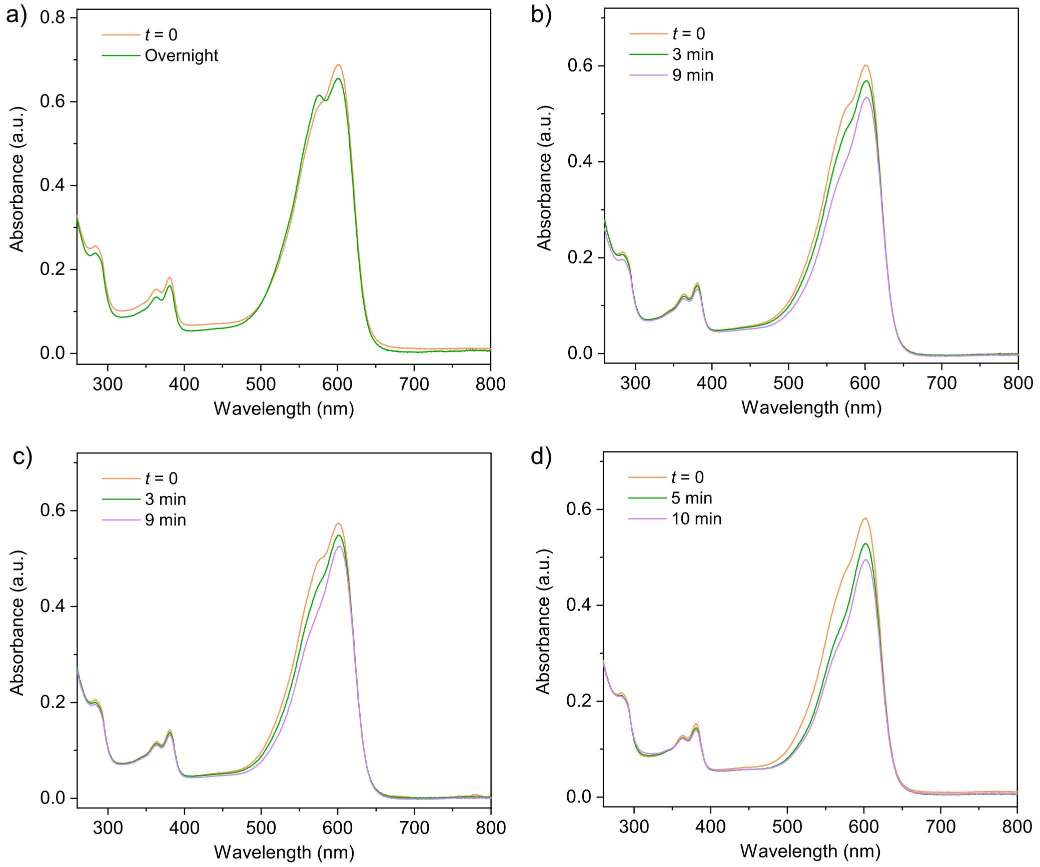


**Supplementary Fig. 55** Changes in the UV-Vis absorption spectra of aqueous solutions of **4** exposed to **a)** 4 equiv of TMEDA (no NPs; no light), **b)** green light (no TMEDA, no NPs), **c)** 4 equiv of TMEDA and green light (no NPs), and **d)** $2.25 \cdot 10^{-8}$ equiv of Au NPs and green light (no TMEDA).

As the final control experiment, we subjected cage **1** (20 nmol) to our standard reducing conditions (160 nmol TMEDA, $9.0\cdot10^{-7}$ nmol NPs, green light). No changes in the UV-Vis spectra were found after 15 min of irradiation (Supplementary Fig. 56), suggesting that the cage remains intact under these conditions (i.e., no reduction to $Pd^0$ was observed).

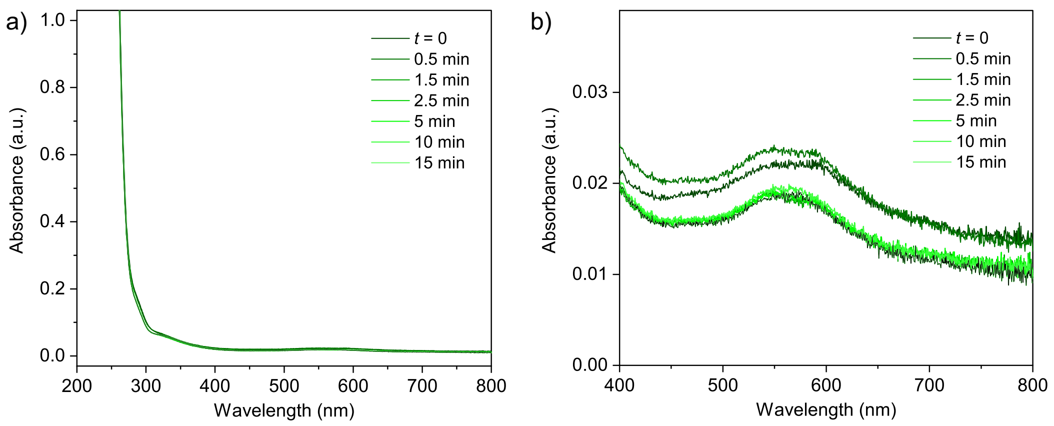


**Supplementary Fig. 56** UV-Vis absorption spectrum of cage **1** in the presence of 8 equiv of TMEDA and $4.5\cdot10^{-8}$ equiv of Au NPs before ($t$ = 0) and after green light irradiation. Panel b) shows the magnified view in the 400–800 nm range, where the band at ~550 nm originates due to the catalytic amount of Au NPs.

## 5.2. Reduction in the presence of extra cage

To determine whether the presence of extra cage in solution influences on the kinetics of the reduction of **4**, we repeated the reaction under our standard reducing conditions (4 equiv TMEDA, $2.25 \cdot 10^{-8}$ equiv Au NPs, green LED) in the presence of various amounts of empty cage. Supplementary Fig. 57 shows the decay of **4** (blue markers) and the production of **2** (red markers) in the absence of free cage (i.e., $\mathbf{4_2{\subset}1}$; Supplementary Fig. 57a) and with an additional 1, 2, and 5 equiv of **1** (Supplementary Fig. 57b, c, and d, respectively). The concentrations of **2** and **4** at different times were calculated independently based on the UV-Vis absorption spectra, after having determined the extinction coefficients of $\mathbf{2_2{\subset}1}$ and $\mathbf{4_2{\subset}1}$ (based on the spectra of pure $\mathbf{2_2{\subset}1}$ and $\mathbf{4_2{\subset}1}$ at known concentrations). In all cases, the reaction was found to follow the apparent first-order kinetics, with the reaction rates inversely proportional to the concentration of free **1** (Supplementary Fig. 57e).

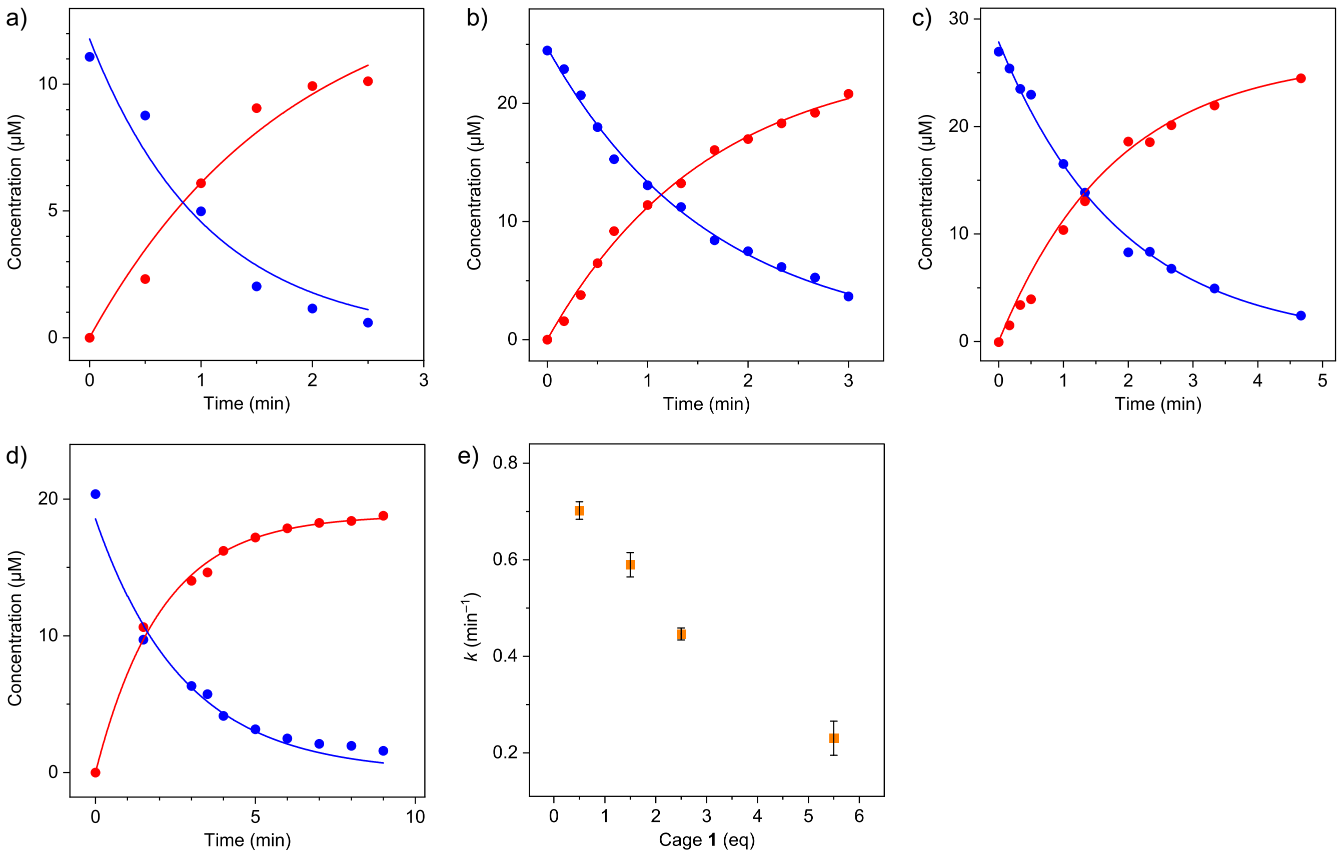


**Supplementary Fig. 57** Concentration changes of **4** and **2** during the NP-catalyzed reduction of $\mathbf{4_2{\subset}1}$ using TMEDA in the presence of different amounts of extra cage **1**: **a)** no free **1**, **b)** 1 equiv **1**, **c)** 2 equiv **1**, and **d)** 5 equiv **1**. **e)** The apparent first-order reaction constants *k* as a function of the total amount of **1** in the system. Each reaction was performed twice and the average values of *k* are reported.

## 6. Synthesis and characterization of **6**$_2$⊂**1**

Inclusion complex **6**$_2$⊂**1** was obtained by stirring an aqueous solution of cage **1** above an excess of solid **6** overnight.

### 6.1. NMR spectroscopy

$^1$H NMR (600 MHz, $D_2O$, 320 K): δ (ppm) = 9.11 (s, 4H, **1**$_1$), 9.02 (s, 8H, **1**$_4$), 7.74 (s, 4H, **1**$_3$), 7.68 (s, 4H, **1**$_2$), 7.61 (s, 8H, **1**$_7$), 7.55 (s, 8H, **1**$_5$), 7.45 (s, 12H, **1**$_6$+**1**$_8$), 5.50 (s, 4H, **6**), 5.44 (s, 4H, **6**), 4.88 (s, 4H, **6**), 4.83 (s, 4H, **6**), 3.10 (s, 8H, **1**$_{9,ax}$), 3.06 (s, 16H, **1**$_{9,eq}$), 2.74 (s, 24H, **1**$_{10,ax}$), 2.71 (s, 24H, **1**$_{10,eq}$), 2.62 (s, 24H, **1**$_{10,eq}$).

$^{13}$C NMR (151 MHz, $D_2O$, 320 K): δ (ppm) = 141.6, 138.3, 138.0, 137.4 (**1**$_4$), 137.1 (**1**$_1$), 137.0, 130.5, 129.6 (**1**$_2$), 129.2 (**1**$_5$), 122.9 (**6**), 121.1, 121.0 (**1**$_3$), 120.8 (**1**$_6$), 119.8 (**6**), 113.6 (**1**$_3$+**6**), 112.3 (**1**$_7$), 112.0 (**6**), 63.0 (**1**$_{9,ax}$), 62.9 (**1**$_{9,eq}$), 50.62 (**1**$_{10,ax}$), 50.59 (**1**$_{10,eq}$), 50.3 (**1**$_{10,eq}$).

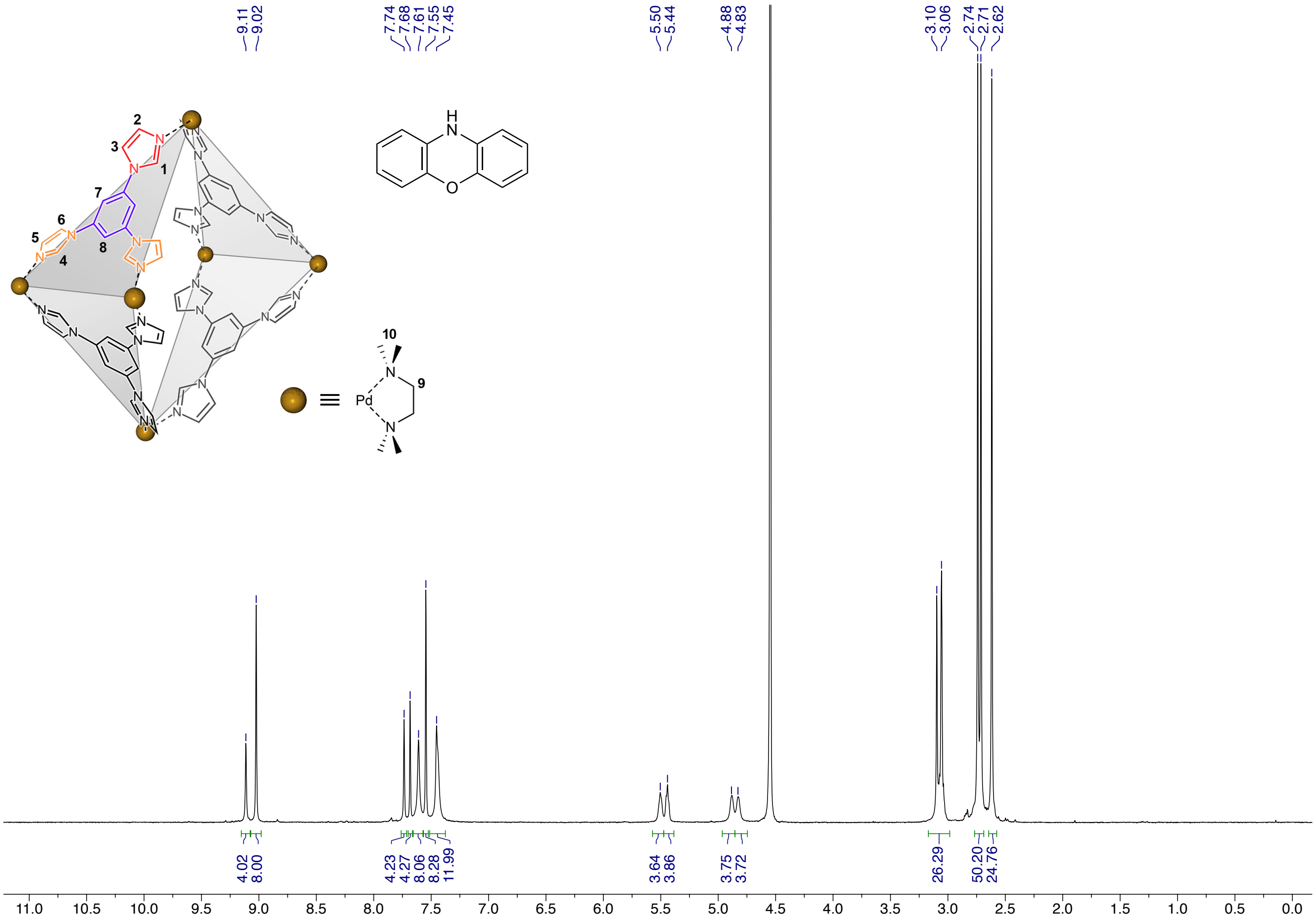


**Supplementary Fig. 58** $^1$H NMR spectrum of **6**$_2$⊂**1** (600 MHz, $D_2O$, 320 K).

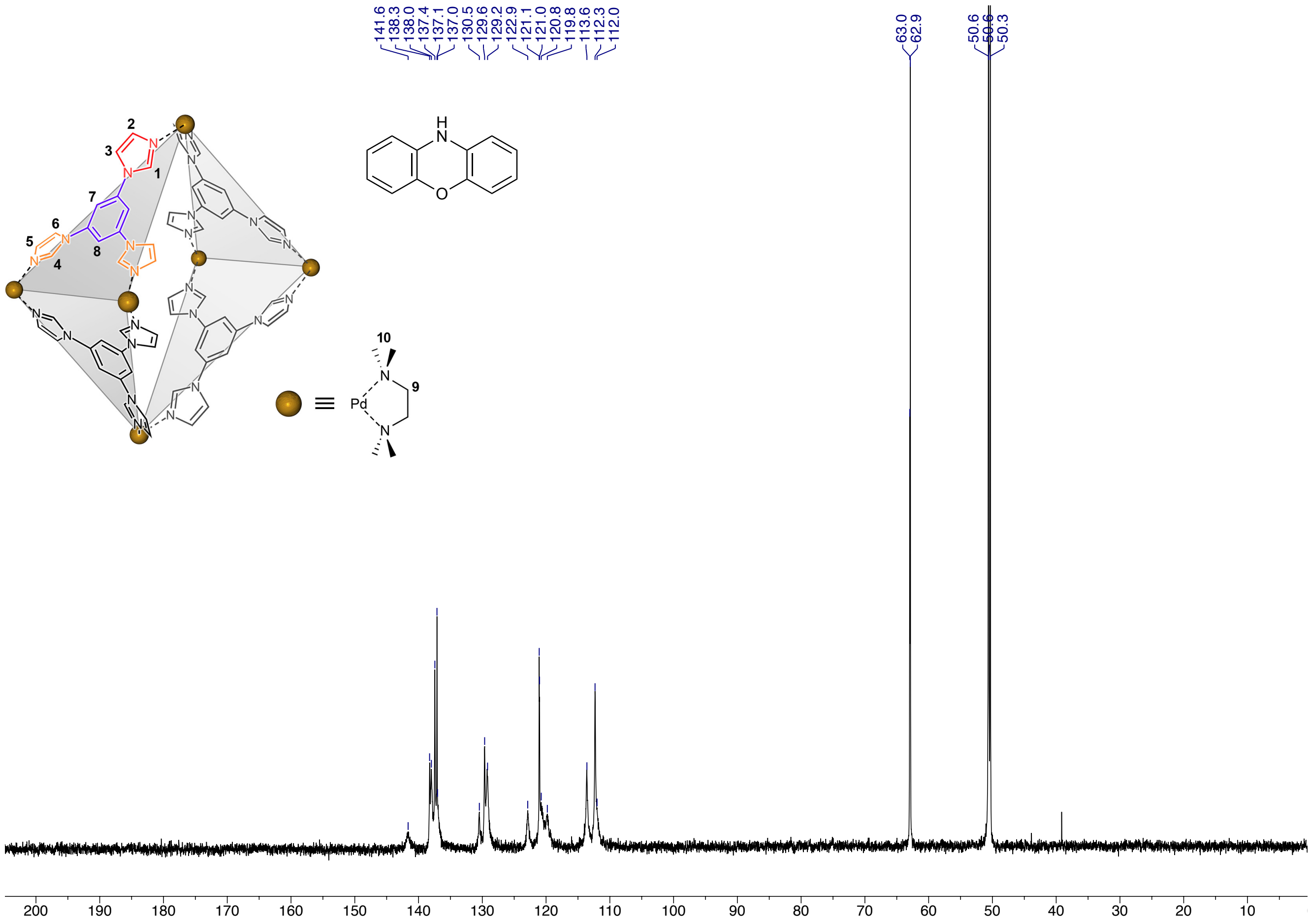


**Supplementary Fig. 59** $^{13}$C NMR spectrum of $\mathbf{6}_2\subset\mathbf{1}$ (151 MHz, $D_2O$, 320 K).

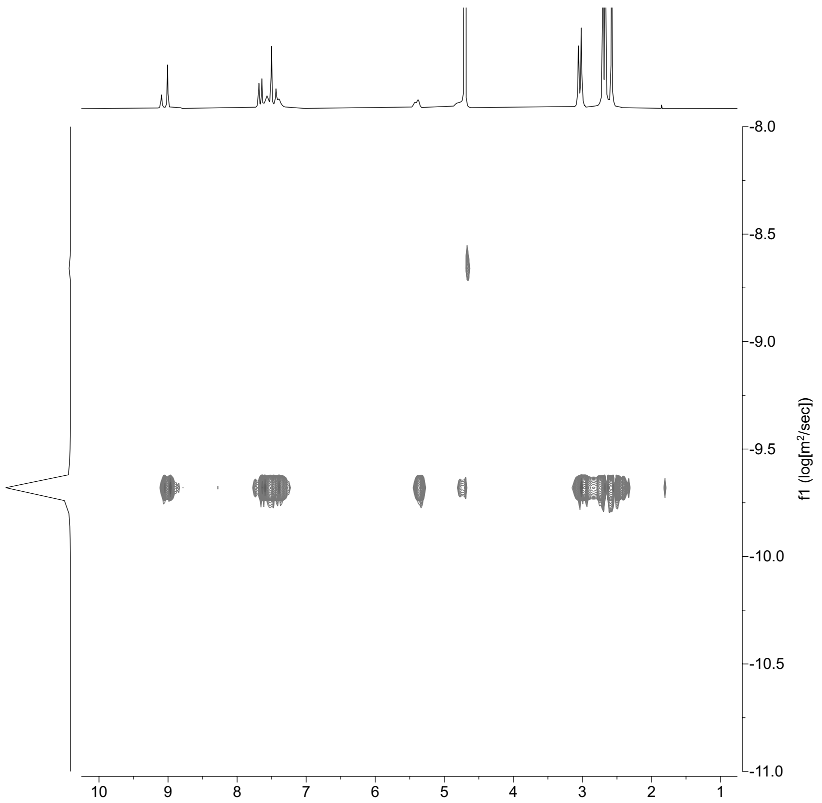


**Supplementary Fig. 60** $^{1}$H DOSY NMR spectrum of **6**$_2$⊂**1** (600 MHz, $D_2O$, 300 K). The signals at ~4.75 and ~5.4 ppm are due to encapsulated **6**.

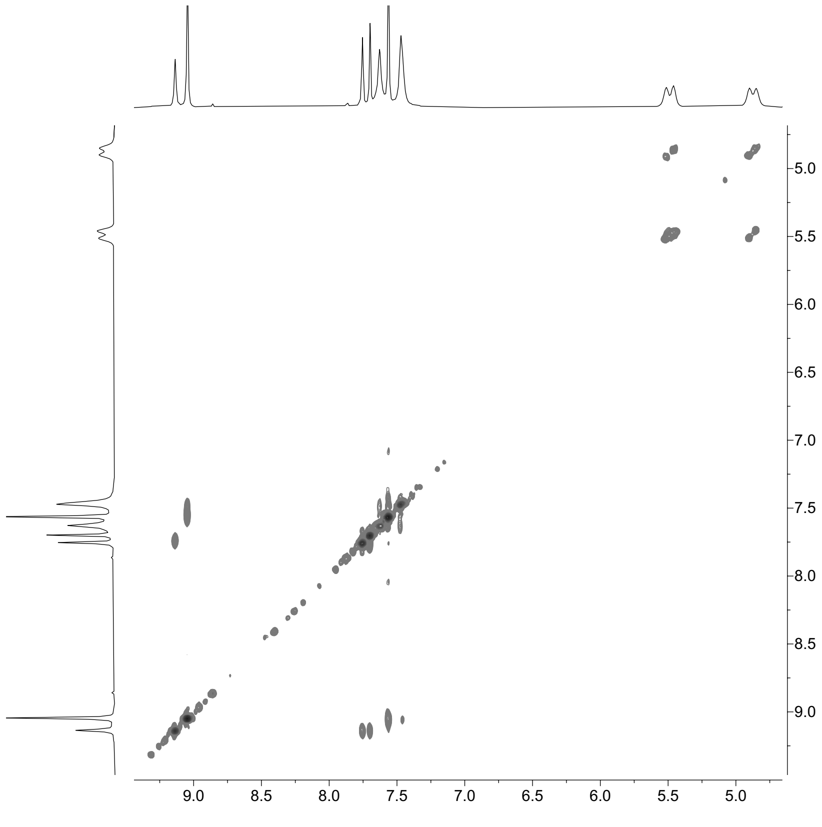

**Supplementary Fig. 61** Partial $^{1}$H–$^{1}$H COSY NMR spectrum of **6**$_2$⊂**1** (600 MHz, $D_2O$, 320 K).

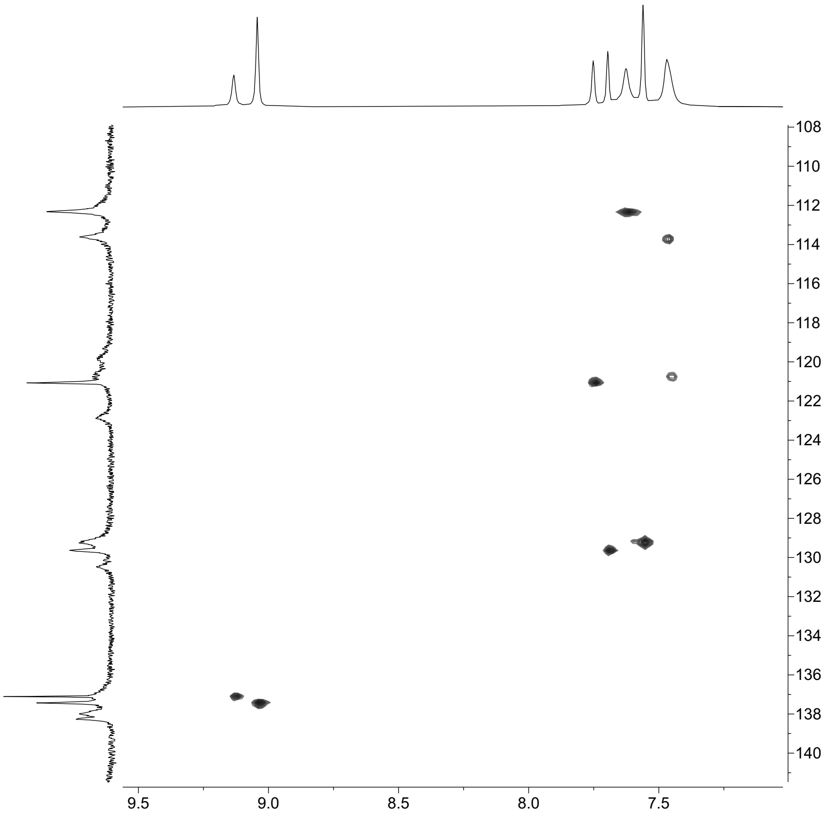


**Supplementary Fig. 62** Partial $^{1}H$–$^{13}C$ HSQC NMR spectrum of $\mathbf{6}_2{\subset}\mathbf{1}$ (600 MHz, $D_2O$, 320 K).

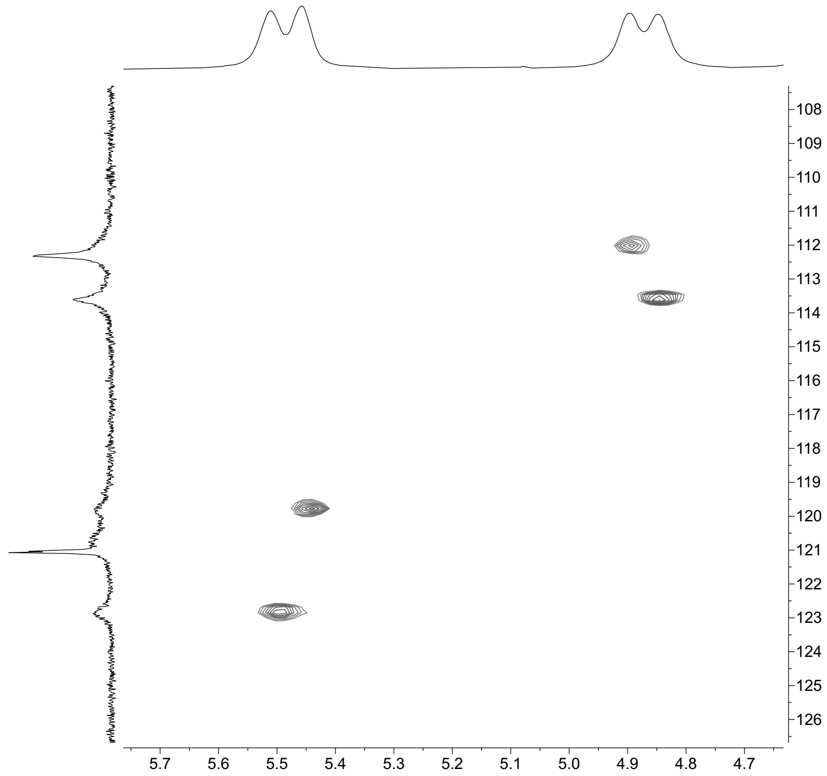


**Supplementary Fig. 63** Partial $^{1}H$–$^{13}C$ HSQC NMR spectrum of $\mathbf{6}_2{\subset}\mathbf{1}$ (600 MHz, $D_2O$, 320 K).

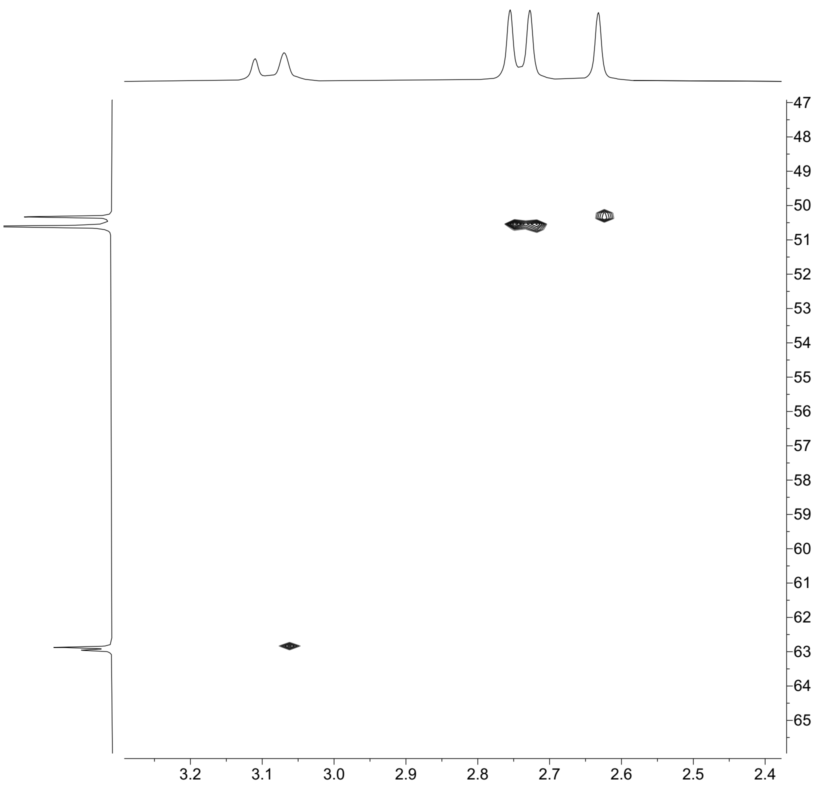


**Supplementary Fig. 64** Partial $^{1}H$–$^{13}C$ HSQC NMR spectrum of $\mathbf{6}_2{\subset}\mathbf{1}$ (600 MHz, $D_2O$, 320 K).

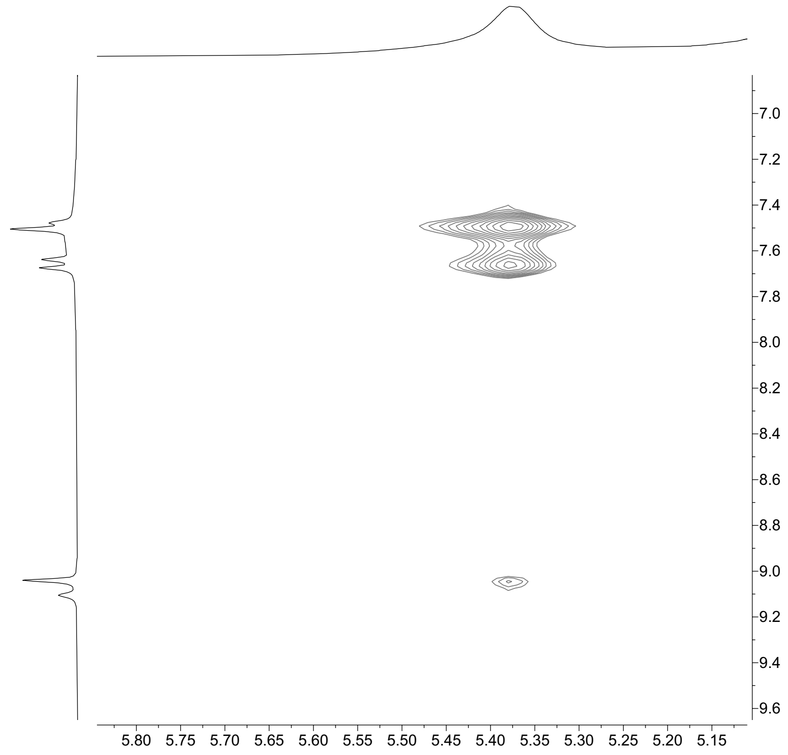


**Supplementary Fig. 65** Partial $^{1}H$–$^{1}H$ NOESY NMR spectrum of $\mathbf{6}_2{\subset}\mathbf{1}$ (600 MHz, $D_2O$, 278 K) (note that whereas the $^{1}H$ NMR spectrum of $\mathbf{6}_2{\subset}\mathbf{1}$ has a finer structure at 320 K, the nOe correlations are very weak).

## 6.2. Solid-state structure characterization

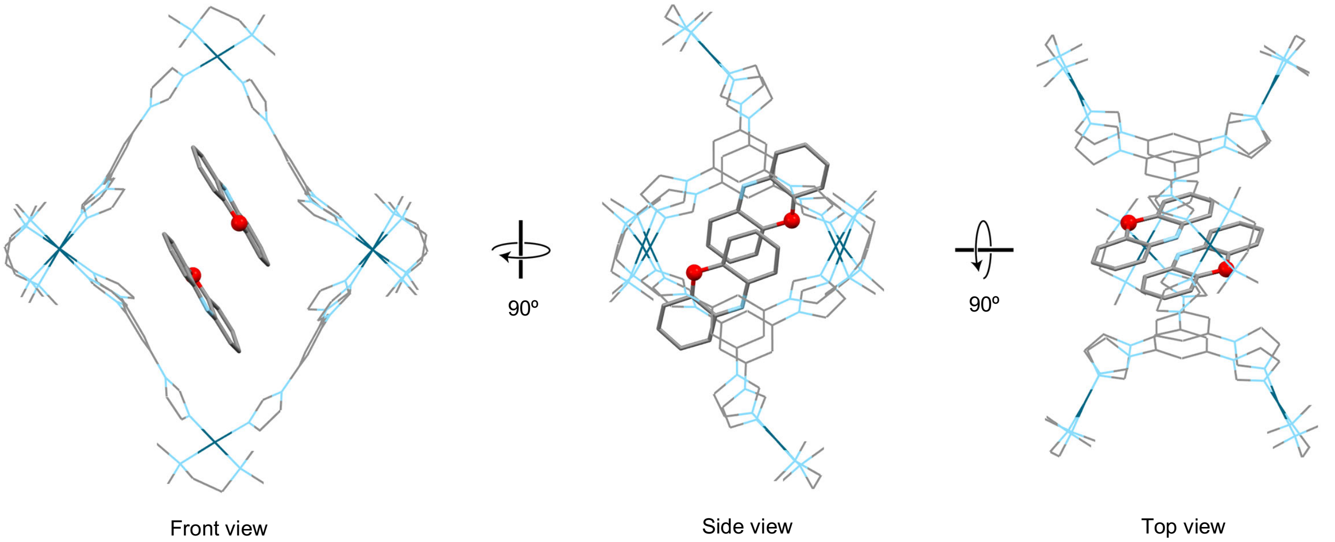


**Supplementary Fig. 66** X-ray structure of the **6**$_2$⊂**1** inclusion complex along three different viewing directions. It can be seen that encapsulated guests **6** assume a configuration different from that of encapsulated **2** and **4** (Supplementary Figs. 15 and 45, respectively; see also the corresponding CIF files). Nevertheless, the structure unambiguously confirms the 2:1 stoichiometry of the inclusion complex. The oxygen atoms are indicated as red spheres to highlight the arrangement of the two guests. Hydrogens, nitrates, and water molecules were omitted for clarity. Pd, dark-blue; C, gray; N, light-blue.

## 7. Crystal data and structure refinement data

| Species | $(2_2\subset 1)\cdot 2$ | $(2_2\subset 1)\cdot 2_4$ | 3 | $4_2\subset 1$ | $(4_2\subset 1)\cdot 4$ | $6_2\subset 1$ |
|---|---|---|---|---|---|---|
| CCDC No. | 2045607 | 2045611 | 2048454 | 2045608 | 2045606 | 2045610 |
| Formula* | $C_{132}H_{166}N_{48.63}O_{39.89}Pd_6$ | $C_{168}H_{180}N_{46}O_{66.24}Pd_6$ | $C_{84}H_{112}N_{36}O_{19}Pd_4$ | $C_{120}H_{156}N_{45}O_{41}Pd_6$ | $C_{132}H_{198}N_{48}O_{57}Pd_6$ | $C_{240}H_{323}N_{91.60}O_{102.60}Pd_{12}$ |
| Formula weight* | 3710.58 | 4541.74 | 2355.69 | 3523.29 | 4007.77 | 7409.65 |
| Crystal system | Triclinic | Triclinic | Monoclinic | Triclinic | Triclinic | Triclinic |
| Space group | $P\bar{1}$ | $P\bar{1}$ | $I2/a$ | $P\bar{1}$ | $P\bar{1}$ | $P\bar{1}$ |
| Crystal size (mm) | 0.14×0.05×0.01 | 0.20×0.10×0.02 | 0.39×0.05×0.03 | 0.12×0.12×0.04 | 0.07×0.04×0.02 | 0.28×0.12×0.06 |
| Crystal color and shape | Red plate | Reddish plate | Colorless needle | Blue plate | Greenish–blue plate | Colorless plate |
| Temperature (K) | 100 | 100 | 100 | 100 | 100 | 100 |
| Wavelength (Å) | 1.54184 | 1.54184 | 1.54184 | 1.54184 | 1.54184 | 0.71073 |
| a (Å) | 17.1715(2) | 17.1672(3) | 15.8229(6) | 16.6540(3) | 15.8348(3) | 19.5488(2) |
| b (Å) | 17.7476(2) | 17.6220(3) | 23.3987(11) | 17.4747(3) | 15.9249(3) | 20.6052(3) |
| c (Å) | 17.8897(2) | 22.1209(4) | 35.4491(14) | 18.1411(3) | 17.9726(2) | 25.6481(6) |
| α (º) | 80.2460(10) | 70.9633(16) | 90 | 86.5240(10) | 96.0127(12) | 107.457(2) |
| β (º) | 74.1860(10) | 86.3543(15) | 99.344(4) | 76.625(2) | 96.5684(11) | 95.0960(10) |
| γ (º) | 61.0800(10) | 73.4355(16) | 90 | 62.575(2) | 112.2020(15) | 93.9140(10) |
| Volume (Å$^3$) | 4586.86(10) | 6060.3(2) | 12950.4(9) | 4552.60(16) | 4114.23(11) | 9767.3(3) |
| Z | 1 | 1 | 4 | 1 | 1 | 1 |
| $\rho_{calcd}$ (g·cm$^{-1}$) | 1.343 | 1.244 | 1.208 | 1.285 | 1.618 | 1.260 |
| μ (mm$^{-1}$) | 5.313 | 4.200 | 4.955 | 5.323 | 6.048 | 0.619 |
| No. of reflections (unique) | 73549(17336) | 93592(24220) | 74187(13231) | 71102(18556) | 65672(16706) | 49130 (16706) |
| $R_{int}$ | 0.0489 | 0.0411 | 0.1656 | 0.0849 | 0.0663 | twin ref. |
| Completeness to θ (%) | 99.5 | 99.0 | 99.1 | 98.9 | 99.8 | 100 |
| Data / restraints / parameters | 17336 / 142 / 1068 | 24220 / 39 / 1426 | 13231 / 37 / 653 | 18556 / 211 / 1040 | 16706 / 78 / 1270 | 49130 / 240 / 2146 |
| Goodness-of-fit on $F^2$ | 1.004 | 1.107 | 1.597 | 1.085 | 1.045 | 1.050 |
| Final $R_1$ and $wR_2$ indices [$I$>2σ($I$)] | 0.0799, 0.2209 | 0.0704, 0.2028 | 0.1739, 0.2267 | 0.0835, 0.2491 | 0.0561, 0.1534 | 0.1201, 0.2979 |
| $R_1$ and $wR_2$ indices (all data) | 0.0877, 0.2363 | 0.0851, 0.2145 | 0.4072, 0.4326 | 0.0875, 0.2573 | 0.0607, 0.1595 | 0.1486, 0.3234 |

**Supplementary Table 1.** Crystallographic data. (*Derived from the crystal structure)

## Supplementary Note

### Proving the existence of (**2·4**)⊂**1**

The heterodimeric species (**2·4**)⊂**1** exists in dynamic equilibrium with homodimers $\mathbf{2}_2$⊂**1** and $\mathbf{4}_2$⊂**1**. To demonstrate the presence of (**2·4**)⊂**1**, we first recorded a UV-Vis spectrum of the mixture of $\mathbf{2}_2$⊂**1** and $\mathbf{4}_2$⊂**1**. Supplementary Fig. 67 shows the UV-Vis absorption spectra of $\mathbf{2}_2$⊂**1** (pink) and of $\mathbf{4}_2$⊂**1** (blue), obtained by mixing cage **1** with 2.0 equiv of **2** and **4**, respectively. A UV-Vis spectrum of a 1:1 mixture of $\mathbf{2}_2$⊂**1** and $\mathbf{4}_2$⊂**1** is shown in green. If this solution consisted of a mixture of $\mathbf{2}_2$⊂**1** and $\mathbf{4}_2$⊂**1**, its UV-Vis spectrum would be an average of the pink and the blue spectrum, which is denoted by a gray dashed line. However, the spectrum featured a single maximum located between those of $\mathbf{2}_2$⊂**1** and $\mathbf{4}_2$⊂**1**; this result indicates the formation of the heterodimeric species (**2·4**)⊂**1**.

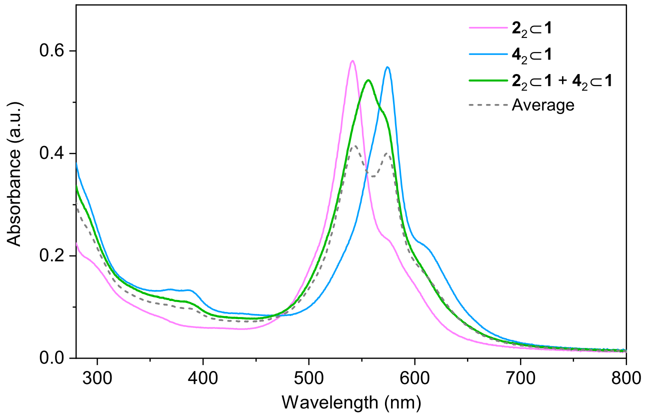


**Supplementary Fig. 67** UV-Vis absorption spectra of 7.6 μM aqueous solutions of $\mathbf{2}_2$⊂**1** (pink), $\mathbf{4}_2$⊂**1** (blue), and their 1:1 mixture (green). The gray dashed line denotes a weighted average of the pink and blue spectra.

We also studied our system by fluorescence spectroscopy. Supplementary Fig. 68a shows the emission spectra of **2** (pink) and **4** (blue), and their 1:1 mixture (green). The highly emissive component of the system is **2**, with an intense emission at ~580 nm. The emission of **4** (blue) is dominated by a minor contamination with **2**, as discussed above (p. 32). Despite the contamination contributing to only ~5 mol%, the emission intensity at ~580 nm is ca. three times higher than that at ~630 nm (due to **4**). The fluorescence spectrum of the 1:1 mixture of **2** and **4** (green) is a weighted average of those of free **2** and **4**, as expected.

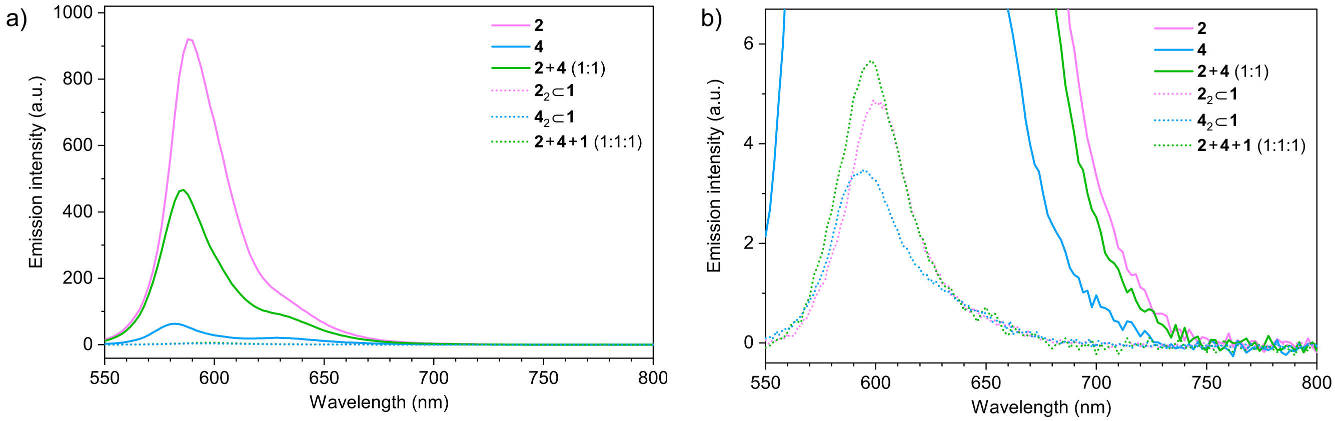


**Supplementary Fig. 68** Fluorescence spectra of free and encapsulated **2**, **4**, and their 1:1 mixture at two different intensity scales. Excitation wavelength, $\lambda_{exc}$ = 530 nm.

Supplementary Fig. 68b focuses on the low-intensity region of Supplementary Fig. 68a, showing the emission spectra of $\mathbf{2}_2\subset\mathbf{1}$ and $\mathbf{4}_2\subset\mathbf{1}$ (obtained by mixing cage **1** with 2 equiv of **2** and **4**, respectively), and of a 1:1 mixture of $\mathbf{2}_2\subset\mathbf{1}$ and $\mathbf{4}_2\subset\mathbf{1}$ (i.e., a mixture of $\mathbf{2}_2\subset\mathbf{1}$, $\mathbf{4}_2\subset\mathbf{1}$, and the heterodimer $(\mathbf{2}\cdot\mathbf{4})\subset\mathbf{1}$). The emission intensities of all three samples are similar, suggesting that they do not originate primarily from uncomplexed **2** or **4** – otherwise, large differences as in **a)** would be expected. Indeed, given the high association constants for both $\mathbf{2}_2\subset\mathbf{1}$ and $\mathbf{4}_2\subset\mathbf{1}$, $K_a \approx 10^{12}$, the fraction of uncomplexed **2** (or **4**) can be estimated as less than ~0.03%. Thus, if the inclusion complexes were not fluorescent, one would expect that the emission of **2** in the presence of 0.5 equiv **1** would be less than 1/30,000 of free **2**; however, it approaches 1/100 of that value. Importantly, the emission intensity from the $\mathbf{2}_2\subset\mathbf{1} + \mathbf{4}_2\subset\mathbf{1}$ mixture is higher than that of either of the individual components, indicating the formation of the $(\mathbf{2}\cdot\mathbf{4})\subset\mathbf{1}$, whose emission intensity is higher than that of the homodimers.

The existence of the $(\mathbf{2}\cdot\mathbf{4})\subset\mathbf{1}$ heterodimer is also evident from the NMR spectra of the $\mathbf{2}_2\subset\mathbf{1} + \mathbf{4}_2\subset\mathbf{1}$ mixture. If the mixture consisted of the two parent species, $\mathbf{2}_2\subset\mathbf{1} + \mathbf{4}_2\subset\mathbf{1}$, the guests would show six signals in the NMR spectrum (three from the encapsulated **2** and three from the encapsulated **4**; see the main text, Fig. 1c and 5a, respectively). However, we clearly observed, with the help of HSQC NMR (Supplementary Fig. 69), a set of six additional signals, three of which originate from **2** within $(\mathbf{2}\cdot\mathbf{4})\subset\mathbf{1}$, and the other three from **4** within $(\mathbf{2}\cdot\mathbf{4})\subset\mathbf{1}$.

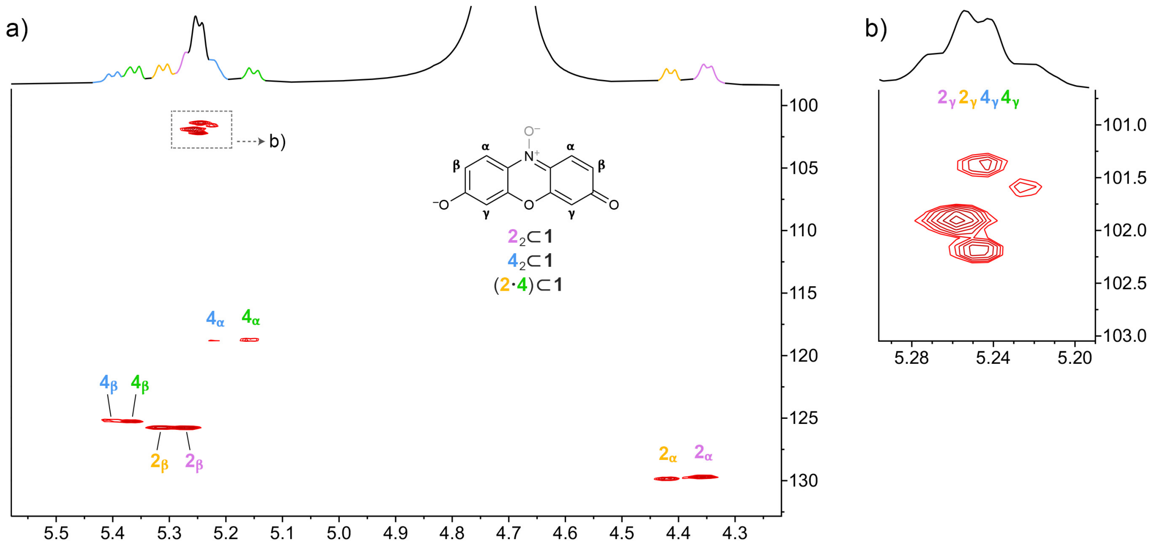


**Supplementary Fig. 69** Partial $^1$H–$^{13}$C HSQC NMR spectrum of a $\mathbf{2}_2\subset\mathbf{1} + \mathbf{4}_2\subset\mathbf{1}$ mixture (600 MHz, $D_2O$, 300 K). The peak assignment is based on the COSY spectrum of the same sample (Supplementary Fig. 70) and on the $^1$H NMR spectra of the two homodimers.

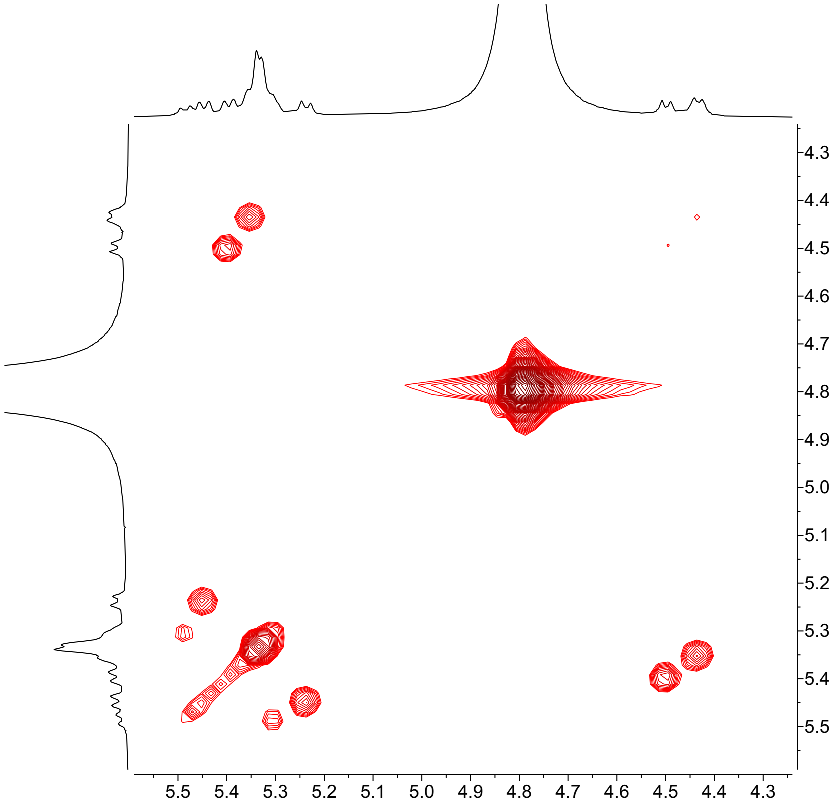


**Supplementary Fig. 70** Partial $^{1}$H–$^{1}$H COSY NMR spectrum of a **2**$_2$⊂**1** + **4**$_2$⊂**1** mixture (600 MHz, $D_2O$, 300 K).

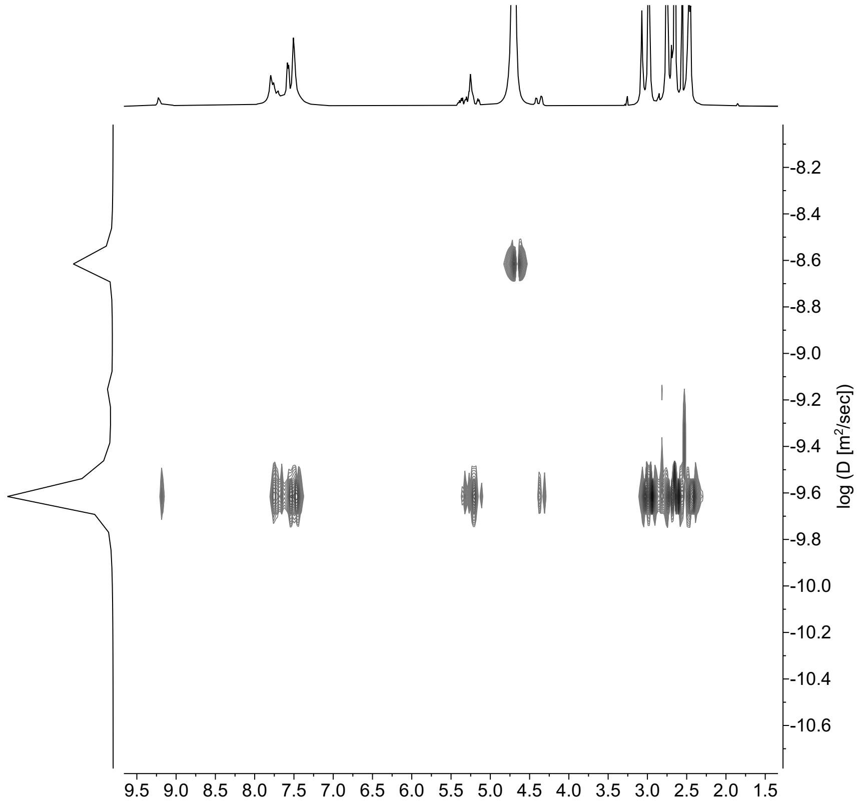


**Supplementary Fig. 71** $^{1}$H DOSY NMR spectrum of a **2**$_2$⊂**1** + **4**$_2$⊂**1** mixture (600 MHz, $D_2O$, 300 K).

## Supplementary References


1. Canton, M. et al. Improving fatigue resistance of dihydropyrene by encapsulation within a coordination cage. *J. Am. Chem. Soc.* **142**, 14557–14565 (2020).
2. Bueno Alejo, C. J., Fasciani, C., Grenier, M., Netto-Ferreira, J. C. & Scaiano, J. C. Reduction of resazurin to resorufin catalyzed by gold nanoparticles: dramatic reaction acceleration by laser or LED plasmon excitation. *Catal. Sci. Technol.* **1**, 1506–1511 (2011).
3. Zhou, X., Xu, W., Liu, G., Panda, D. & Chen, P. Size-dependent catalytic activity and dynamics of gold nanoparticles at the single-molecule level. *J. Am. Chem. Soc.* **132**, 138–146 (2010).
4. Bigley, M. J., Radigan, K. J. & Nathan, L. C. Aliphatic di-N-oxide complexes; cobalt(II), nickel(II), and chromium(III) perchlorate complexes of N,N,N',N'-tetramethylethylenediamine-N,N'-dioxide. *Inorg. Chim. Acta* **16**, 209–212 (1976).
5. Read, C. D. G., Moore, P. W. & Williams, C. M. *N*,*N*,*N*′,*N*′-Tetramethylenediamine dioxide (TMEDAO2) facilitates atom economical/open atmosphere Ley–Griffith (TPAP) tandem oxidation-Wittig reactions. *Green Chem.* **17**, 4537–4540 (2015).
6. Moore, P. W. et al. Selectivity modulation of the Ley–Griffith TPAP oxidation with *N*-oxide salts. *Eur. J. Org. Chem.* **2016**, 3401–3407 (2016).
