# Supplementary material for: Encapsulation within a coordination cage modulates the reactivity of redox-active dyes
Source: Commun Chem. 2022 Mar 30;5:44. doi: 10.1038/s42004-022-00658-8 (PMC9814915; doi:10.1038/s42004-022-00658-8)
Supplement: Supplementary file 2 — Description of Additional Supplementary Files [file 42004_2022_658_MOESM2_ESM.pdf]

Description of Additional Supplementary Files

File Name: Supplementary Data 1

Description:

- “(22c1)2.cif”: Single-crystal X-ray structure of ($\mathbf{2}_2\subset\mathbf{1}$)·**2**
- “(22c1)24.cif”: Single-crystal X-ray structure of ($\mathbf{2}_2\subset\mathbf{1}$)·$\mathbf{2}_4$
- “3.cif”: Single-crystal X-ray structure of **3**
- “(42c1)4.cif”: Single-crystal X-ray structure of ($\mathbf{4}_2\subset\mathbf{1}$)·**4**
- “42c1.cif”: Single-crystal X-ray structure of $\mathbf{4}_2\subset\mathbf{1}$
- “62c1.cif”: Single-crystal X-ray structure of $\mathbf{6}_2\subset\mathbf{1}$
- “DFT1.cif”: Energy-optimized structure of the $\mathbf{2}\subset\mathbf{1}$ inclusion complex
- “DFT2.cif”: Energy-optimized structure of the $\mathbf{2}\subset\mathbf{1}'$ inclusion complex
